# Glass formation of metal organic–inorganic complexes

**DOI:** 10.1093/nsr/nwag349

**Published:** 2026-06-08

**Authors:** Tianzhao Xu, Zhencai Li, Jia-Xin Wu, Zihao Wang, Hanmeng Zhang, Huotian Zhang, Lars R Jensen, Kenji Shinozaki, Feng Gao, Haomiao Zhu, Ivan Hung, Zhehong Gan, Jinjun Ren, Zheng Yin, Ming-Hua Zeng, Yuanzheng Yue

**Affiliations:** Department of Chemistry and Bioscience, Aalborg University, Aalborg 9220, Denmark; Department of Chemistry and Bioscience, Aalborg University, Aalborg 9220, Denmark; School of Chemistry and Pharmaceutical Sciences, State Key Laboratory for Chemistry and Molecular Engineering of Medicinal Resources, Guangxi Normal University, Guilin 541004, China; Xiamen Research Center of Rare Earth Materials, Haixi Institutes, Chinese Academy of Sciences, Xiamen 361021, China; Shanghai Institute of Optics and Fine Mechanics, Chinese Academy of Sciences, Shanghai 201800, China; Department of Physics, Chemistry, and Biology (IFM), Linköping University, Linköping 583 30, Sweden; Department of Materials and Production, Aalborg University, Aalborg 9220, Denmark; Research Institute of Core Technology for Materials Innovation, National Institute of Advanced Industrial Science and Technology (AIST), Ikeda 563-8577, Japan; Department of Physics, Chemistry, and Biology (IFM), Linköping University, Linköping 583 30, Sweden; Xiamen Research Center of Rare Earth Materials, Haixi Institutes, Chinese Academy of Sciences, Xiamen 361021, China; National High Magnetic Field Laboratory, Tallahassee, FL 32310, USA; National High Magnetic Field Laboratory, Tallahassee, FL 32310, USA; Shanghai Institute of Optics and Fine Mechanics, Chinese Academy of Sciences, Shanghai 201800, China; School of Chemistry and Pharmaceutical Sciences, State Key Laboratory for Chemistry and Molecular Engineering of Medicinal Resources, Guangxi Normal University, Guilin 541004, China; School of Chemistry and Pharmaceutical Sciences, State Key Laboratory for Chemistry and Molecular Engineering of Medicinal Resources, Guangxi Normal University, Guilin 541004, China; Department of Chemistry and Bioscience, Aalborg University, Aalborg 9220, Denmark

**Keywords:** ligand-tuning approach, hydrogen-bonded network, glass formation, photoluminescence

## Abstract

Some metal organic–inorganic complexes (MOICs) are known for their glass-forming ability. However, the composition range of glass-forming MOICs is rather limited as the mechanism of MOIC glass formation has not been well understood. Here, we uncover the structural factors controlling MOIC glass formation and thereby develop new MOIC glass formers. MOIC glass formation relies on the creation of a hydrogen-bonded structural network. By properly choosing metal centers, anions, and organic ligands of MOICs, a supramolecular structural network can be constructed, thereby allowing property modulation. Furthermore, MOIC systems with mixed crystals are generated by ligand-mixing and then vitrified by melt-quenching. The glass-transition temperature (*T*_g_) of the derived glasses can be linearly tuned through the substitution of benzimidazole for imidazole. Interestingly, vitrification led to disordering of hydrogen-bonded networks in MOICs at short-, medium-, and long-range scales. This work enables the expansion of the composition range of MOIC glasses with various functionalities.

## Teaser

This work developed a series of metal organic-inorganic complexes (MOICs), which can be vitrified to glass state, and revealed their glass formation mechanism.

## INTRODUCTION

Metal complex (MC) crystals, composed of metal ions or atoms coordinated with organic or inorganic ligands, represent the cornerstone of coordination chemistry, a field that has been studied for over a century [1]. Recently, melt-quenching of MC crystal systems has led to the emergence of metal-organic framework (MOF) glasses, coordination polymer (CP) glasses, and metal organic–inorganic complex (MOIC) glasses, and these glasses exhibit zero- to three-dimensional disordered network structures [2–4]. Due to their structural diversity, high tunability, and designability, MC crystals can be potentially applied in various fields, including gas separation [5], lithium-ion batteries [6], and luminescent host materials [4]. Compared to MC crystals, the upscaling applications of melt-quenched MC glasses are enabled by their bulk nature and enhanced processability [3,7,8]. Tracking the structural evolution of MOFs and CPs during glass formation offers deeper insight into the nature of melt-quenched MC glasses, thereby enabling the development of new glass-forming MCs [2,9].

Within MCs, some zeolitic imidazolate frameworks (ZIFs), a subclass of MOFs, exhibit an ultra-high glass-forming ability (GFA) and a tetrahedral network structure analogous to that of SiO_2_ [10]. Various ligands such as solvents [11], phosphates [12], and halides [13] can be introduced into ZIFs to generate CPs or MOICs. This introduction enables partial substitution of weaker interactions (e.g., hydrogen bonds or weak coordination) for the coordination bonds in ZIFs, thereby reducing the melting energy barrier for the formation of bulk MC glasses. The processability and structural flexibility of the glasses allow for their applications, such as luminescent hosts [14], room-temperature phosphorescence [15], and gas separation membranes [16]. Past investigations have mostly focused on some properties of hydrogen-bonded MOIC glasses, but their underlying glass-formation mechanisms have not been elucidated. This is because the structure evolution during the vitrification of MOIC crystals has not been revealed. The lack of detailed structural characterizations of MOIC glasses further constrains the development of new glass-forming systems. Exploring the structural evolution of MOIC crystals during vitrification is critically important for establishing a universal strategy for the synthesis of new types of hybrid MOIC glasses. Recently, an experimental approach was employed to identify complex disorder motifs in hybrid metal halide glasses, thereby providing insight into their glass formation mechanism [17].

In the present work, MOIC crystals ZnCl_2_(HIm)_2_ (MOIC-4) and ZnCl_2_(HbIm)_2_ (MOIC-7) (HIm: imidazole; HbIm: benzimidazole) were designed and synthesized via a slow-evaporation method. MOIC-4 and MOIC-7 feature Zn-centered tetrahedral units analogous to those in ZIF-4 and ZIF-7, respectively [18], but with the imidazolate-based linkers (Im: imidazolate; bIm: benzimidazolate) replaced with the hydrogen-bond acceptor (Cl^−^) and hydrogen-bond donor (HIm/HbIm). The supramolecular structures of the two MOICs are constructed via hydrogen bonding. In particular, the HbIm ligands in MOIC-7 contribute extensive *π-π* interactions to the overall structure. A binary eutectic MOIC system was obtained through ligand-mixing synthesis, causing reduced melting points (*T*_m_) and tunable glass-transition temperatures (*T*_g_). The structural evolution of MOIC crystals during melting was characterized by *in situ* X-ray diffraction (XRD). The structures of both crystalline and glassy MOICs were investigated by *ex situ* XRD, Fourier transform infrared (FT-IR) transmittance, Raman spectroscopy, high-energy synchrotron XRD, and solid-state nuclear magnetic resonance (NMR) spectroscopy. Both the *T*_m_ of MOICs and the *T*_g_ of MOIC glasses were measured using differential scanning calorimetry (DSC). In addition, the design strategy involved multi-level modulations of the metal nodes (Cu/Co substituting Zn) and anions (SCN^−^ substituting Cl^−^). The photonic properties of MOIC-7 crystals and glasses were determined to reveal the correlation between structural design and material performance. Our work offers insights into the vitrification mechanism of MOIC crystals and a new avenue for developing novel functional MOIC glasses.

## RESULTS AND DISCUSSION

### Characteristics of MOIC crystals and glasses

**Figure 1.**
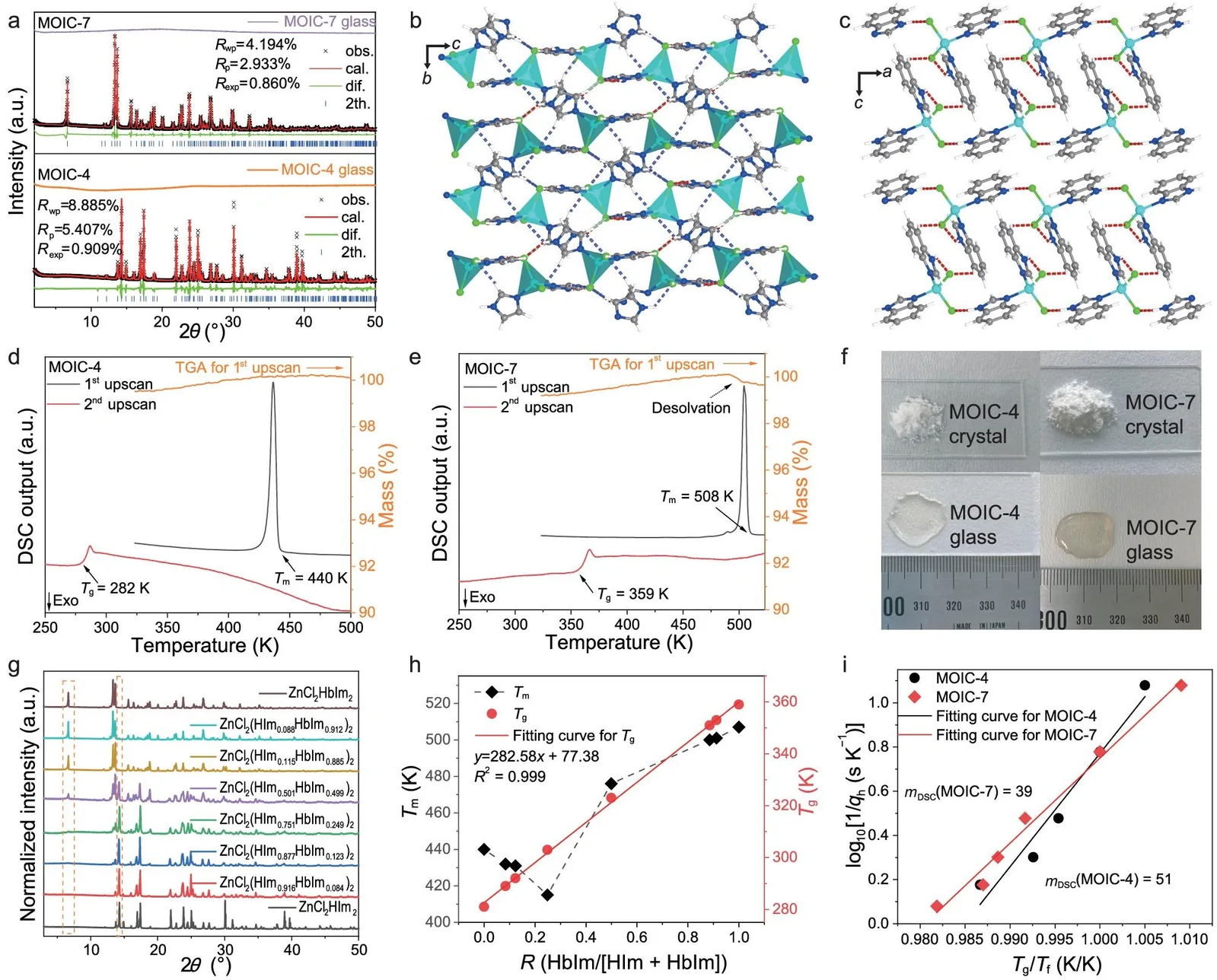
(a) X-ray diffraction (XRD) patterns of the synthesized MOIC-4 and MOIC-7 crystals and glasses, and refinement result of the two crystals. (b, c) The crystal structure of MOIC-4 (b) and MOIC-7 (c). (d, e) Differential scanning calorimetry (DSC) and thermogravimetry (TG) curves of MOIC-4 (d) and MOIC-7 (e). (f) The photos of the synthesized MOIC-4 and MOIC-7 crystals and their corresponding glasses. (g) XRD patterns of the binary eutectic system. (h) Relationships between the molar ratio *R* (HbIm/[HIm + HbIm]) and the *T*_m_ (±2 K) and *T*_g_ (±1 K) values for the binary eutectic series. (i) Linear dependence of the reciprocal heating rate (log_10_1/*q*_h_) on the *T*_g_-scaled fictive temperature (*T*_g_/*T*_f_) for MOIC-4 glass and MOIC-7 glass. The slope of the linear fit at *T*_g_ is the calorimetric fragility index (*m*_DSC_).

Two Zn-based MOICs, MOIC-4 (ZnCl_2_(HIm)_2_) and MOIC-7 (ZnCl_2_(HbIm)_2_), are synthesized using the slow evaporation method (see Supporting Information). In the supramolecular structure of MOICs, the imidazole ligands primarily enable hydrogen bonding, whereas the benzimidazole ligands facilitate *π-π* interactions, giving rise to fluorescence. To characterize the crystalline structure, the XRD patterns of the two MOIC crystals are collected and refined, as shown in Fig. 1a. From the XRD results, MOIC-4 is identified to be a monoclinic phase [19], whereas MOIC-7 is a triclinic phase [20]. To uncover the supramolecular interaction within the crystal structure, Density Functional Theory (DFT) calculations are carried out. All input files for the CP2K program are generated using the Multiwfn program [21,22]. The crystal structures of both MOICs are assembled and stabilized by intermolecular interactions, including hydrogen bonding and *π-π* stacking (Fig. 1b and c). Specifically, two types of [ZnCl_2_(HIm)_2_] tetrahedra in MOIC-4 are interdigitated and linked by N-H…Cl hydrogen bonds to form zigzag chains along the $[ {10\!\!\!\bar{\,\,\,1}} ]$ direction. These chains further extend through N-H…Cl and C-H…Cl hydrogen bonds into two-dimensional hydrogen-bonded layers in the *ac* plane. The weak hydrogen bonds and *π-π* interactions govern the stacking of the hydrogen-bonded layers into an ABAB arrangement, thereby resulting in the supramolecular structure (Figs S1 and S2). In MOIC-7, [ZnCl_2_(HbIm)_2_] tetrahedra are linked by N-H…Cl hydrogen bonds to form one-dimensional chains along the [100] direction. These chains extend through N-H…Cl hydrogen bonds along the *b* axis into a two-dimensional layer, which is interdigitated with another two-dimensional layer via interlayer C-H…Cl hydrogen bonds and *π-π* interactions. Finally, the interdigitated layers are stacked through *π-π* interactions, resulting in the formation of the supramolecular structure (Table S1 and Figs S3–S5).

Figure 1d and e shows the enthalpic responses of MOIC-4 and MOIC-7 during DSC upscan, respectively. The endothermic signal in the first upscan is ascribed to melting, whereas the endothermic event observed during the second upscan is attributed to the glass transition. During this process, MOIC-7 crystal exhibits a slight mass loss of about 0.5%, attributed to the release of the residual solvent (acetone) trapped within the as-synthesized crystal. No further mass loss is detected over three consecutive upscan-downscan cycles for MOIC-7 (Fig. S6), indicating the total desolvation of acetone during the first upscan. The offset of the melting peak (*T*_m_) is determined to be 440 and 508 K for MOIC-4 and MOIC-7, respectively [23]. The higher *T*_m_ of MOIC-7 hints at the following scenario. Due to its larger size, the HbIm ligand may cause a higher degree of steric hindrance, while the interdigitated arrangement provides greater structural stability than the stacked counterpart. These factors suppress the mobility of structural units and hence require a higher temperature to break down the network for melting. The transparent MOIC-4 and MOIC-7 glasses are produced by melting the MOIC crystals at temperatures above their respective *T*_m_ in air for 1 min and subsequently quenching the resultant melt to room temperature (Fig. 1f). No sharp XRD Bragg peak is observed in the melt-quenched samples (Fig. 1a), indicating their glassy nature. *T*_g_ values of MOIC-4 and MOIC-7 glasses are determined to be 282 and 359 K, respectively (Fig. 1d and e). The higher *T*_g_ of MOIC-7 glass is ascribed to the increase of the steric hindrance in the structural network, caused by HbIm. The increased steric hindrance makes the structural network more rigid, and hence a higher temperature is required to induce the glass transition.

A series of MOIC crystals with different *T*_m_ values was synthesized by varying the ligand ratio. To determine the composition of the crystals, solution-state ^1^H NMR spectra of the samples were collected (Figs S7–S12). The results show that the compositions of the derived series of crystals almost match the designed ones. XRD patterns and refinement analysis (Fig. 1g and Fig. S13) reveal that all samples are physical mixtures of MOIC-4 and MOIC-7. However, the molar ratio between MOIC-4 and MOIC-7 determined by XRD refinement for each of the six non-endmember compositions deviates from the theoretically designed value. This deviation might be attributable to the imperfectly grown crystals. To study the compositional impact on the characteristic temperatures of the binary MOIC series, their *T*_g_ and *T*_m_ values (Fig. 1h and Table S2) are determined from the DSC upscan curves (Figs S14–S19). In the binary MOIC series, each of the six samples containing both HIm and HbIm (Figs S14–S19) exhibits a broader melting peak than the two endmembers (MOIC-4 and -7) (Fig. 1d and e). This contrast implies that the melting peak of the non-endmembers is likely a result of the partial overlap of two melting peaks. This is a typical eutectic behavior.

Upon cooling, the eutectic system melts preferentially undergo vitrification rather than crystallization. Notably, MOIC-4 glass undergoes rapid crystallization upon exposure to moisture. Thus, to inhibit crystallization, moisture must be suppressed. In other words, MOIC glass should be utilized in a closed system without moisture. In addition, the binary eutectic glasses with *T*_g_ values higher than room temperature (298 K) exhibit excellent ambient stability against crystallization. The high-*T*_g_ MOIC glasses have been developed in our parallel work.

The *T*_g_/*T*_m_ ratio is an indirect measure of the GFA of a liquid. A higher *T*_g_/*T*_m_ ratio implies a high GFA [10]. Figure S20 reveals the correlation between the *T*_g_/*T*_m_ ratio and the molar ratio of *R* = HbIm/(HIm + HbIm) in the binary MOIC system. It is seen that the eutectic system with *R* = 0.25 has the highest *T*_g_/*T*_m_ ratio, implying its highest GFA. This could be attributed to its abundant hydrogen-bonding motifs formed between structural units. In addition, it is observed that its melting peak is broader than those of MOIC-4 and -7 (see Fig. 1d, e, and Fig. S16). This could be because the eutectic system has higher degrees of lattice dislocations, high chemical heterogeneity in crystals, and a mixed-ligand effect compared with the two endmembers [24]. The linear increase of *T*_g_ values with increasing *R* implies that the replacement of HbIm for HIm results in larger Zn-ligand tetrahedra, thereby enhancing the steric hindrance of the structural network in the glass [10].

Figures S21 and S22 show the DSC upscan curves of MOIC-4 and MOIC-7 glasses, measured at different upscan rates (*q*_h_) and corresponding to their prior cooling rates. The onset temperature of the glass transition peak is defined as the fictive temperature (*T*_f_). *T*_g_ was determined as the *T*_f_ measured at the upscan rate of 10 K/min. Figure 1i demonstrates the dependence of the reciprocal rate of heating log (1/*q*_h_) on the *T*_g_-scaled *T*_f_ (*T*_g_/*T*_f_) for MOIC-4 (black color) and MOIC-7 (red color) glasses. The slope of the log(1/*q*_h_) versus *T*_g_/*T*_f_ curve at *T*_g_ is defined as the calorimetric fragility index (*m*_DSC_) [25]. *m*_DSC_ is found to be about 51 for MOIC-4 glass and about 39 for MOIC-7 glass (Fig. 1i). From *m*_DSC_, the kinetic fragility index *m*_vis_, where vis refers to viscosity, can be derived through the equation:


(1) \begin{eqnarray*}
{m}_{{\mathrm{vis}}} = 1.289\left( {{m}_{{\mathrm{DSC}}} - {m}_0} \right) + {m}_0,
\end{eqnarray*}

where *m*_0_ denotes the fragility (14.97) of an ideally strong glass. Thus, the values of *m*_vis_ for MOIC-4 and MOIC-7 glasses are found to be ∼62 and 46, respectively. From *m*_vis_ and *T*_g_ values, the temperature dependence of the viscosity for both glass systems can be determined through the simplified version of the Mauro-Yue-Ellison-Gupta-Allan (MYEGA) model [25,26]:


(2) \begin{eqnarray*}
{\log }_{10}\eta \left( T \right) &=& - 3 + \ 15\frac{{{T}_{\rm g}}}{T}\\
&&\times \, {\mathrm{exp}}\!\left[ {\left( {\frac{{{m}_{\rm vis}}}{{15}} - 1} \right)\left( {\frac{{{T}_{\rm g}}}{T} - 1} \right)} \right].\\
\end{eqnarray*}

The derived viscosity-temperature curves (Figs S23 and S24) are useful for designing the melting and processing conditions of glass materials. They also provide insight into the dynamic behavior of the studied MOIC glasses. In general, a higher *m*_vis_ implies a faster slow-down process of a glass-forming liquid during cooling toward *T*_g_.

Compared to MOIC-4, MOIC-7 involves stronger hydrogen-bonding in the structural network, which gives a lower topological degree of freedom in structural rearrangement during the liquid-to-glass transition. This is why MOIC-7 liquid exhibits a lower liquid fragility and a higher GFA (e.g., higher *T*_g_/*T*_m_ ratio) than MOIC-4 (Fig. S20). Thus, MOIC-7 melt has a better workability (i.e., a broader working temperature range) than MOIC-4 melt when it is processed to glass products. In addition, the hydrogen bond strength in MOIC systems is lower than the coordination bond strength in ZIF systems. This is one of the reasons why MOIC glass exhibits a much lower *T*_g_ value and a lower GFA than ZIF glasses [10]. As is known, the effective number of intact constraints per atom is positively related to both the counted number of constraints per atom [27] and the field strength between the central atom and its coordinated atoms [28]. Obviously, since the field strength between H and Cl in a MOIC system is lower than that between Zn and N in a ZIF system, the effective number of intact constraints per atom in the former system is lower than that of the latter one. This also explains why the former exhibits a higher fragility index and lower GFA than the latter. However, the quantitative relationships among topological constraints, liquid fragility, and GFA remain to be established in future work.

### Structural evolution of MOIC crystals

**Figure 2.**
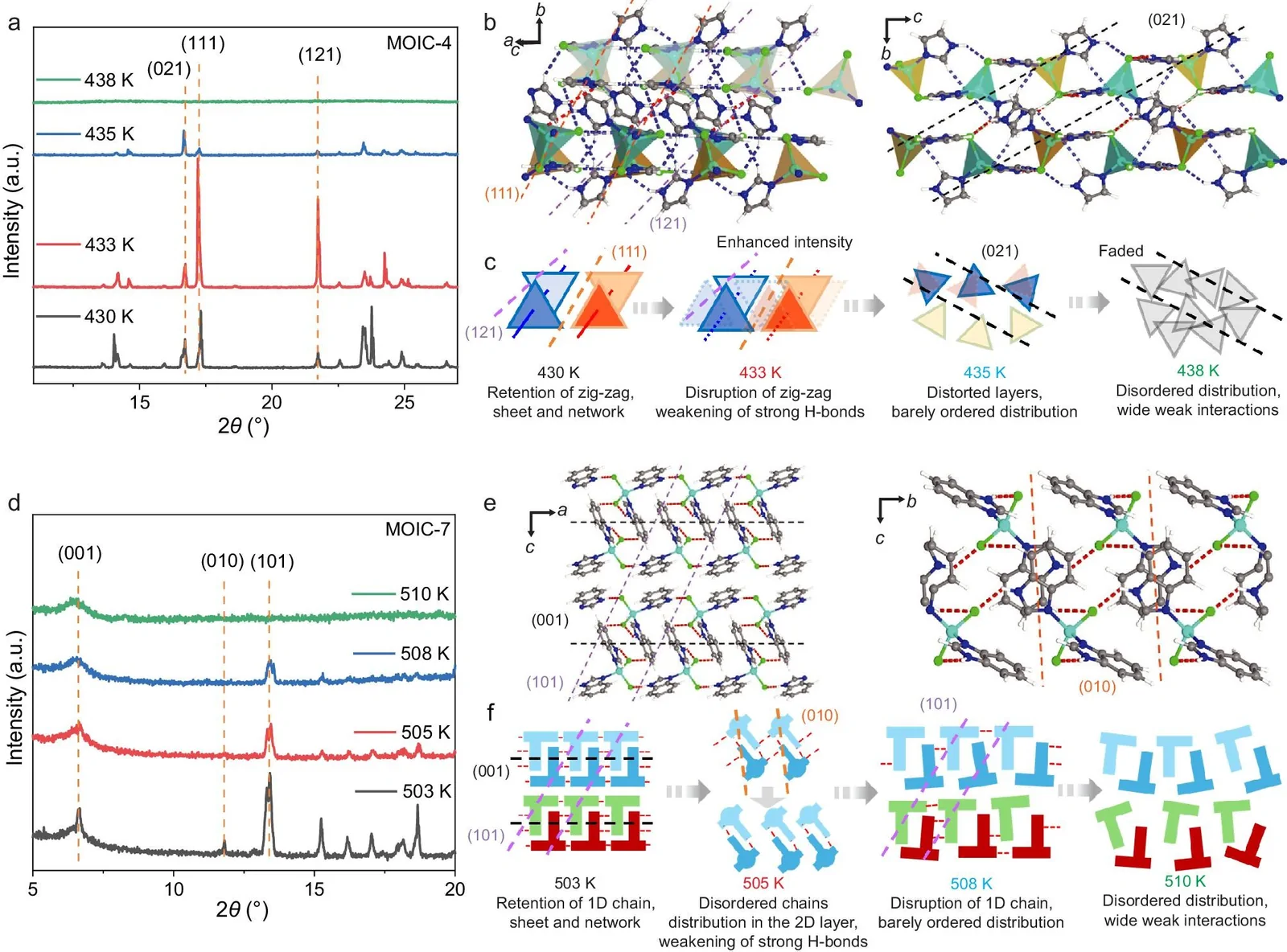
(a) Variable-temperature XRD patterns of MOIC-4 during melting. (b) Crystal planes of MOIC-4. (c) Schematic diagram of the structural evolution of MOIC-4 during melting. (d) Variable-temperature XRD patterns of MOIC-7 during melting. (e) Crystal planes of MOIC-7. (f) Schematic diagram of the structural evolution of MOIC-7 during melting.

To investigate the structural evolution of MOIC crystals during vitrification, an *in-situ* XRD experiment was performed on both MOIC-4 and MOIC-7 crystals. Variable-temperature XRD patterns of MOIC-4 (Fig. S25) and MOIC-7 (Fig. S26) show that their crystal structures remain intact prior to melting, without phase transitions detected. In MOIC-4, the peaks corresponding to the (121) and (111) planes increase in intensity at the early stage of melting and then vanish abruptly, whereas the peak corresponding to the (021) plane remains until complete melting (Fig. 2a). In the crystal structure of MOIC-4 (Fig. 2b), the zigzag chains formed by tetrahedra linked through the strong N-H…Cl hydrogen bonds lengthen within the (121) and (111) planes. Therefore, the observed evolutions of the peak intensity can be attributed to changes in the chains and their stacking arrangements. In contrast, the (021) plane intersects the zigzag chains, and this plane involves both weak hydrogen bonds and *π-π* interactions between adjacent chains. Upon melting at 433 K, the weakening of strong hydrogen bonds causes distortion and slippage of the zigzag chains, and thus more Zn atoms stay on the (021) plane, thereby enhancing the intensity of the chain-related reflections. Upon heating to 435 K, the N-H…Cl bonds break and the zigzag chains rapidly disintegrate, leaving only weak interactions between adjacent molecules to maintain the crystal structure. Further heating to 438 K leads to the loss of intermolecular correlations, resulting in the molten state of MOIC-4 (Fig. 2c).

In MOIC-7, the intensities of all diffraction peaks decrease as temperature increases to 510 K. Using the background-subtracted integrated areas in each diffraction pattern as a reference, the (010) reflection is the first to vanish (Fig. 2d). In the crystal structure of MOIC-7 (Fig. 2e), the (101) plane corresponds to molecular correlations within the 1D chains, whereas the (010) plane is related to the chain extension along the *b* axis, and the (001) plane is indicative of correlations between interdigitated layers. During melting at 505 K, slippage of the 1D chains induces rapid disorder within the interdigitated layers. Upon heating to 508 K, the strong hydrogen bonds become weakened, leading to the progressive disintegration of the chains and increasing disorder between the interdigitated layers. Upon heating to 510 K, the strong hydrogen bonds break, and thus intermolecular correlation peaks vanish, signaling the completion of the melting process in MOIC-7 (Fig. 2f).

The XRD results indicate that the melting of MOIC crystals begins with the slippage and distortion of strong hydrogen-bonded structures, followed by rapid disordering upon the breaking of the hydrogen bonds. At the beginning of the glass formation, the breakage of hydrogen bonds causes an increase in the free volume required for molecular mobility, thereby avoiding both the breaking of coordination bonds and ligand decomposition. The stacked hydrogen-bonded network structure in MOIC-4 tends to more readily undergo slippage and distortion compared to the interdigitated structure involving multiple *π-π* interactions in MOIC-7. This is why MOIC-4 has a lower *T*_m_ than MOIC-7, indicating that the bonding structure controls the melting point of MOICs.

### Structural analyses

**Figure 3.**
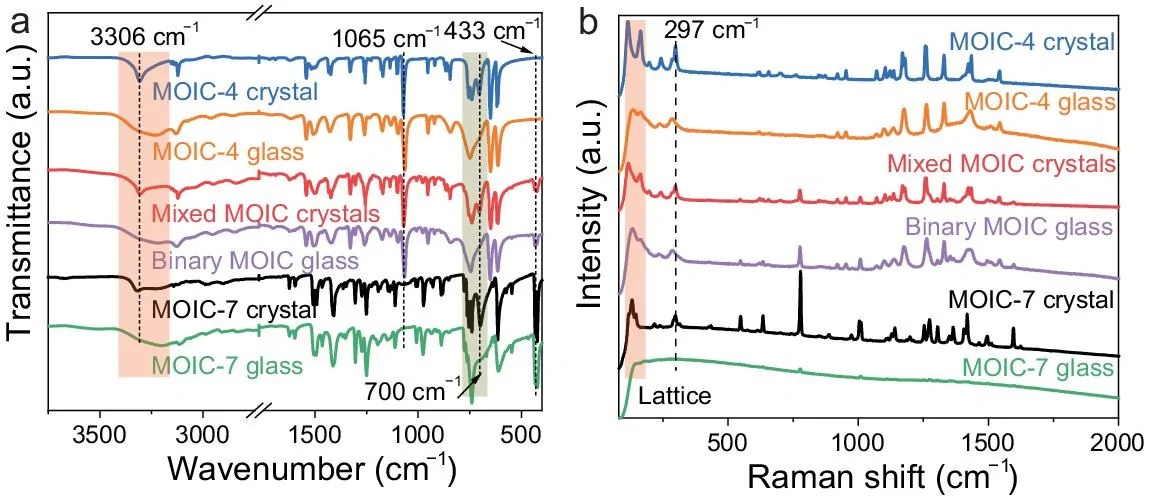
(a) Fourier transform infrared (FT-IR) spectra of MOIC crystals and glasses. (b) Raman spectra of MOIC crystals and glasses.

To understand the structure changes during vitrification, we analyze the FT-IR and Raman spectra of MOIC crystals and glasses (Fig. 3). Here, we choose ZnCl_2_(HIm_0.123_HbIm_0.877_)_2_ mixed crystals as a representative sample of the binary system. In FT-IR spectra (Fig. 3a), the peaks at 1065 cm^−1^ are seen, assigned to the C-H in-plane bending mode of HIm for both MOIC-4 crystal and glass [29]. The peaks at 433 and 422 cm^−1^ are ascribed to the in-plane ring bend mode of HbIm in MOIC-7 crystal and glass [30]. Both mixed crystals and their glassy counterpart exhibit the FT-IR signals of HIm and HbIm. The sharp peaks around 3306 and 700 cm^−1^ for MOIC crystals are associated with the stretching mode of N-H bonds from N-H…Cl and bending mode of C-H bonds from C-H…Cl, respectively [20,31]. The peaks of N-H bonds in all the samples become broadened and exhibit red-shift upon vitrification, while the peaks of the C-H bonds diminish. This implies that glass formation weakens the C-H…Cl hydrogen bonds while strengthening the N-H…Cl hydrogen bonds. The melting of MOIC crystals (Fig. 2) is accompanied by the breakage of N-H…Cl and C-H…Cl hydrogen bonds. During quenching, N-H…Cl hydrogen bonds are re-established, leading to the dominance of N-H…Cl hydrogen-bonded structural network. This is attributed to the higher bonding energy of N-H…Cl bonds compared with C-H…Cl bonds. Therefore, the disordered N-H…Cl hydrogen bond network is frozen in during the liquid-to-glass transition [20,32]. The Raman spectra (Fig. 3b) of all MOIC samples show a peak at 297 cm^−1^, which is attributed to the stretching modes of Zn-Cl, confirming the presence of the Cl ligand before and after vitrification [33]. To investigate the structural change during melting, we perform *in-situ* Raman spectroscopic measurements on the MOIC-4 crystal (Fig. S27). During melting, the peak at 283 cm^−1^ (stretching modes of Zn-Cl) becomes weaker and merges with the main peak at 297 cm^−1^, and thus a new broad peak at 293 cm^−1^ appears. This implies that melting causes the weakening of the Zn-Cl bonds, the breakage of C-H…Cl and N-H…Cl hydrogen bonds, the distortion of [ZnCl_2_(HIm)_2_] tetrahedra, and consequently structural disordering in different length scales [34]. Interestingly, MOIC-7 glass shows a stronger fluorescence background compared to other MOIC crystals and glasses because the former one has a higher probability for electron transfer between HbIm linkers.

**Figure 4.**
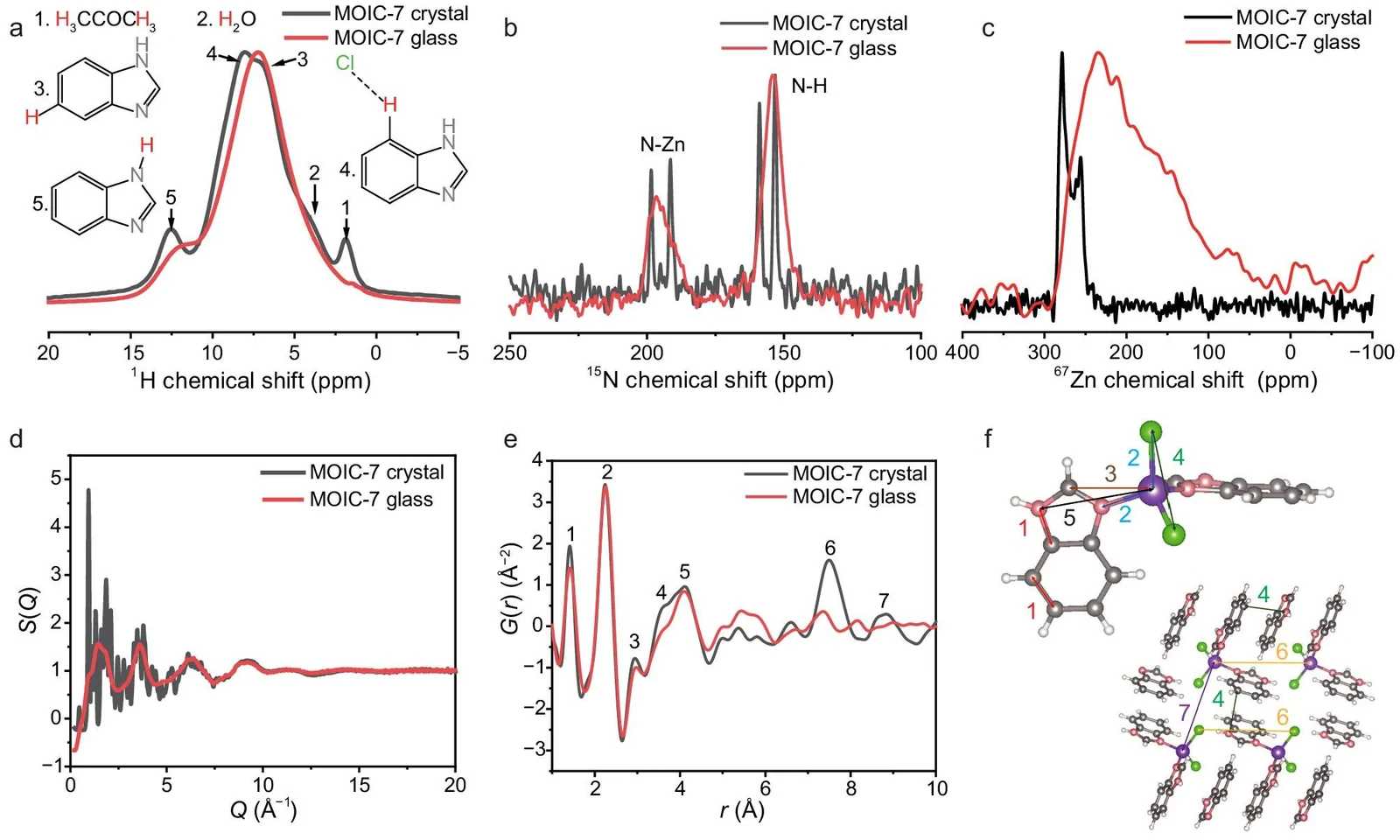
(a) The ^1^H solid-state magic-angle spinning (MAS) nuclear magnetic resonance (NMR) spectra of MOIC-7 crystal and glass. (b) The ^15^N cross-polarization MAS (CPMAS) NMR spectra of MOIC-7 crystal and glass. (c) The ^67^Zn CPMAS NMR spectra of MOIC-7 crystal and glass. (d) Faber-Ziman structure factor *S*(*Q*) of MOIC-7 crystal and glass. (e) Reduced pair correlation functions *G*(*r*) of MOIC-7 crystal and glass. (f) Schematic diagram of the atomic relationships in MOIC-7 structure. Note: purple spheres represent Zn, gray spheres represent C, green spheres represent Cl, white spheres represent H, and pink spheres represent N.

The short-range structural evolution trends in MOIC-7 crystals and glasses are observed using ^1^H, ^13^C, ^15^N, and ^67^Zn magic angle spinning NMR methods. Figure 4a–c shows the ^1^H, ^15^N, and ^67^Zn NMR spectra of MOIC-7 crystal and glass, respectively. In the ^1^H NMR spectrum (Fig. 4a), MOIC-7 displays the partial overlap of two main resonance peaks at about 6.7 and 8.5 ppm and three weak ones at 1.8, 4.4, and 12.6 ppm. In contrast, MOIC-7 glass shows a strong overlap of two main resonance peaks at 7.2 and 7.3 ppm and one weak peak at 12.4 ppm, implying a uniform structural environment of hydrogen atoms in the glass state. The ^1^H NMR spectrum of the MOIC-7 crystal is deconvoluted into five resonance peaks (Fig. S28, Table S3). However, the ^1^H NMR peaks at 1.8 and 4.4 ppm vanish when MOIC-7 is melt-quenched to glass state (Fig. S29, Table S4). Specifically, the 1.8 ppm resonance is attributed to residual acetone trapped within the crystal lattice [35]. This agrees with the TG results in Fig. 1e, where the desolvation of trapped acetone is observed. The 4.4 ppm signal, which is associated with H species stemming from the atmosphere [36], disappears after desolvation. The two main resonances at 8.5 ppm and 6.7 ppm arise from H atoms bonded to C atoms on the aromatic ring. The 8.5 ppm resonance is attributed to H atoms within the imidazole rings, whereas some of these H atoms are involved in the C-H…Cl hydrogen bonds, thus causing a decrease in the electron cloud density of the H atoms. When MOIC-7 crystal is melt-quenched to glass, the resonance peak shifts from 8.5 to 7.3 ppm (attributed to C-H bonds), indicating that the C-H…Cl hydrogen bonds are broken, whereas C-H bonds are intact. It is seen that the 12.6 ppm resonance peak, ascribed to H atoms bonded to N atoms, becomes weaker and broader as the crystal is vitrified [37]. This peak change is due to the replacement of the C-H…Cl hydrogen bonds with N-H…Cl hydrogen bonds. Such replacement results in the dominance of the hydrogen-bonded (N-H…Cl) structural network in MOIC glasses.

Figure S30 shows the ^13^C NMR spectra of MOIC-7 crystal and glass, where it is observed that upon vitrification, their ^13^C NMR peaks only slightly move to a lower chemical shift. This indicates that the de-shielding effect around the C atoms in the MOIC-7 crystal becomes weaker, i.e., vitrification can increase the electron density around C. The ^15^N NMR spectra of MOIC-7 crystal and glass (Fig. 4b) exhibit peaks at 154 and 197 ppm, which are attributed to the N atoms bonded to H and the N atoms coordinated to Zn, respectively [37,38]. In the crystal, the signals appear as pairs at 153/159 and 191/199 ppm. The two pairs are associated with two types of HbIm ligands in the crystal: one type constituting stacked planar layers, whereas the other forms interdigitated layers. Upon vitrification, the two pairs become two broad singlets, suggesting a wide distribution of the Zn-N bond lengths and N-Zn-Cl (N) bond angles, i.e., a subtle short-range disorder. Interestingly, analogous to ZIF glasses, MOIC-7 glass exhibits a pronounced asymmetric peak and a low-frequency tail in the ^67^Zn spectrum (Fig. 4c). Since ^67^Zn is a spin −5/2 quadrupole nucleus, the second-order quadrupole effect is often the main cause of broadening, which is proportional to the square of the quadrupole coupling or electric-field gradient. The drastic increase in width to the right indicates a larger electric-field gradient for glasses at the ^67^Zn site due to distortions from the local bonding environment and/or charge distribution. This further confirms the short-range structural disorder in MOIC glasses, implying the freezing of multiple conformations of tetrahedral units [34]. In the MOIC melt structure, the steric hindrance increased by HbIm lowers the ability of molecules to return to their equilibrium positions, i.e., to the short-range order structure.

The high-energy synchrotron XRD technique is employed to probe the short-, medium-, and long-range structural evolution of MOIC-7 crystal upon vitrification. The acquired diffraction images collected by synchrotron XRD are processed using the self-made Igor program [39]. Figure 4d shows the Faber-Ziman structure factor *S*(*Q*) curve of both MOIC-7 crystal and glass, where sharp diffraction peaks and broad peaks are observed in the crystal and the glass, respectively. This further confirms that the long-range order is lost upon vitrification [40]. The reduced pair correlation functions *G*(*r*) curves in Fig. 4e show seven main atom-atom correlation peaks in MOIC-7 crystal and five main peaks in MOIC-7 glass. The atom-atom correlations are schematically displayed in Fig. 4f [41–44]. Peak 1 at 1.4 Å corresponds to C-C(N) interaction in the HbIm rings. Peak 2 at 2.2 Å is ascribed to the overlap of the correlations of both the nearest-neighbor Zn-N (2.0 Å) and Zn-Cl (2.2 Å), implying that the metal-ligand bonding remains intact in MOIC-7 glass. Peak 3 at 2.95 Å and peak 5 at 4.1 Å are attributed to Zn-C and Zn-N (next-neighbored N) correlations, respectively. The intramolecular correlations (C-C/N), Zn-N, and Zn-Cl remain in MOIC-7 glass. Peak 4 at 3.6 Å is assigned to both the nearest-neighbored Cl-Cl correlation and the aromatic ring-ring correlations. The decrease in peak 4 intensity upon vitrification implies that the *π-π* stacking is disrupted. Peak 6 at 7.5 Å corresponds to intermolecular nearest-neighbored Zn-Zn and Cl-Cl correlations, while peak 7 at 8.8 Å is ascribed to the next-neighbored Zn-Zn correlations. In contrast to the preserved intramolecular correlations, the intermolecular correlations (Zn-Zn and Cl-Cl) disappear in MOIC-7 crystal upon vitrification, suggesting that the hydrogen-bonded network becomes disordered in the medium-range. The uncertainty of the derived position of the atom-atom correlation peak in both MOIC-7 crystal and glass is evaluated by deconvoluting *T*(*r*) curves. The result shows that the uncertainty is about 1% (Tables S5 and S6). This further indicates that the conformational disorder indeed exists in MOIC-7 glass.

### Extension of the ligand-tuning approach

The ligand-tuning approach is further extended through multi-level structural tuning to synthesize four MX_2_L_2_ crystals, where M is the metal node (Co or Cu), X is the hydrogen bond acceptor (Cl or SCN), and L is the hydrogen bond donor (HIm or HbIm). From the XRD patterns in Figs S31–S34, we can identify four kinds of MOIC crystals, which possess different crystal structures, consistent with the literature [13,45–47]. From DSC upscan curves in Figs S35–S38, we obtain the *T*_m_ values of MOIC crystals and the *T*_g_ values of their glass states (Table S7). Specifically, ZnCl_2_(HbIm)_2_ and CoCl_2_(HbIm)_2_, both of which have the interdigitated structure, exhibit the *T*_m_ values of 508 and 512 K, respectively. In contrast, Co(SCN)_2_(HbIm)_2_ and Cu(SCN)_2_(HIm)_2_, composed of only stacked 1D hydrogen-bonded chains, show the *T*_m_ values of 464 and 446 K, respectively. The derived Co-based MOIC glass exhibits an increase in *T*_g_ as HIm is substituted by HbIm (Figs S35 and S36), due to the enhanced steric hindrance caused by HbIm [10]. Substitution of Cl^−^ with the larger SCN^−^ ligand in MOIC structure avoids interdigitation between two-dimensional layers, thereby lowering the *T*_m_ of MOIC crystals (Fig. S37). The substitution of one type of metal node (e.g., Co) by another (e.g., Cu) leads to the formation of a square-planar Cu(SCN)_2_(HIm)_2_ complex, whose geometry is unfavorable for the formation of a tetrahedral network. Therefore, the Cu-based MOIC glass exhibits a lower *T*_g_ (Fig. S38) than the Co-based one. These results demonstrate the potential of compositional design of MOIC crystals to modulate their coordination network, and thereby improve the physical properties of their glass state.

### Optical properties of MOIC-7 crystal and glass

**Figure 5.**
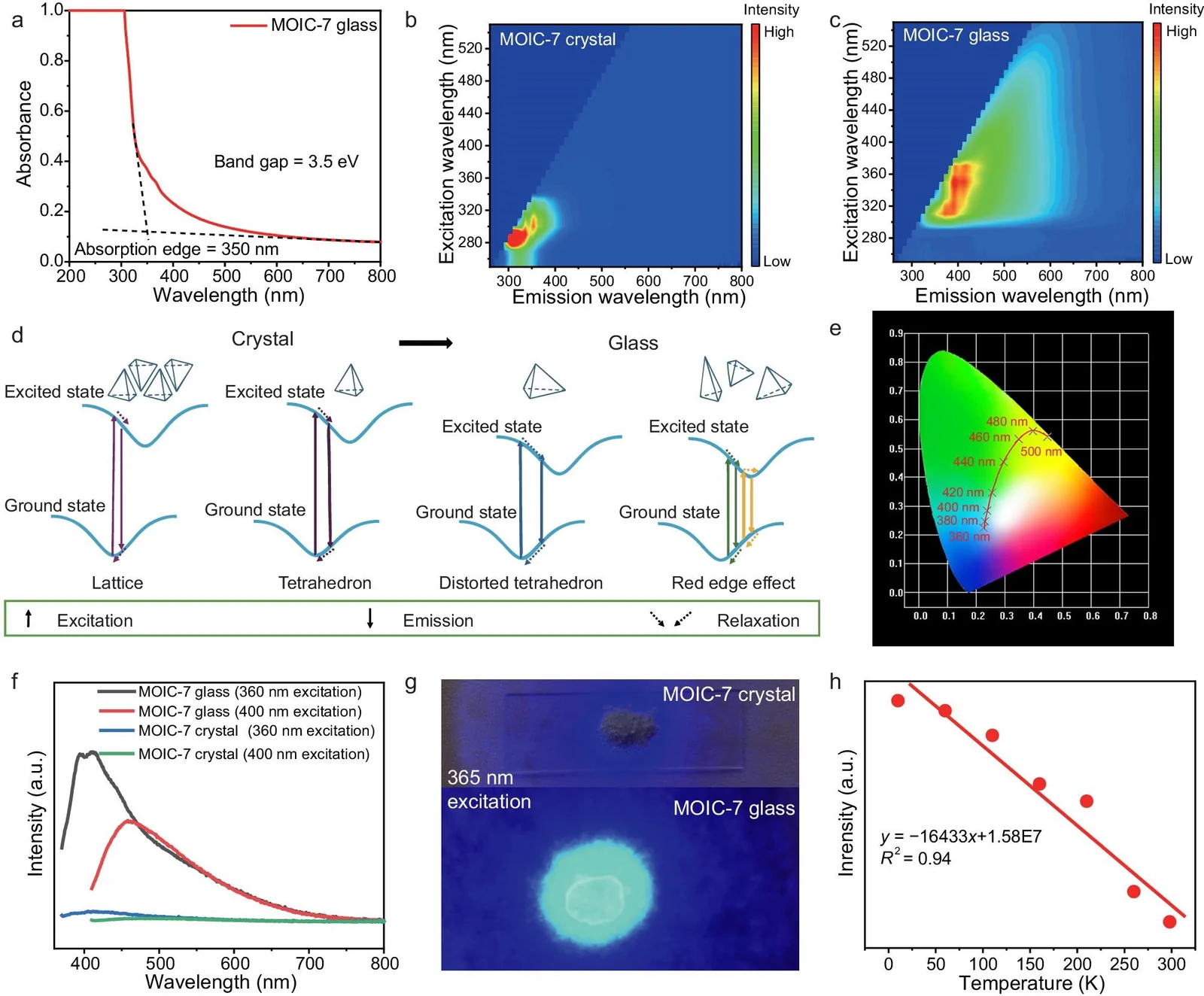
(a) Absorption spectrum of MOIC-7 glass. (b, c) Two-dimensional fluorescence (excitation/emission) spectra of MOIC-7 crystal (b) and MOIC-7 glass (c). (d) Schematic diagram of the luminescence mechanism. (e) International Commission on Illumination (CIE) chromaticity coordinates of the emission spectra of MOIC-7 glass. (f) Fluorescence spectra of MOIC-7 crystal and glass under 360 nm and 400 nm excitations. (g) Photos of MOIC-7 crystal and glass under excitation of a 365 nm laser. (h) The temperature dependence of the photoluminescence intensity of MOIC-7 glass under 360 nm excitation.

Inspired by the observation of strong fluorescence in MOIC-7 glass (Fig. 3b), we explore the optical functions of MOIC-7 crystal and glass. The optical absorption spectrum of MOIC-7 glass in Fig. 5a shows an absorption edge at 350 nm, below which the absorbance sharply increases with decreasing wavelength. The absorption signal is ascribed to the *π* electron transition from the valence band to the conduction band of the HbIm. From the absorption edge, the band gap of the ligands is estimated to be 3.5 eV, which is consistent with the value calculated using the Periodic Density Functional Theory [48].

Figure 5b and c shows the two-dimensional (excitation/emission) fluorescence spectra of MOIC-7 crystal and glass, respectively, which are constituted by combining the excitation and PL emission spectra shown in Figs S39 and S40. A narrow emission peak is seen at 308 nm in the crystal under excitation in the wavelength range of 250–290 nm. Three emission peaks are observed at 336, 353, and 371 nm in the crystal under excitation in the wavelength range of 300–330 nm. MOIC-7 glass shows three broad emission peaks at 373, 392, and 412 nm under excitation in 300–370 nm and a broad emission peak in the visible range under excitation in 370–550 nm. Interestingly, the broad emission peaks of MOIC-7 glass under laser excitation in the wavelength range of 370–550 nm shift to longer wavelength with increasing the excitation wavelength, i.e., a red shift occurs. The structural origin of the observed photoluminescence (PL) can be explained as follows (Fig. 5d). First, the narrow emission peak at 308 nm corresponds to the periodically arranged HbIm, where the rigidity of the crystal suppresses the non-radiative transition. Second, the three emission peaks both in the crystal and glass are attributed to the π-π* electron transition of HbIm [49]. The broadening and red shift of the PL peaks of the crystal are ascribed to the distortion of the medium- and long-range structure. Third, the excitation-dependent PL of the glass might be associated with the red-edge effect [50]. The red-edge effect is commonly observed in solute-solvent systems [51,52] but not in single-component solid materials [53].

The excitation-dependent PL color points of MOIC-7 glass are marked in the International Commission on Illumination chromaticity coordinates (Fig. 5e), where the PL color varies from blue to yellow with increasing the excitation wavelength. This implies that the excitation-dependent PL is associated with the red-edge effect arising from slow electron transition from high to low energy levels in MOIC glasses. It should be mentioned that lasers of different wavelengths can excite the electrons within molecules to different energy states, leading to a broad spectrum of PL. However, the disordered hydrogen-bonded structural network of MOIC glasses features slow electron transitions from high to low energy states during laser excitation, causing the PL at longer wavelengths, i.e., a red-shift PL [54]. Distinct conformers of ZnCl_2_(HbIm)_2_ molecules are frozen in the glass network, as evidenced by the vitrification-induced broadening of the ^67^Zn NMR signal of MOIC-7 crystals (Fig. 4c). In addition, the phosphorescence behavior is also observed in MOIC and ZIF-62 glasses [55–57].

In contrast to MOIC-7 crystal, its glass state exhibits significantly enhanced PL under excitation at 360 and 400 nm (Fig. 5f). Photographs of MOIC-7 crystal and glass under excitation at 365 nm are shown in Fig. 5g. It is seen that MOIC-7 glass can emit strong blue light. In addition, the internal PL quantum yield (PLQY) of MOIC-7 glass (3.4%) is around three times higher than that of MOIC-7 crystal (1.2%) under excitation at 370 nm (Table S8). By integrating the PL intensity over the wavelength, the integrated value of MOIC-7 glass linearly decreases with an increase in temperature due to the increased probability of non-radiative transition (Fig. 5h and Figs S41–S43). In summary, vitrifying MOIC-7 causes a redshift and enhanced PL in the visible light range, indicating its potential value in anti-counterfeiting applications.

## CONCLUSIONS

We designed and synthesized two types of hydrogen-bonded MOIC crystals, i.e., MOIC-4 and MOIC-7 crystals, with the composition of ZnCl_2_(HIm)_2_ and ZnCl_2_(HbIm)_2_, respectively. The hydrogen-bonded structural network was generated in the MOIC crystal by substituting the coordination bonds in ZIF-4 and -7 with hydrogen bonds facilitated by introducing Cl^−^ ligands. It was found that the structural differences between MOIC-4 and MOIC-7 caused distinct phase evolution trends during heating. The stacked structure in MOIC-4 makes the hydrogen bond chains prone to sliding and twisting, while the interlaced structure in MOIC-7 makes these chains stable and integrated. We transformed the two types of MOIC crystals into their glass states via melting-quenching, during which the hydrogen bonds become broken. The melting-quenching process causes the loss of the medium- and long-range intermolecular correlations. In contrast, the short-range correlations (e.g., Zn-N, Zn-Cl, and Zn-C correlations) remain in the glass, whereas the short-range structural order vanishes. Thus, the formation mechanism of MOIC glasses has been clarified.

Furthermore, we developed a binary eutectic MOIC system with varying molar ratio of HbIm/[HIm + HbIm] and its glassy counterpart by melt-quenching. The *T*_g_ of the glass increases with substituting HbIm for HIm because HbIm increases the steric hindrance of the structural network. In addition, we fabricated a series of MOIC crystals and glasses by varying the type of metal nodes (such as Zn, Co, and Cu) and also by substituting acid anion ligand (SCN^−^) for Cl^−^.

We observed enhanced PL in the visible light region in MOIC-7 glass compared with its crystalline counterpart. In addition, the internal PLQY of MOIC-7 glass (3.4%) is nearly three times higher than that of MOIC-7 crystal (1.2%). The excitation-dependence of PL in the glass arises from different ways of the connections between node-ligand tetrahedra in the hydrogen-bonded structural network, i.e., from a variety of short-range disorder. The strong PL makes MOIC-7 glass a strong candidate for photonic applications.

## Supplementary Material

nwag349_Supplemental_File
