# Supplementary material for: Glass formation of metal organic–inorganic complexes
Source: Natl Sci Rev. 2026 Jun 8;13(16):nwag349. doi: 10.1093/nsr/nwag349 (PMC13520117; doi:10.1093/nsr/nwag349)
Supplement: Supplementary file 1 [file nwag349_supplemental_file.pdf]

Supporting Information for

# Glass Formation of Metal Organic-Inorganic Complexes

Tianzhao Xu[1], Zhencai Li[1*], Jia-Xin Wu[2], Zihao Wang[3], Hanmeng Zhang[4], Huotian Zhang[5], Lars R. Jensen[6], Kenji Shinozaki[7], Feng Gao[5], Haomiao Zhu[3], Ivan Hung[8], Zhehong Gan[8], Jinjun Ren[4], Zheng Yin[2], Ming-Hua Zeng[2*], Yuanzheng Yue[1*]

[1]Department of Chemistry and Bioscience, Aalborg University, Aalborg 9220, Denmark

[2]School of Chemistry and Pharmaceutical Sciences, State Key Laboratory for Chemistry and Molecular Engineering of Medicinal Resources, Guangxi Normal University, Guilin 541004, P. R. China

[3]Xiamen Research Center of Rare Earth Materials, Haixi Institutes, Chinese Academy of Sciences, Xiamen 361021, China

[4]Shanghai Institute of Optics and Fine Mechanics, Chinese Academy of Sciences, Shanghai 201800, China

[5]Department of Physics, Chemistry, and Biology (IFM), Linköping University, Linköping 583 30, Sweden

[6]Department of Materials and Production, Aalborg University, Aalborg 9220, Denmark

[7]Research Institute of Core Technology for Materials Innovation, National Institute of Advanced Industrial Science and Technology (AIST), Ikeda, Osaka 563-8577, Japan

[8]National High Magnetic Field Laboratory, Tallahassee FL 32310, USA

*Corresponding authors. E-mail addresses: zhli@bio.aau.dk; zmh@mailbox.gxnu.edu.cn; yy@bio.aau.dk

## Table of Contents

Materials and Methods ........................................................................................................ 3

Supporting Figures ........................................................................................................ 8

Supporting Tables ........................................................................................................ 48

References ........................................................................................................ 52

# Materials and Methods

## Materials

$ZnCl_2$ (Sigma, 98 %), $Cu(CH_3COO)_2{\cdot}H_2O$ (Merck, 99%), $CoCl_2$ (Sigma, 98 %), HIm (Imidazole, Sigma, 99.5 %), HbIm (Benzimidazole, Sigma, 98 %), KSCN (VWR Chemicals, 99 %), $NH_4SCN$ (Honeywell, 97.5%), ethanol (Absolute), and acetone (Absolute) were purchased and used for preparing the MOIC crystals.

## Preparation of the MOIC crystals and binary Zn-MOIC eutectic system

MOIC-4 and 7 were synthesized with the solvent-assist method: 10 mmol $ZnCl_2$ and 20 mmol HbIm were dissolved in 50 mL of acetone separately and stirred for 30 min. Then the HbIm solution was slowly added to the zinc chloride solution and the mixed solution was stirred for a further 2 h to obtain the clear precursor solution in which the molar ratio of Zn/HbIm was 1:2. The precursor solution was placed in a perforated aluminum foil covered bottle and heated in a 333 K water bath for 4 h, during which most of the solvent was removed. After the reaction, the bottle was taken out and left to dry further at room temperature. The white crystalline powder precipitated from the solution was collected and dried overnight in an oven under air to afford MOIC-7. The same process was used for MOIC-4 and the binary Zn-MOIC system, except that the organic ligands in the raw material were replaced with 20 mmol HIm (for MOIC-4) and the corresponding ratio of HIm and HbIm with a total amount of 20 mmol (for the binary Zn-MOIC system).

## Preparation of the M-MOIC crystals

**Co-MOICs**: $CoCl_2(HbIm)_2$ and $CoCl_2(HIm)_2$ were synthesized with the solvent-assist method analogous to that used for Zn-MOICs, with the only modification being the replacement of $ZnCl_2$ by 10 mmol $CoCl_2$. $Co(SCN)_2(HbIm)_2$ were synthesized by the solvent method [1]. 10 mmol $CoCl_2$, 20 mmol KSCN, and 20 mmol HbIm were separately dissolved in 40 mL of water, 50 mL of water, and 60 mL of ethanol, respectively. After stirring for 15 min to ensure complete dissolution, the KSCN and HbIm solutions were mixed and then added dropwise to the $CoCl_2$ solution, upon which an immediate precipitate formed. The mixture was stirred for 24 h, centrifuged, and washed twice with water. The resulting blue-violet powder was dried overnight at 343 K to obtain the final product.

**Cu-MOICs**: $Cu(SCN)_2(HIm)_2$ were synthesized by the solvent method [2]. 10 mmol of $Cu(CH_3COO)_2 \cdot H_2O$ and 20 mmol of HIm were dissolved separately in 200 mL and 100 mL of distilled water and stirred for 30 min. The HIm solution was added dropwise to the copper acetate solution, resulting in the immediate formation of a green precipitate. The mixture was stirred for an additional 2 h, after which the suspension was centrifuged and washed twice with water. The collected green powder was dried overnight at 343 K to yield the final product.

**Preparation of MOIC glasses**

MOIC glasses could be obtained with the melt-quenching method: 100 mg of MOIC-7 was ground into powder and placed on the surface of a commercial glass plate. The plate was then heated in an oven at 523 K, and the crystal melted into a transparent liquid after 5 minutes. The transparent MOIC-7 glass could be obtained after the plate was removed and the melt was quenched at room temperature. Other MOIC glasses were also prepared using the melt-quenching method, with the treatment temperatures set a little bit above their melting temperature, respectively.

**Structure Characterizations**

The room temperature PXRD was performed using an X-ray diffractometer (Empyrean XRD, Analytical) with a monochromator using Cu Kα radiation (1.5406 Å). The in-situ variable-temperature XRD for MOICs was recorded on a Panalytical X'Pert PRO diffractometer with Cu Kα radiation (λ=1.5418Å) and a variable-temperature system.

All the structures were optimized under PBE-D3(BJ)/DZVP-MOLOPT-SR-GTH level with default convergence criteria were used for geometry optimization, as well. The supramolecular interaction was studied by independent gradient model based on Hirshfeld partition (IGMH) [3] or interaction region indicator (IRI) [4] through Multiwfn [5,6]. The wavefunction files in molden format were generated by single-point computation of the geometry optimized experimental structure by Quickstep module [7] of the CP2K software package [8]. For the single-point calculation, the exchange-correlation energy was evaluated in the PBE approximation [9]. For Zn, valence electrons were described by triple-ζ valence with two polarization function molecular optimized (MOLOPT) basis sets optimized with PBE pseudopotentials, TZV2P-MOLOPT-PBE-GTH, and TZVP-MOLOPT-PBE-GTH for H, C,

N [10]. The dispersion interactions were treated at the DFT-D3(BJ) level [11,12]. For accuracy, a plane-wave cutoff for the electronic density to be 600 Ry and a relative cutoff of 50 Ry were used.

The TGA and DSC data were collected using a Netzsch STA 449 F3 instrument equipped with liquid $N_2$ cooling. All the testing conditions included a nitrogen atmosphere and a heating rate of 10 K/min.

The FTIR spectra of samples were obtained using a Bruker TENSOR II equipped with a Bruker Platinum attenuated total reflectance (ATR) attachment in the wavenumber range of 400–4000 $cm^{-1}$. The Raman spectroscopy data in the range of 80 to 2000 $cm^{-1}$ of the as-synthesized samples were obtained using a micro-Raman spectrometer (in Via, Renishaw). The wavelength of the laser was 785 nm.

Pair Distribution Function (PDF) analysis was performed using the data of high-energy synchrotron XRD (HEXRD) measurements. The data were collected on beamline BL04B2 at SPring-8 (Hyogo, Japan) with an X-ray energy of 113 keV (Si333). For the measurements, the sample was filled into 2mm capillaries made by silica glass. The acquired diffraction images collected by synchrotron XRD were processed using the self-made igor program [13]. The *Q*-intensity curve was obtained by converting the diffraction patterns collected by Synchrotron radiation XRD using the *FIT2D* software. The wave vector $Q = 4\pi\sin(2\theta)/\lambda$, where the scattering angle is $2\theta$, and the X-ray wavelength is $\lambda$. The data were further corrected for the effects of sample absorption, polarization, multiple scattering, Compton scattering, and fluorescence to obtain the normalized intensity I(*Q*). Finally, I(Q) was normalized by fitting the coherent atomic form factors $\langle f^2\rangle$ and $\langle f\rangle^2$ at high *Q* values to obtain the total structure factor S(*Q*) as follows:

$$S(Q) - 1 = \frac{\beta I(\mathrm{Q}) - (\langle f^2\rangle + I_m + I_c + I_f)}{\langle f\rangle^2} \quad (1)$$

Where $\beta$ is the normalization factor, and $I_m$, $I_c$ and $I_f$ are multiple scattering, Compton scattering, and fluorescence intensity. The obtained *S*(*Q*) was used to derive the reduced pair distribution function (PDF) *G*(*r*) in real space by inverse Fourier transformation with Lorch function *L*(*Q*) as,

$$G(r) = \frac{2}{\pi}\int_0^{16} Q(S(Q) - 1)\sin(Qr)\,L(Q)dQ \quad (2)$$

Solid-state cross-polarization magic angle spinning-nuclear magnetic resonance (CPMAS-NMR) experiments and $^{1}H$ MAS experiments were carried out using a Bruker Avance III HD 500 MHz NMR system. $^{13}C$-$^{1}H$ CPMAS experiments were performed with 3.3 μs 90° pulses and 3 ms contact time on the $^{1}H$ channel. The recycling delay was set to 8 s. The sample rotation frequency was 6.0 kHz with a 4 mm MAS-NMR probe. The $^{13}C$-$^{1}H$ CPMAS secondary calibration was adamantane, with the maximum intensity set to +38.5 ppm. $^{1}H$ MAS experiments were performed with 2.3 μs 90° pulses. The recycling delays were set to 8 s and 120 s, respectively, for the glass and crystal. The sample rotation frequency was 25.0 kHz in a 2.5 mm MAS-NMR probe. $^{1}H$ chemical shifts were referenced to a $H_2O$ secondary standard (δ=+4.8 ppm, δ=0 ppm relative to tetramethylsilane). The $^{15}N$ CPMAS and $^{67}Zn$ MAS NMR spectra were obtained at the National High Magnetic Field Laboratory (NHMFL) at 20.0 T (850 MHz $^{1}H$ frequency) using a Bruker Avance NEO spectrometer and a Low-E HX 3.2 mm MAS probe designed and manufactured by the NHMFL. The $^{15}N$ CPMAS NMR spectra of MOIC-7 crystal and glass were collected at 10 kHz MAS with 71.4 kHz $^{1}H$ decoupling *rf* field, a contact time of 10 ms, and an 1 s recycle delay. The $^{15}N$ chemical shift reference was second calibrated to $NH_3$. $^{67}Zn$ spectra were acquired using spin-echo with 4.5/9.0 μs pulses using an *rf* field of 18.5 kHz. under16 kHz MAS. A ~2 ms WURST pulse (16 kHz sweep, 18 kHz *rf*) was applied prior to the echo to enhance signal intensity.

**Optical performance**

The absorption spectra in the wavelength range of 200 to 800 nm of MOIC-7 glass were recorded using a UV-visible spectrophotometer (Cary Eclipse Fluorescence Spectrometer, Varian). Two-dimensional (excitation-emission) PL spectra from 260 to 800 nm were recorded on a fluorescence spectrophotometer (Edinburgh Instruments, FLS1000) equipped with a 450 W Xe lamp and detected with a PMT980 photomultiplier tube. The PL quantum yield (PLQY) was measured using an optical integrating sphere (N-M01, Edinburgh Instruments). When measuring the variable-temperature PL spectra, the sample temperature was controlled to vary from 10 to 298 K using an optical cryostat (DE202, Advanced Research Systems). PL decay curves were obtained using a fluorescence spectrophotometer (Edinburgh Instruments, FLS980) equipped with both continuous and microsecond pulsed 450 W Xe lamps and a Hamamatsu R2658P photomultiplier tube as detector. The lifetime can be fitted by the third-order exponential decay mode equation:

$$I = A_1 e^{(-t/\tau_1)} + A_2 e^{(-t/\tau_2)} + A_3 e^{(-t/\tau_3)} \qquad (3)$$

where $I$ is the PL intensity, $A_1$, $A_2$ and $A_3$ are the fitting parameters, $t$ is the time, $\tau_1$, $\tau_2$ and $\tau_3$ are the decay components. According to the value from the fitting of Eq. (3), the average lifetime $\tau$ can be calculated as:

$$\tau = (A_1\tau_1^2 + A_2\tau_2^2 + A_3\tau_3^2)/(A_1\tau_1 + A_2\tau_2 + A_3\tau_3) \qquad (4)$$

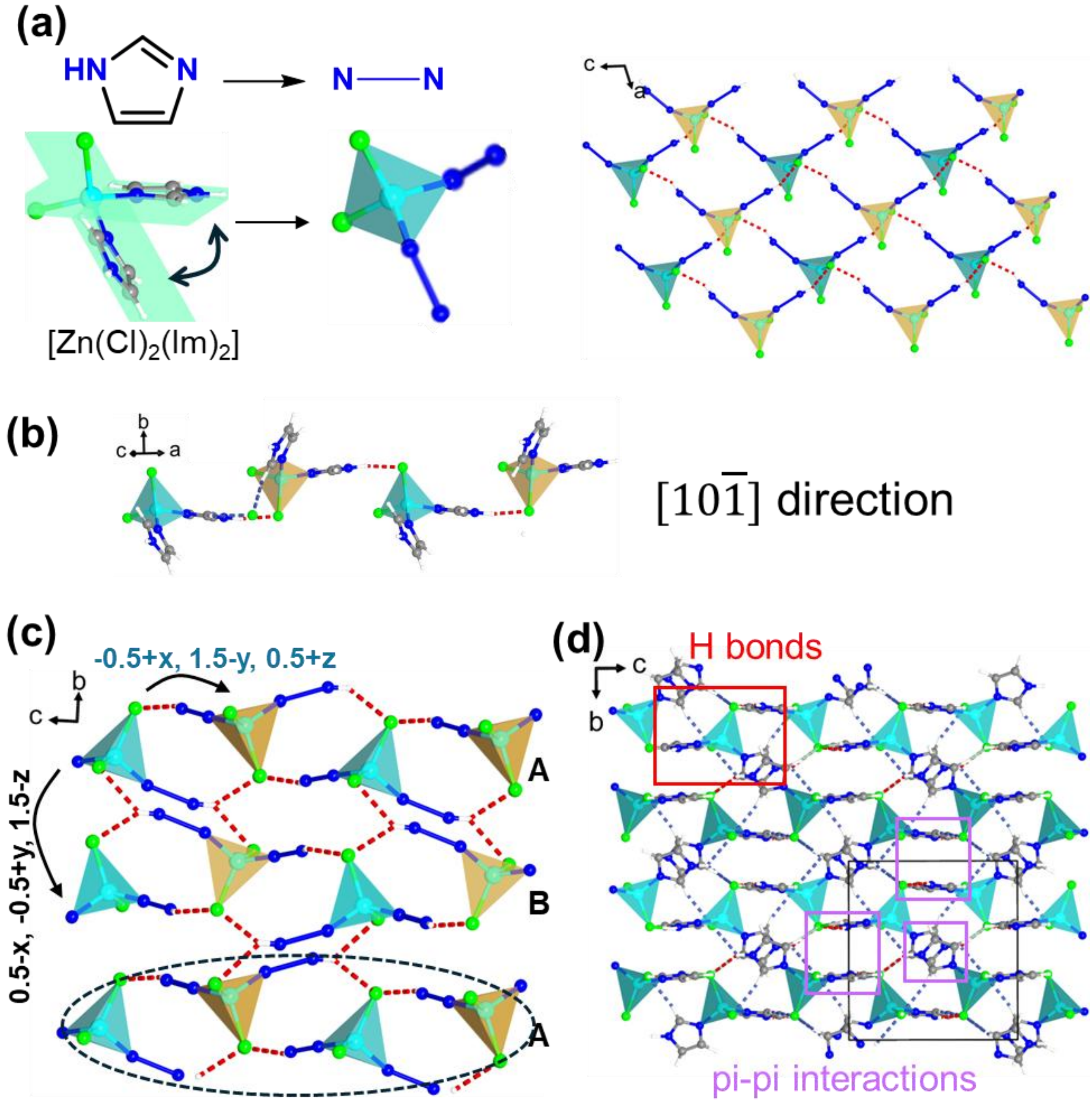


**Figure S1.** Structural schematic of the MOIC-4 crystal. **(a)** Simplified MOIC-4 molecule and the two-dimensional hydrogen-bonded layer. **(b)** Zigzag chain linked by the strongest N-H···Cl hydrogen bonds within the hydrogen-bonded layer. **(c)** ABAB stacking of the hydrogen-bonded layers. **(d)** Hydrogen bonds and π–π interactions in the crystal.

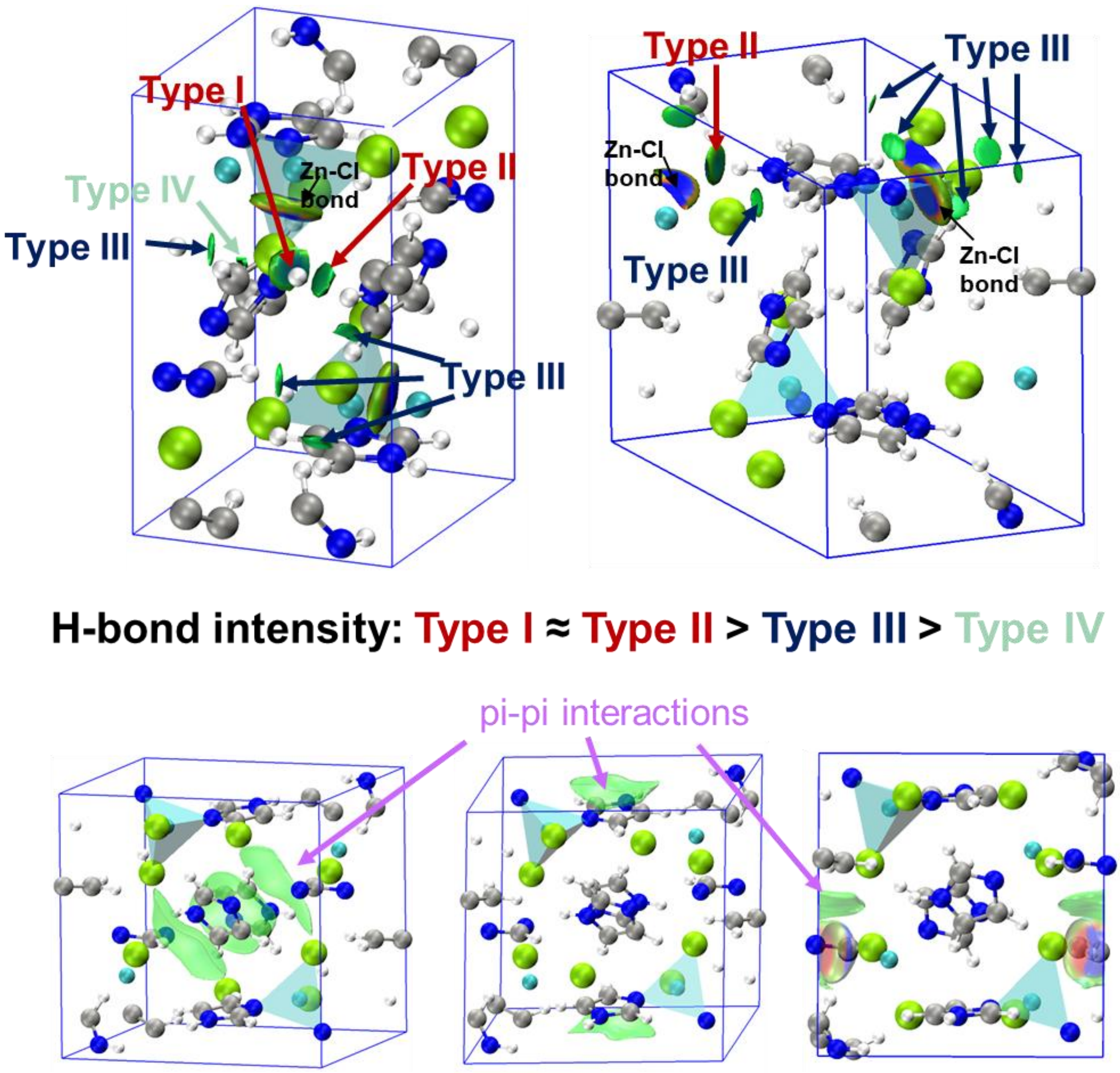


**Figure S2.** IGMH maps of MOIC-4 of selected Cl, H atoms for H-bonds/C, H atoms for π-π intercation. *sign(λ₂)ρ* was mapped on the IGMH isosurfaces of 0.01 a.u. according to the color bar lower and upper limits set to −0.05 and 0.05 a.u., respectively. In each $Zn_4C_{24}H_{32}N_{16}Cl_8$ unit cell, 14 hydrogen bonds of three strength types and three kinds of π-π interactions are identified.

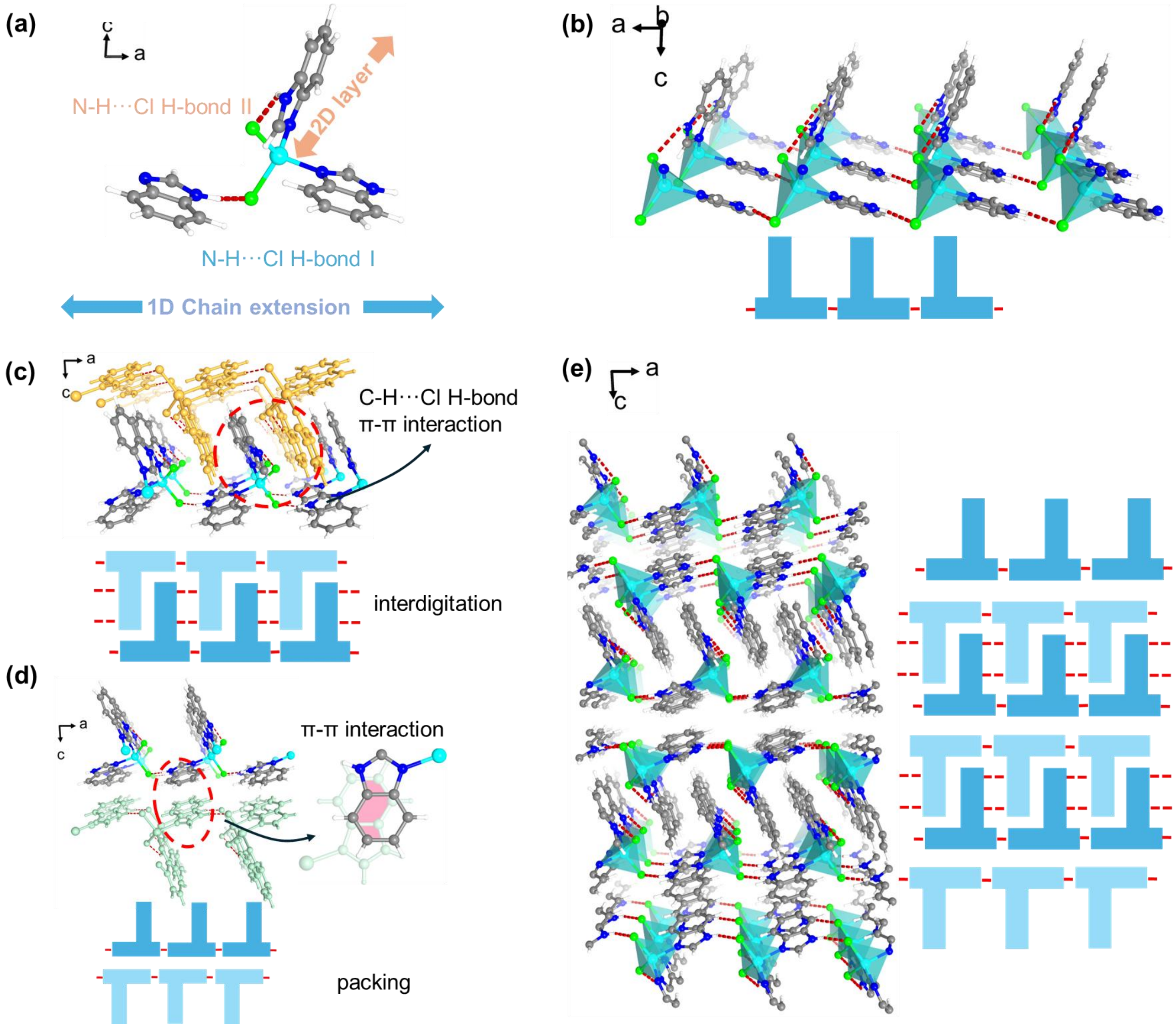


**Figure S3.** Structural schematic of the MOIC-7 crystal. **(a)** Two type of N-H···Cl hydrogen bonds between MOIC-7 molecules. **(b)** 2D hydrogen-bonded layers within MOIC-7. **(c)** Interdigitated arrangement of the hydrogen-bonded layers. **(d)** Stacking of the hydrogen-bonded layers. **(d)** Alternation of the two connection modes forming the final crystal structure.

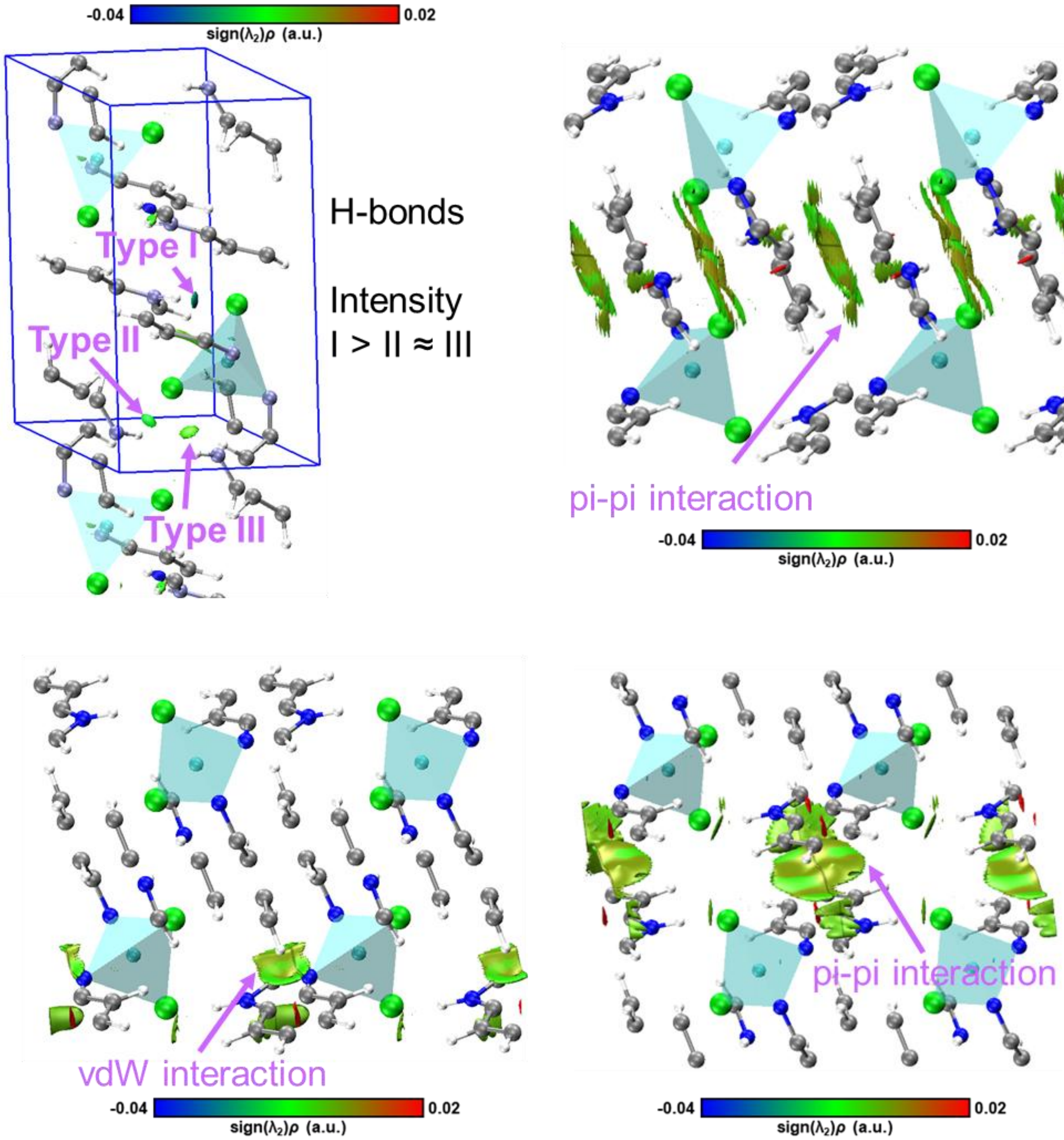


**Figure S4.** IRI maps of MOIC-7 of selected Cl, H atoms for H-bonds/C, H atoms for π-π intercation. *sign(λ2)ρ* was mapped on the IRI isosurfaces of 1 a.u. according to the color bar lower and upper limits set to −0.04 and 0.02 a.u., respectively.

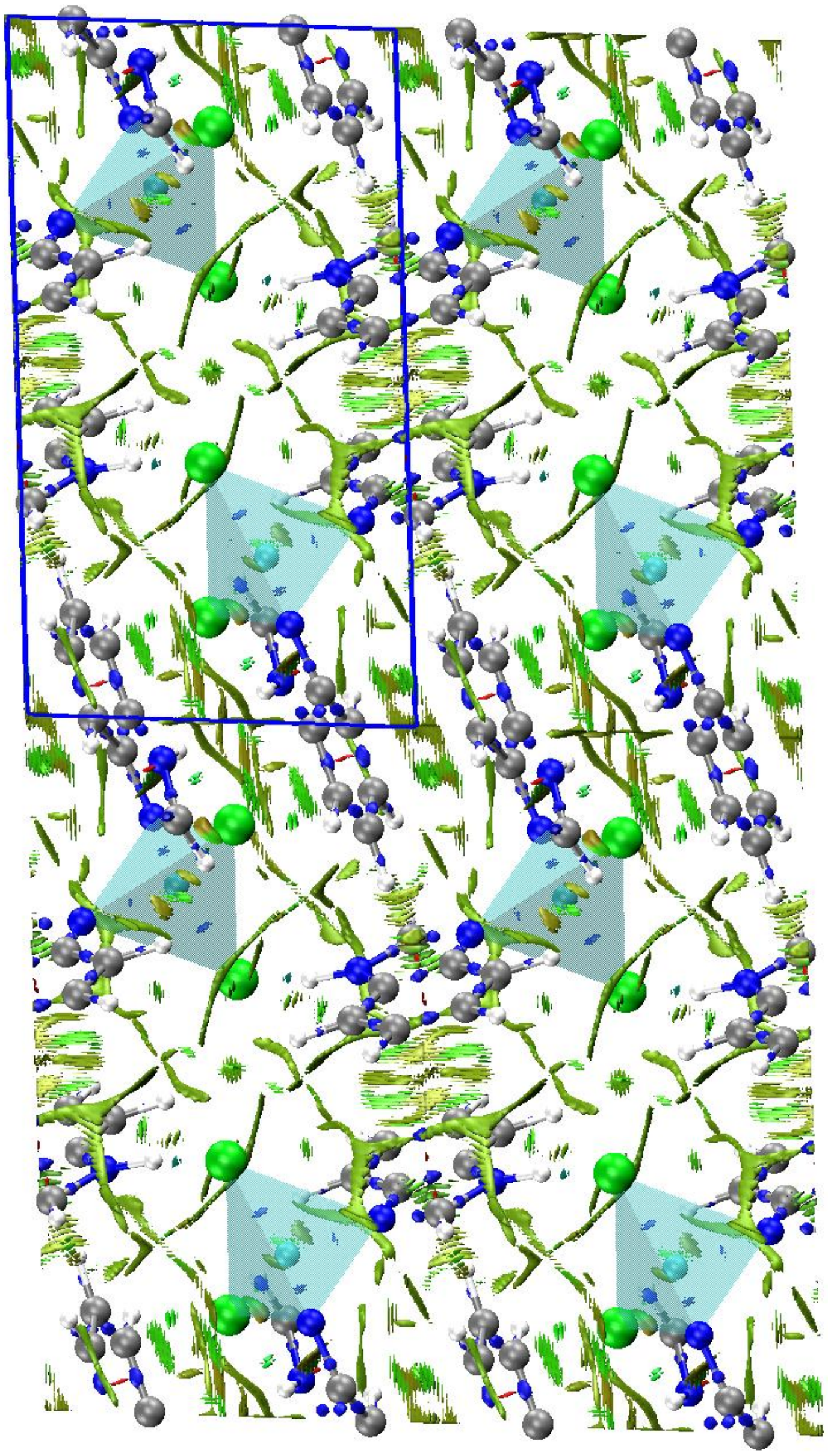

**Figure S5.** The overall IRI map of MOIC-7.

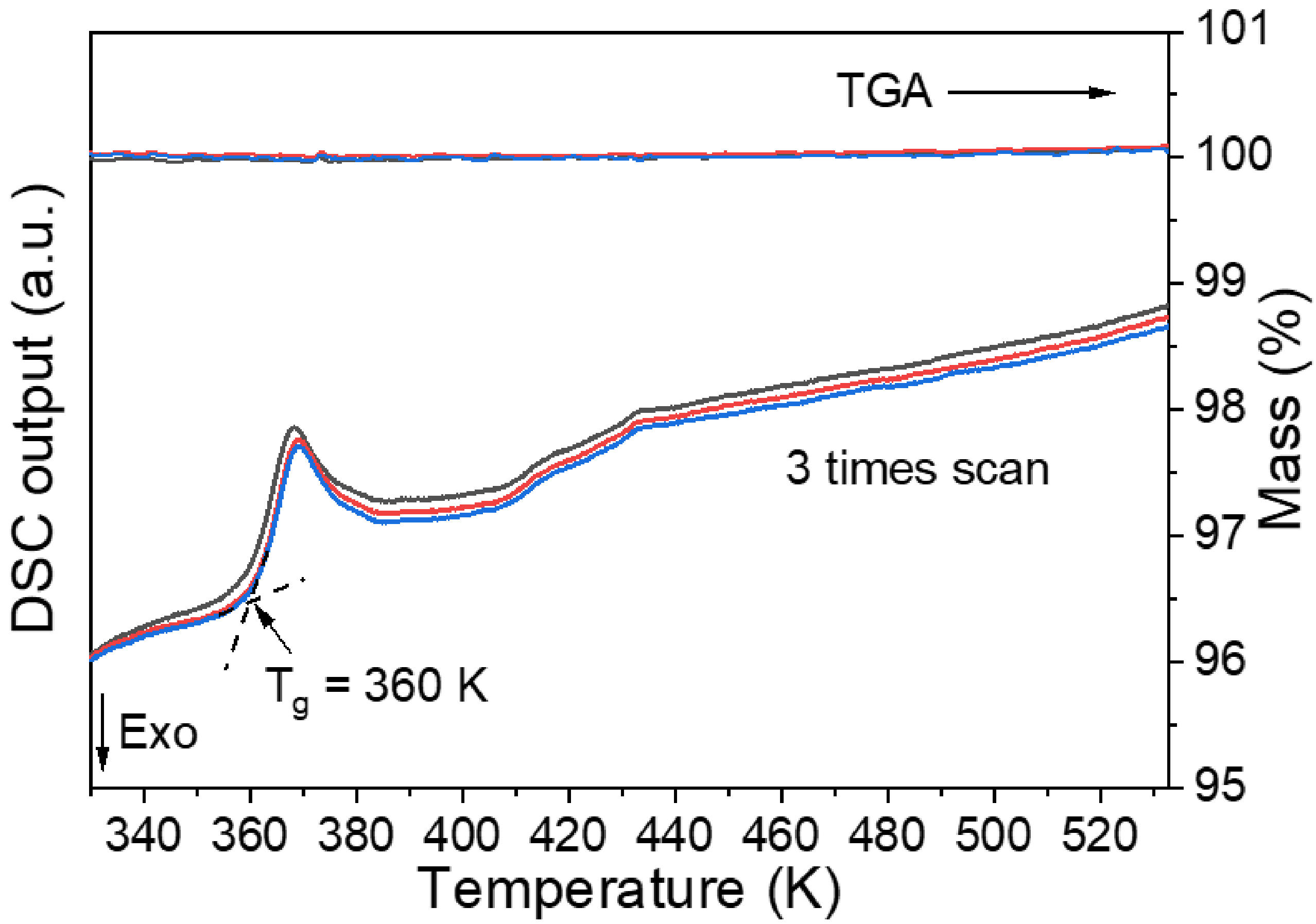


**Figure S6.** DSC-TGA curves of MOIC-7 glass obtained by three consecutive scans at a heating rate of 10 K/min in $N_2$ atmosphere to 523 K.

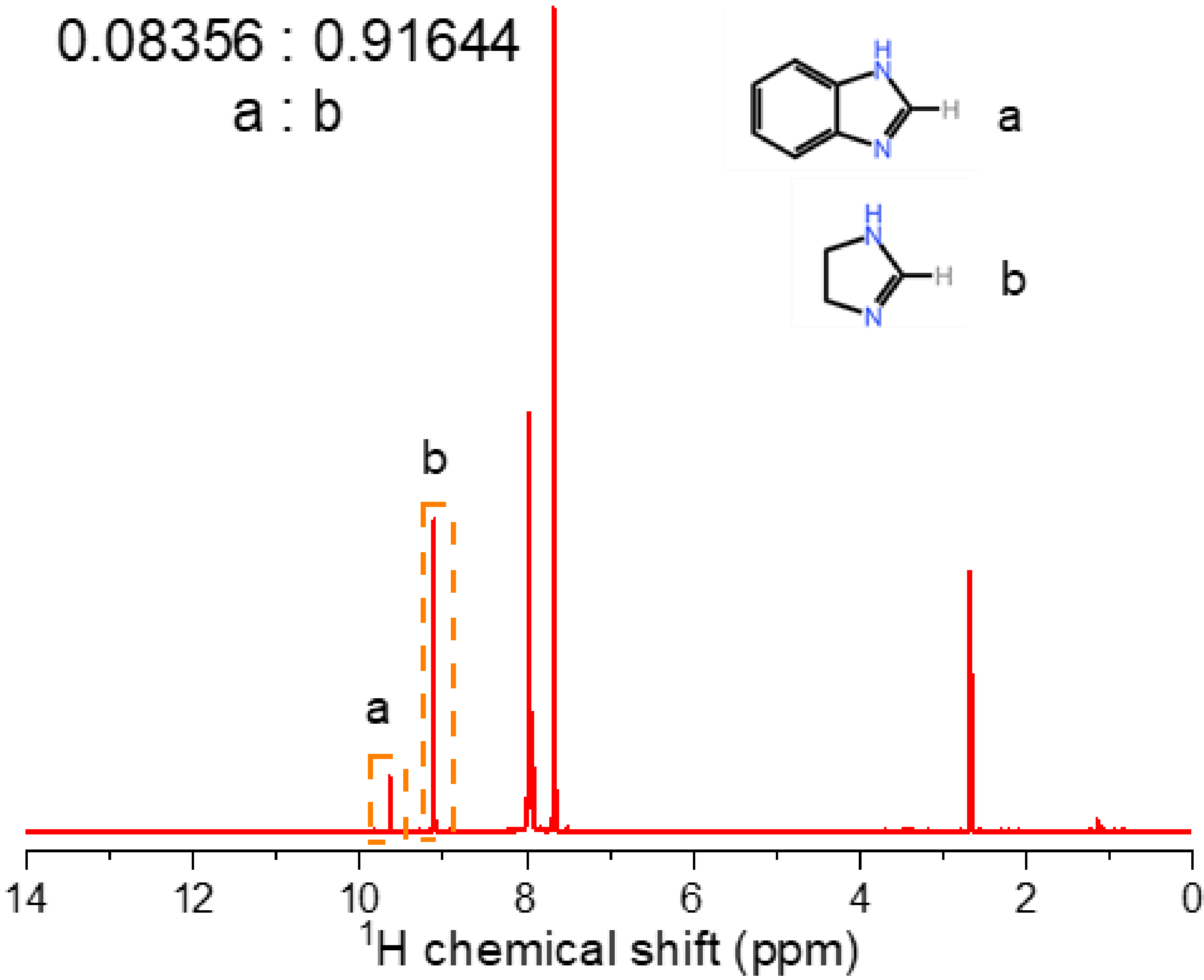


**Figure S7.** Solution $^{1}$H NMR spectra of binary system sample (ligand composition of precursor: HIm/HbIm = 0.917/0.083).

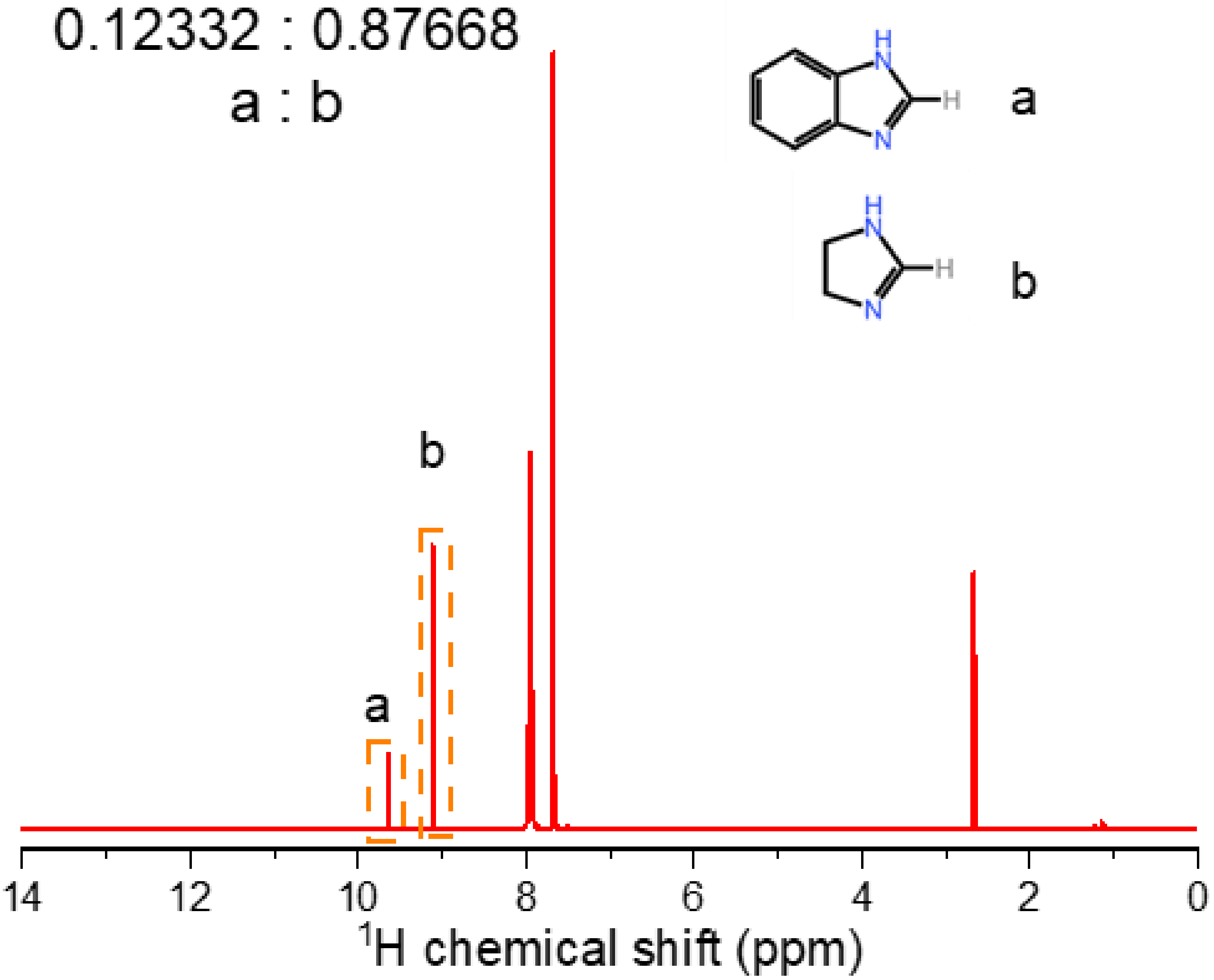


**Figure S8.** Solution $^{1}H$ NMR spectra of binary system sample (ligand composition of precursor: HIm/HbIm = 0.875/0.125).

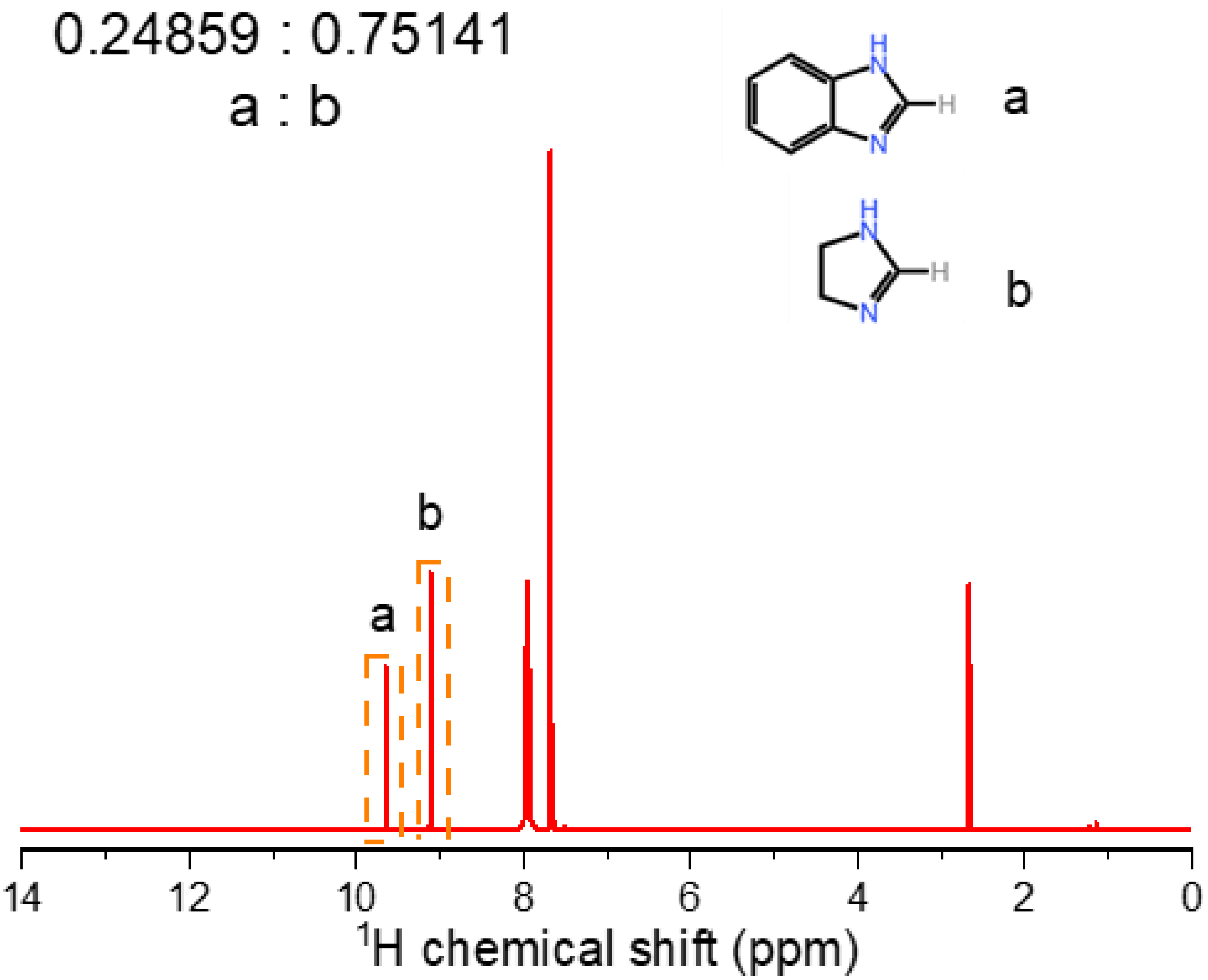


**Figure S9.** Solution $^{1}$H NMR spectra of binary system sample (ligand composition of precursor: HIm/HbIm = 0.75/0.25).

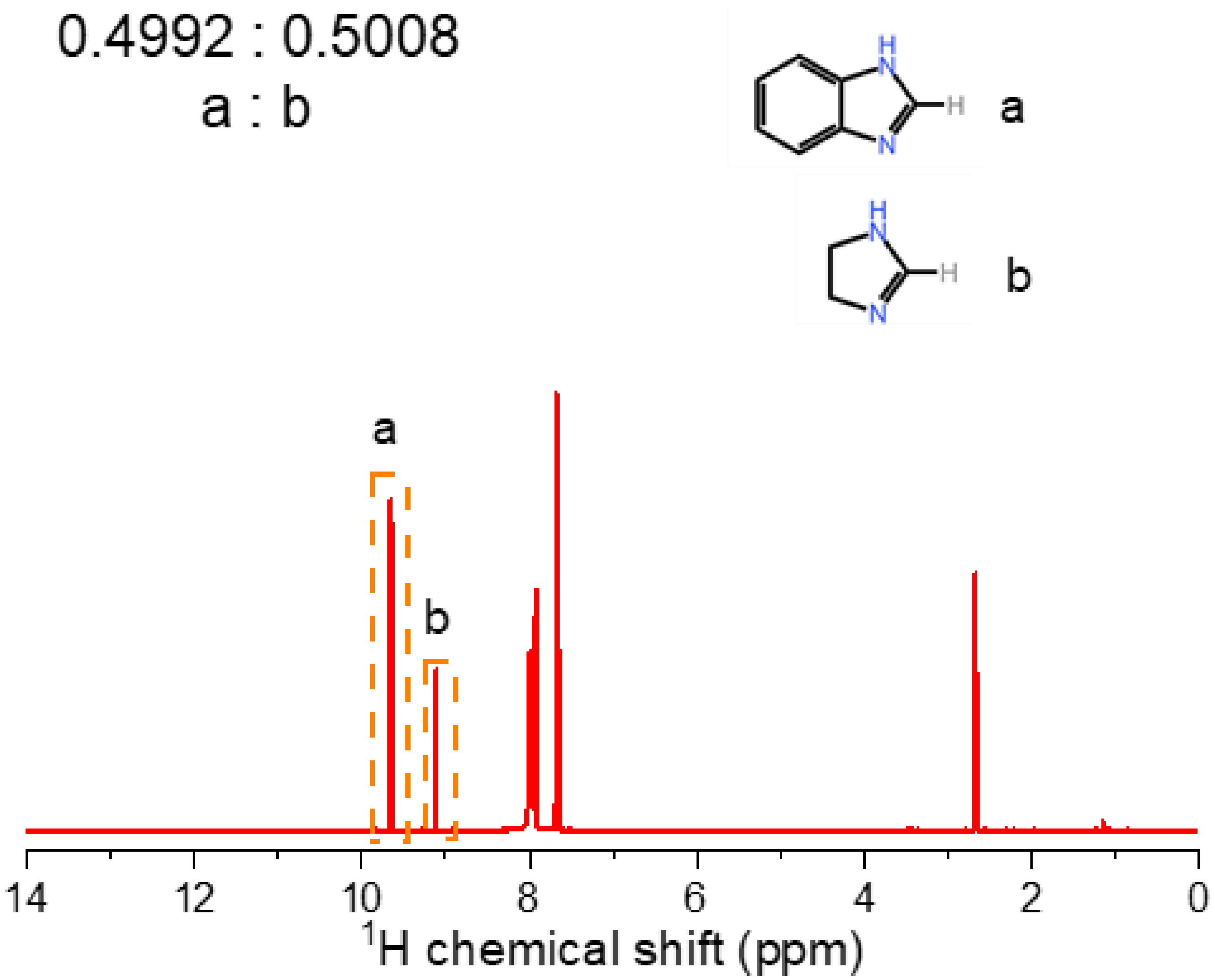


**Figure S10.** Solution $^{1}$H NMR spectra of binary system sample (ligand composition of precursor: HIm/HbIm = 0.5/0.5).

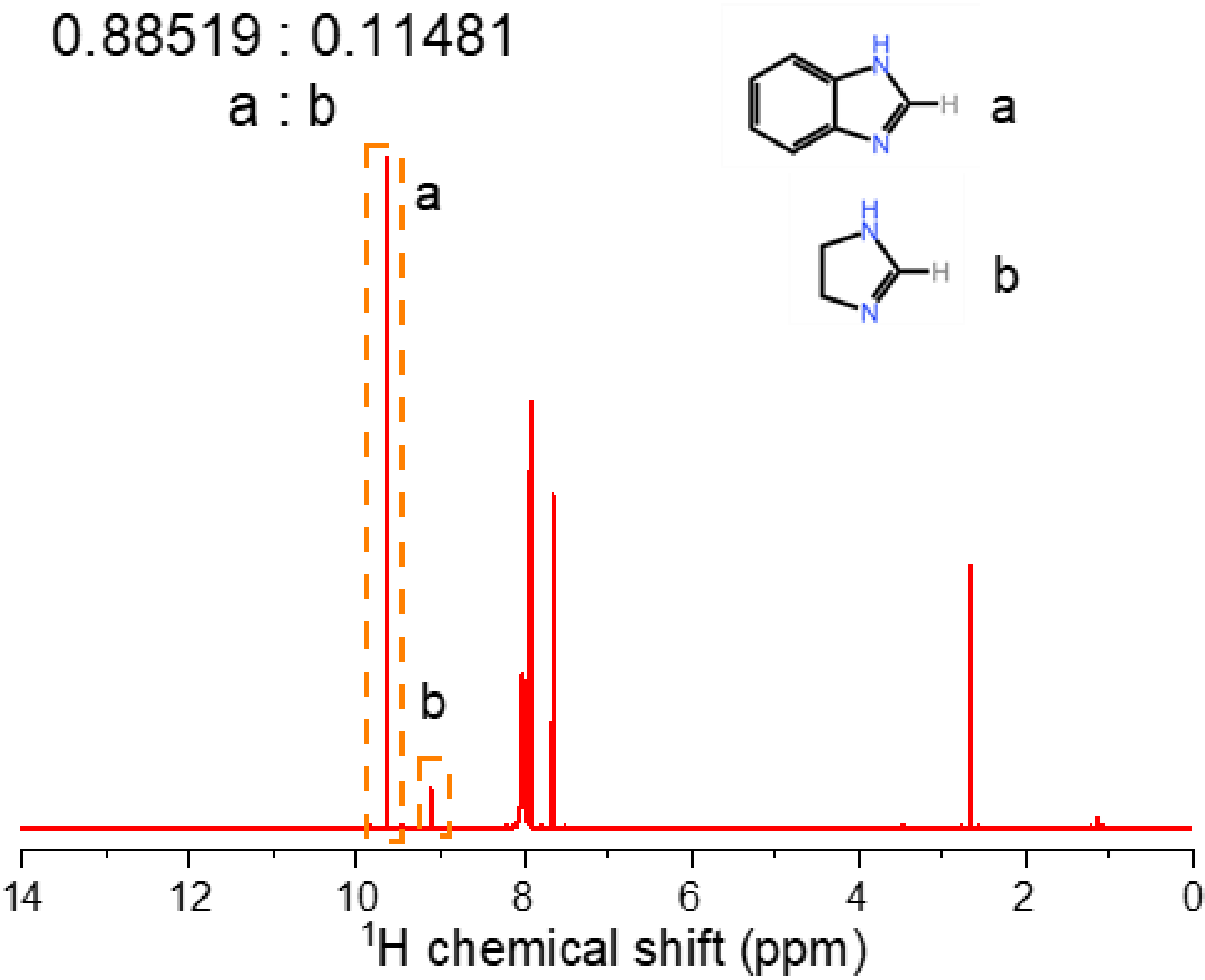


**Figure S11.** Solution $^1$H NMR spectra of binary system sample (ligand composition of precursor: HIm/HbIm = 0.125/0.875).

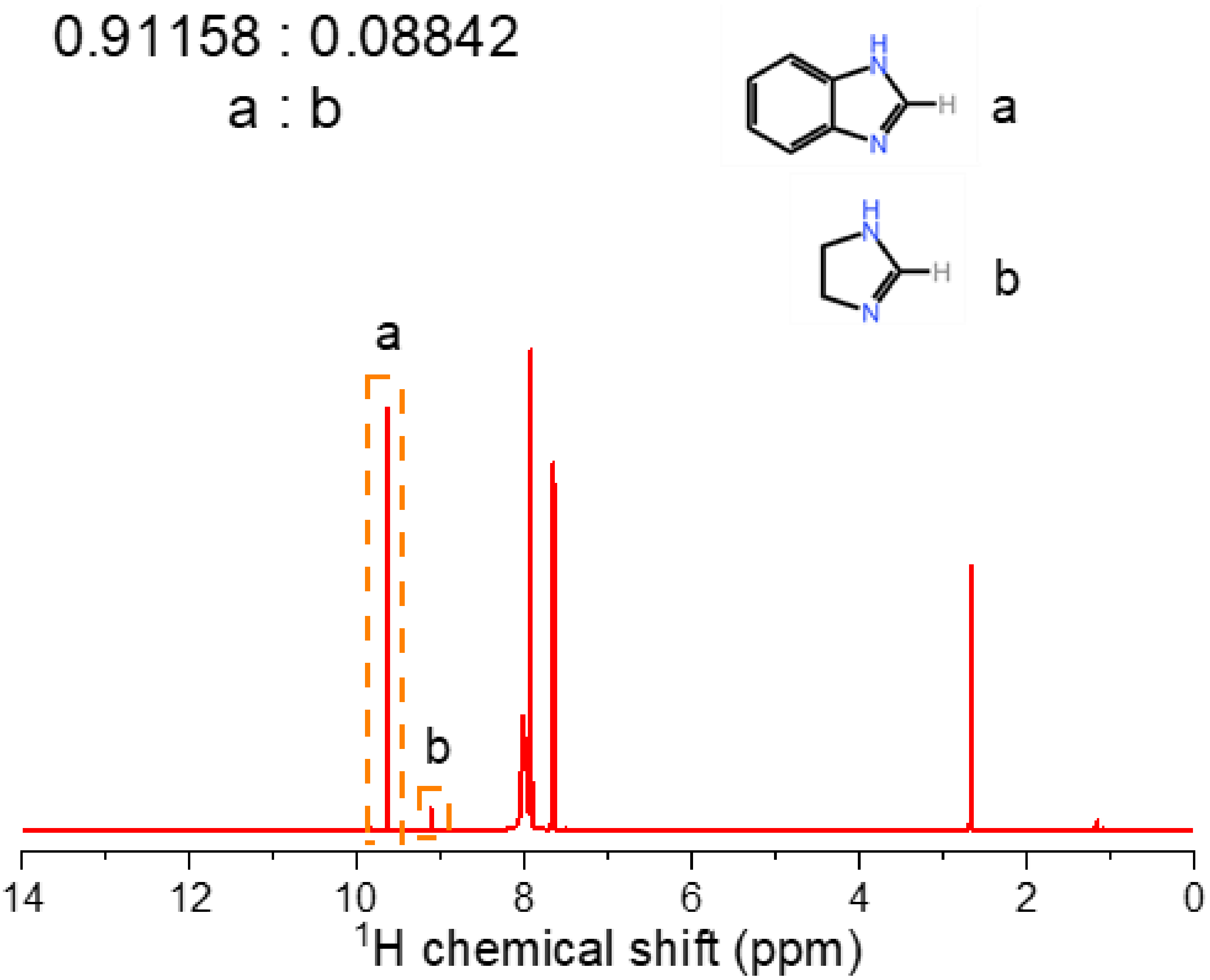


**Figure S12.** Solution $^{1}H$ NMR spectra of binary system sample (ligand composition of precursor: HIm/HbIm = 0.083/0.917).

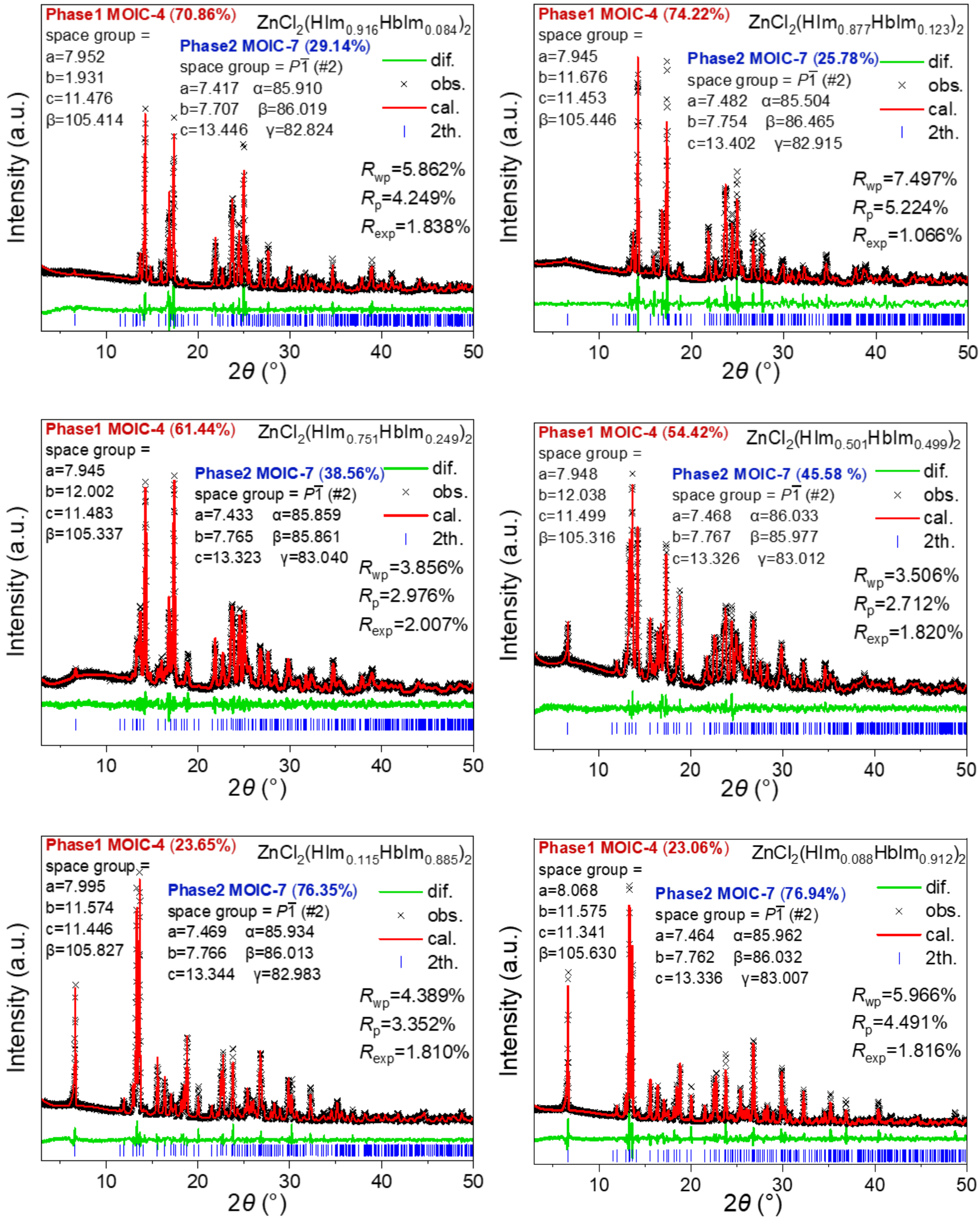


**Figure S13.** XRD refinement result of the binary MOIC system.

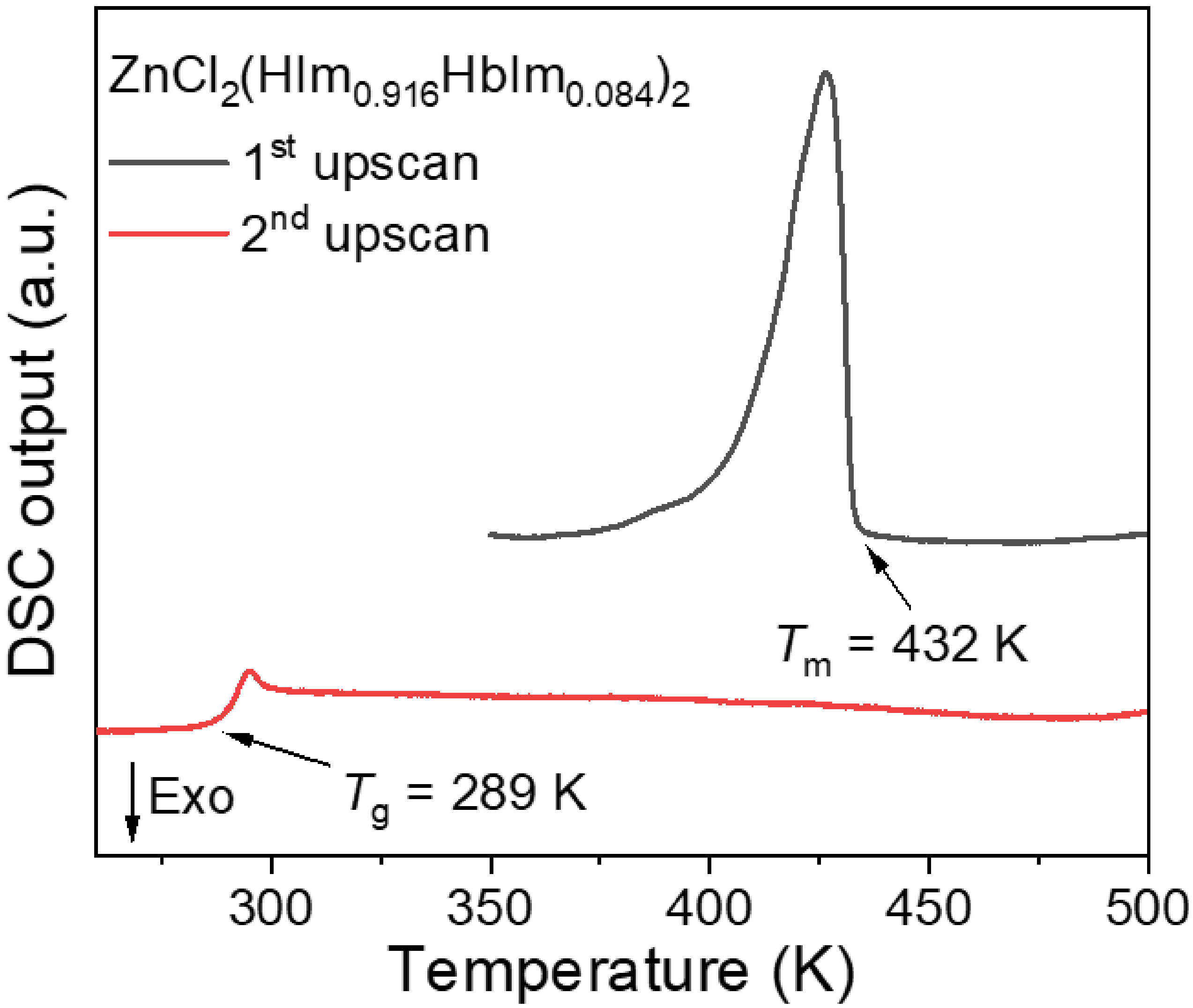


**Figure S14.** DSC upscan curves of the $ZnCl_2(HIm_{0.916}HbIm_{0.084})_2$ in the eutectic system.

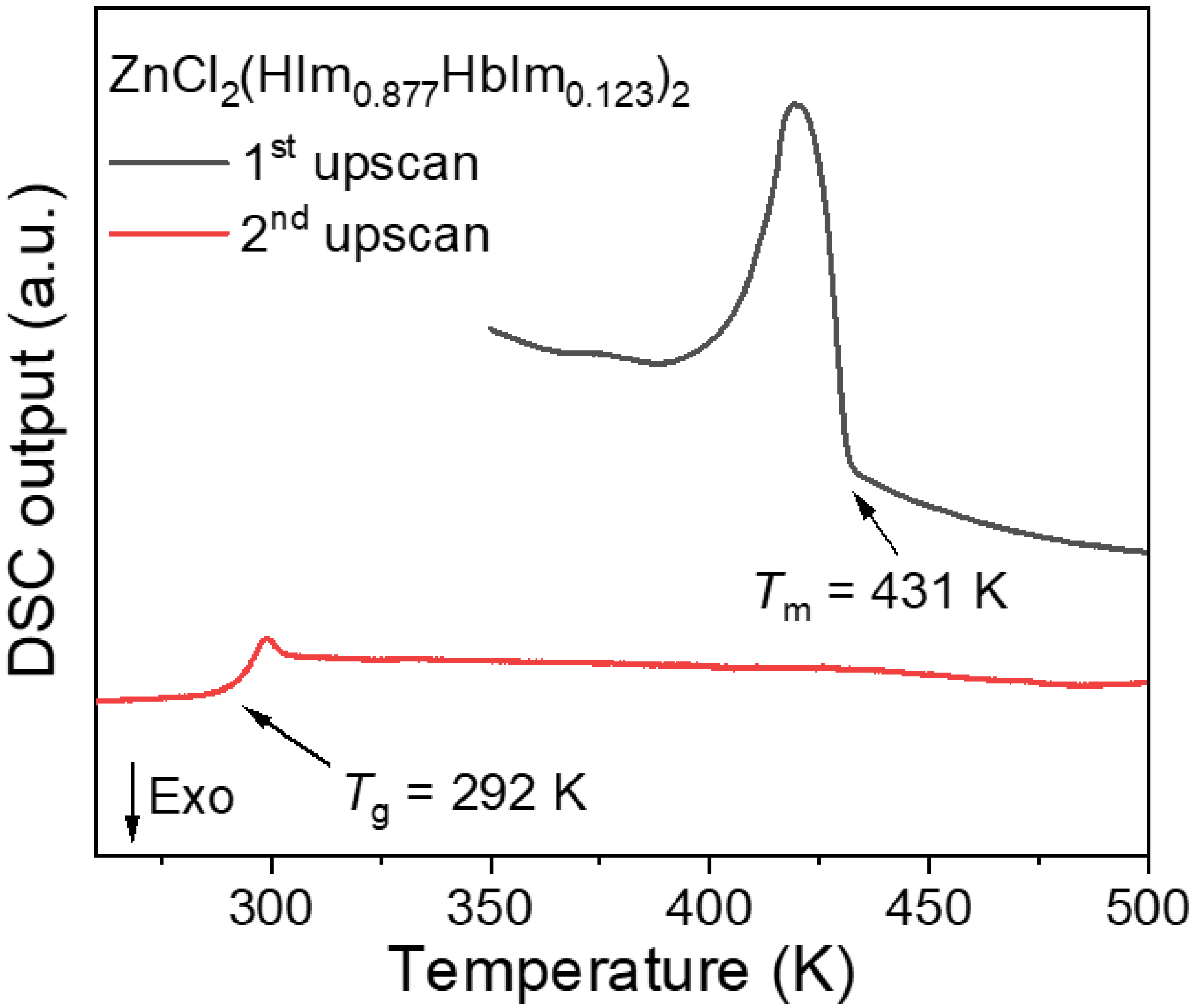


**Figure S15.** DSC upscan curves of the $ZnCl_2(HIm_{0.877}HbIm_{0.123})_2$ in the eutectic system.

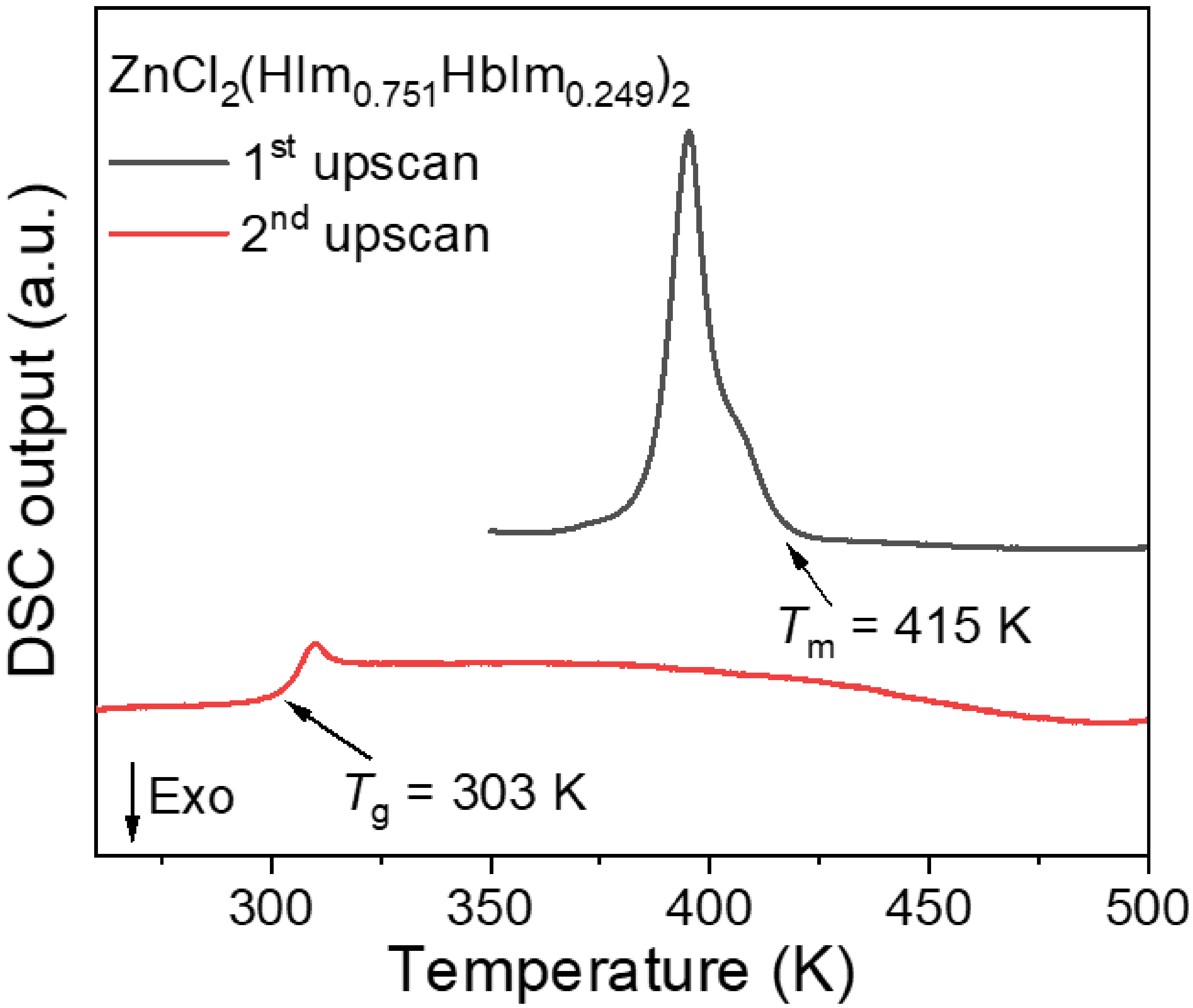


**Figure S16.** DSC upscan curves of the $ZnCl_2(HIm_{0.751}HbIm_{0.249})_2$ in the eutectic system.

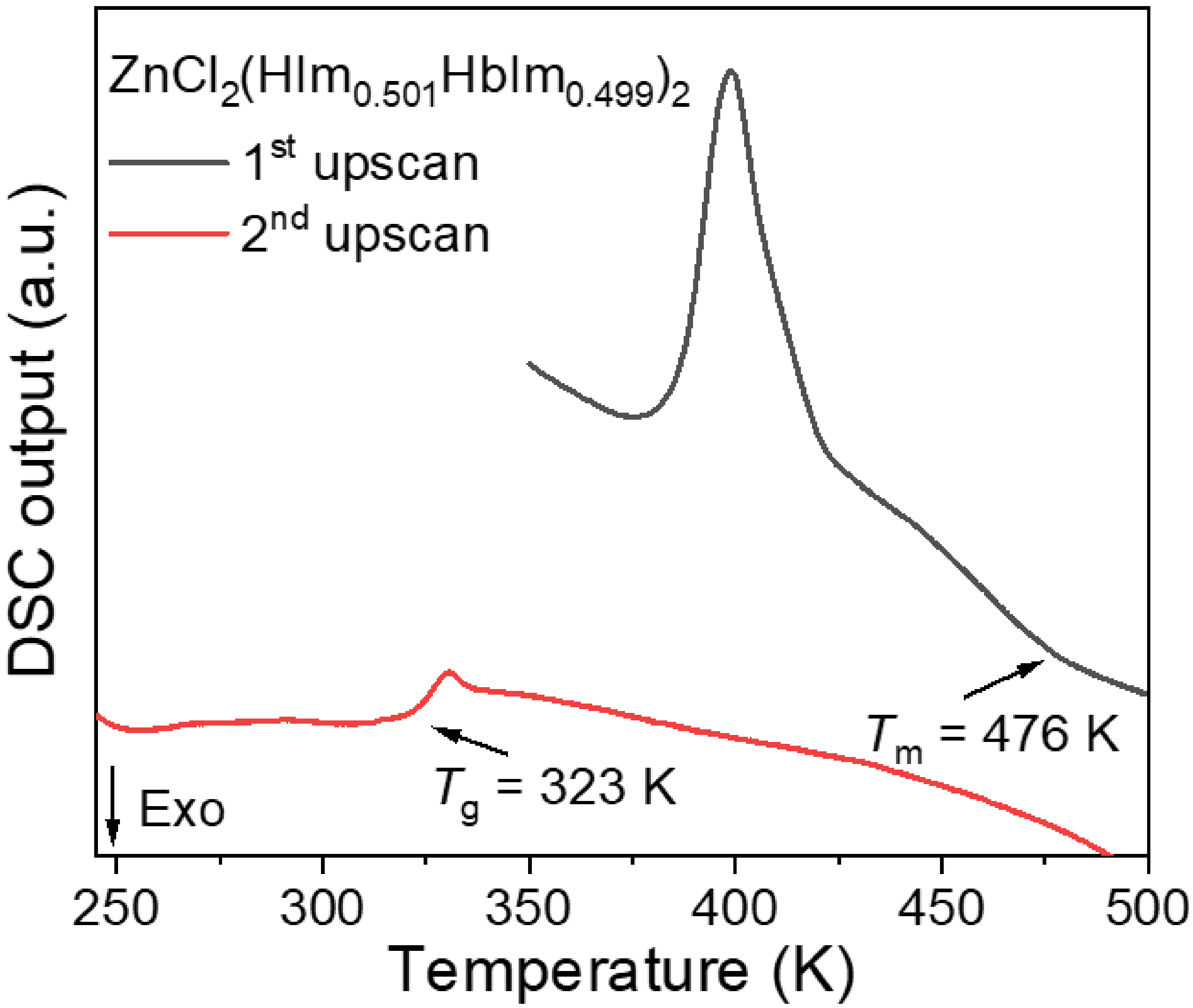


**Figure S17.** DSC upscan curves of the $ZnCl_2(HIm_{0.501}HbIm_{0.499})_2$ in the eutectic system.

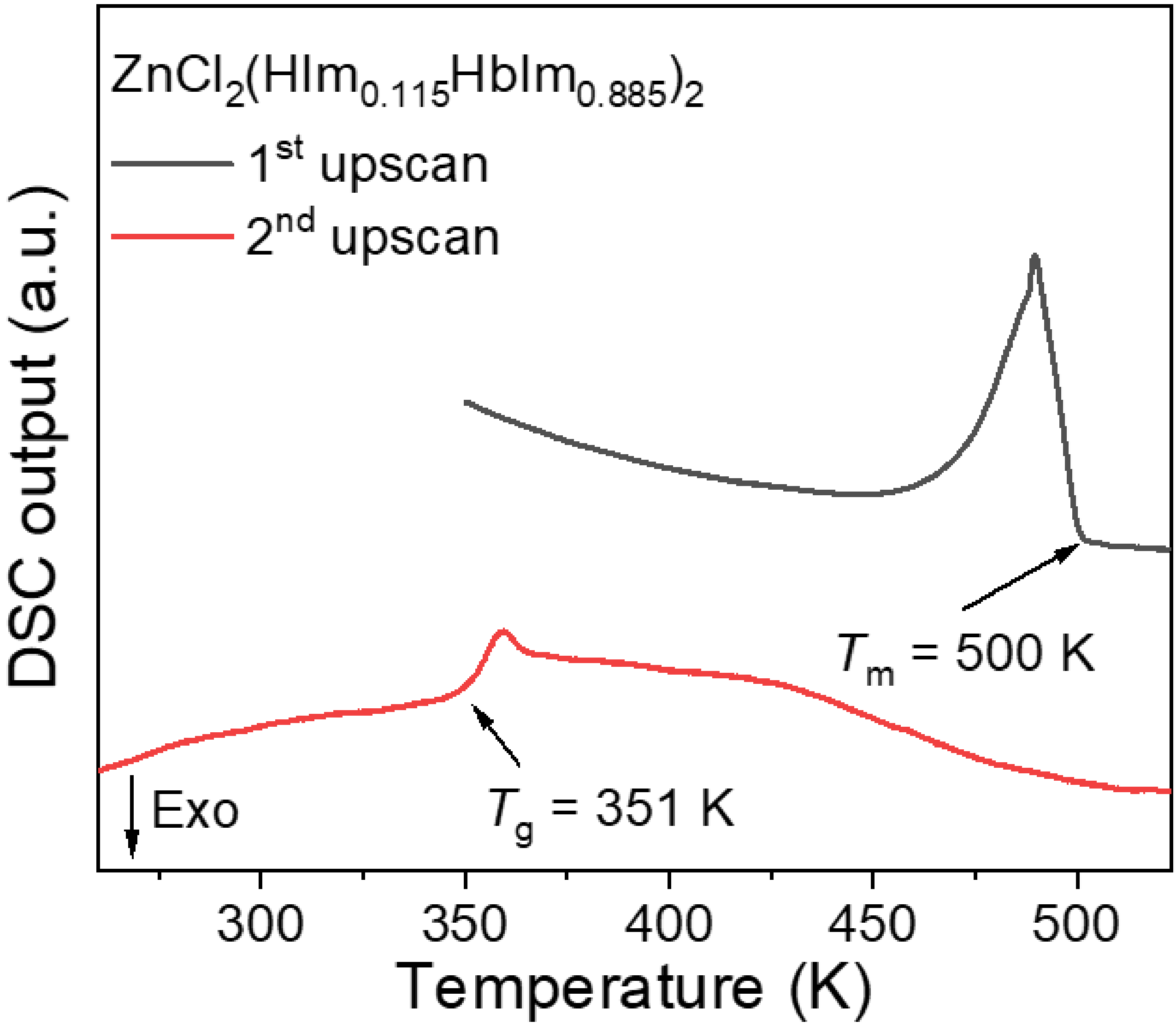


**Figure S18.** DSC upscan curves of the $ZnCl_2(HIm_{0.115}HbIm_{0.885})_2$ in the eutectic system.

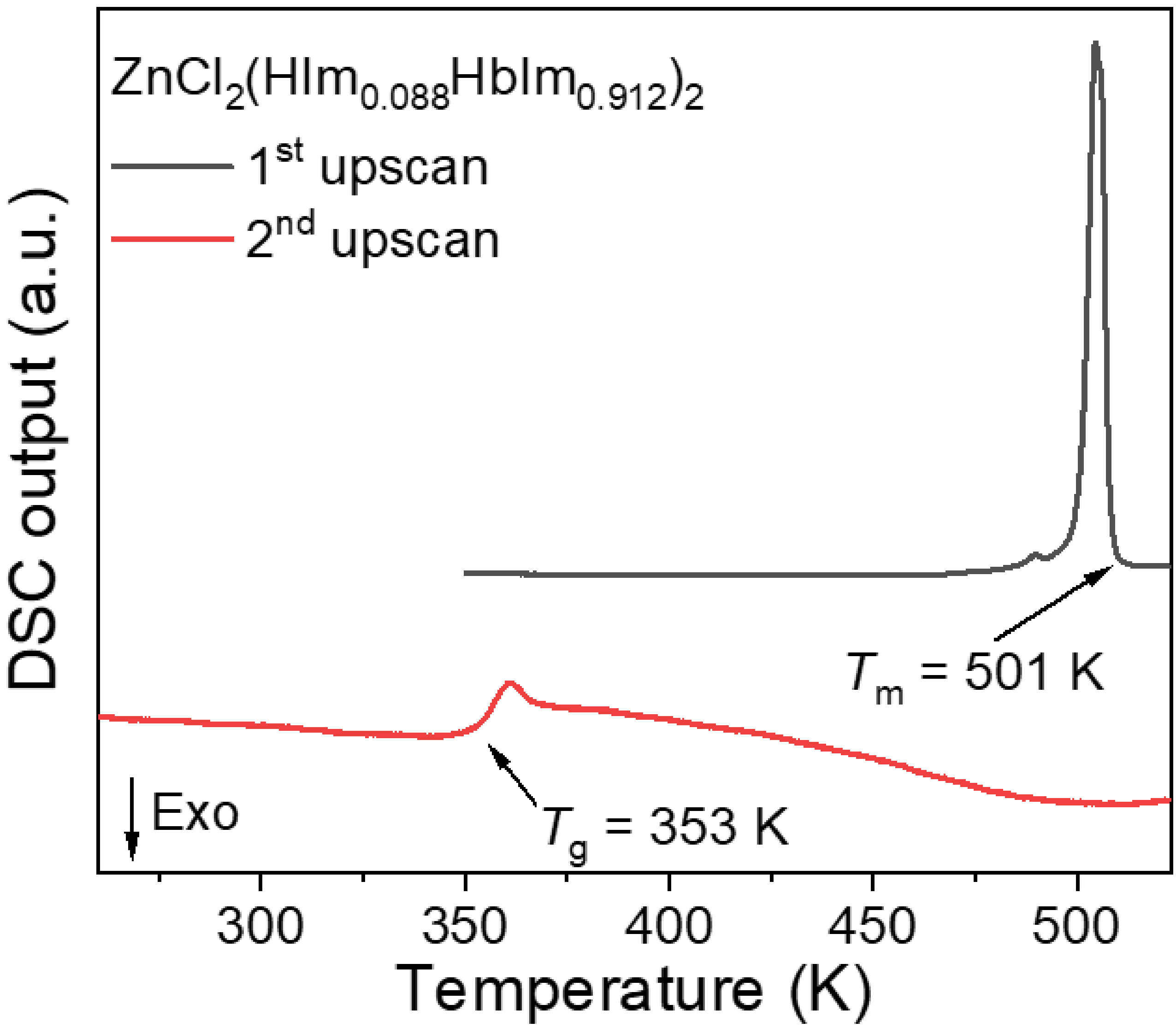


**Figure S19.** DSC upscan curves of the $ZnCl_2(HIm_{0.088}HbIm_{0.912})_2$ in the eutectic system.

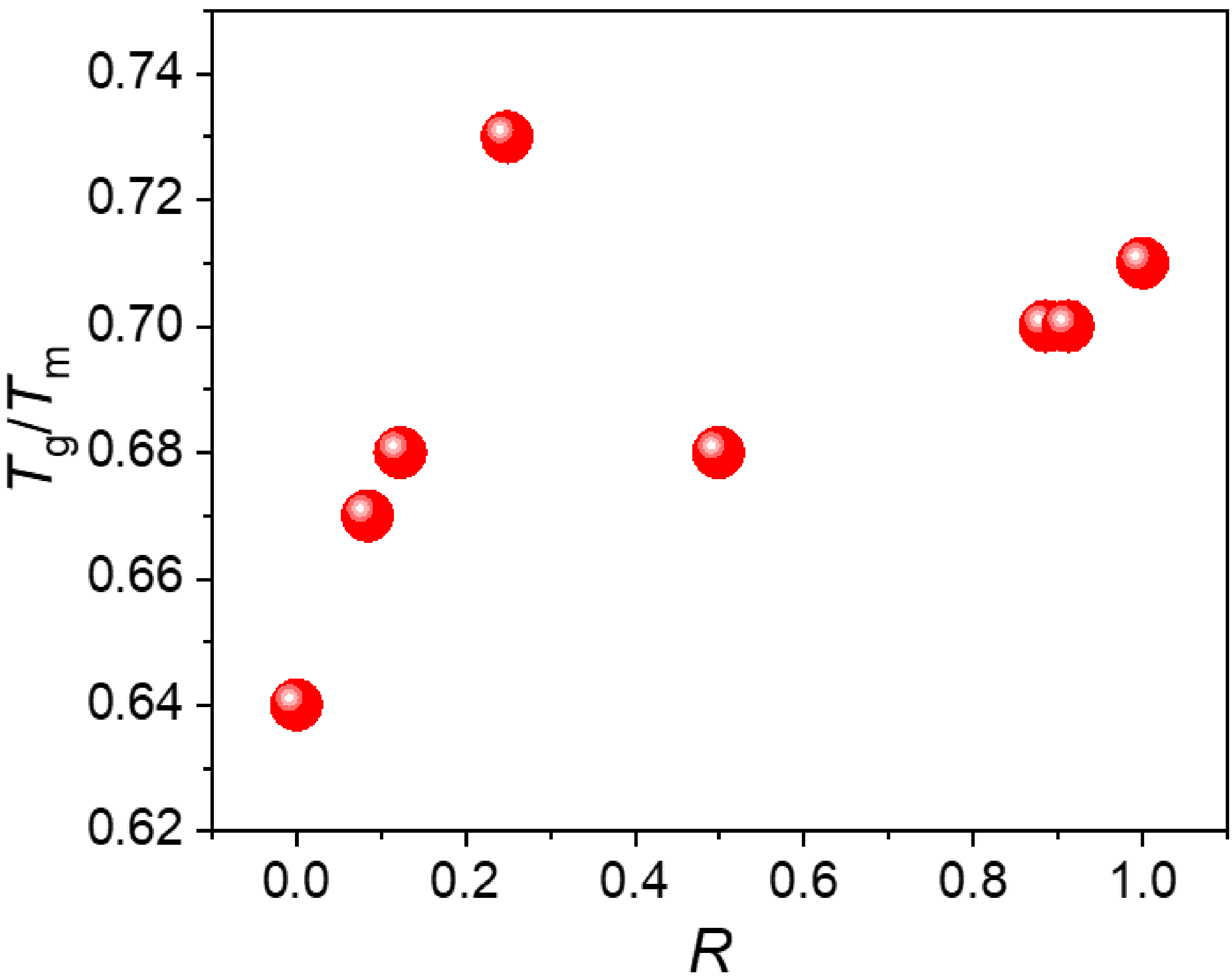


**Figure S20.** Relationship between the $T_g/T_m$ value and and molar ratio of $R$ = HbIm/(HIm + HbIm) in the binary eutectic system.

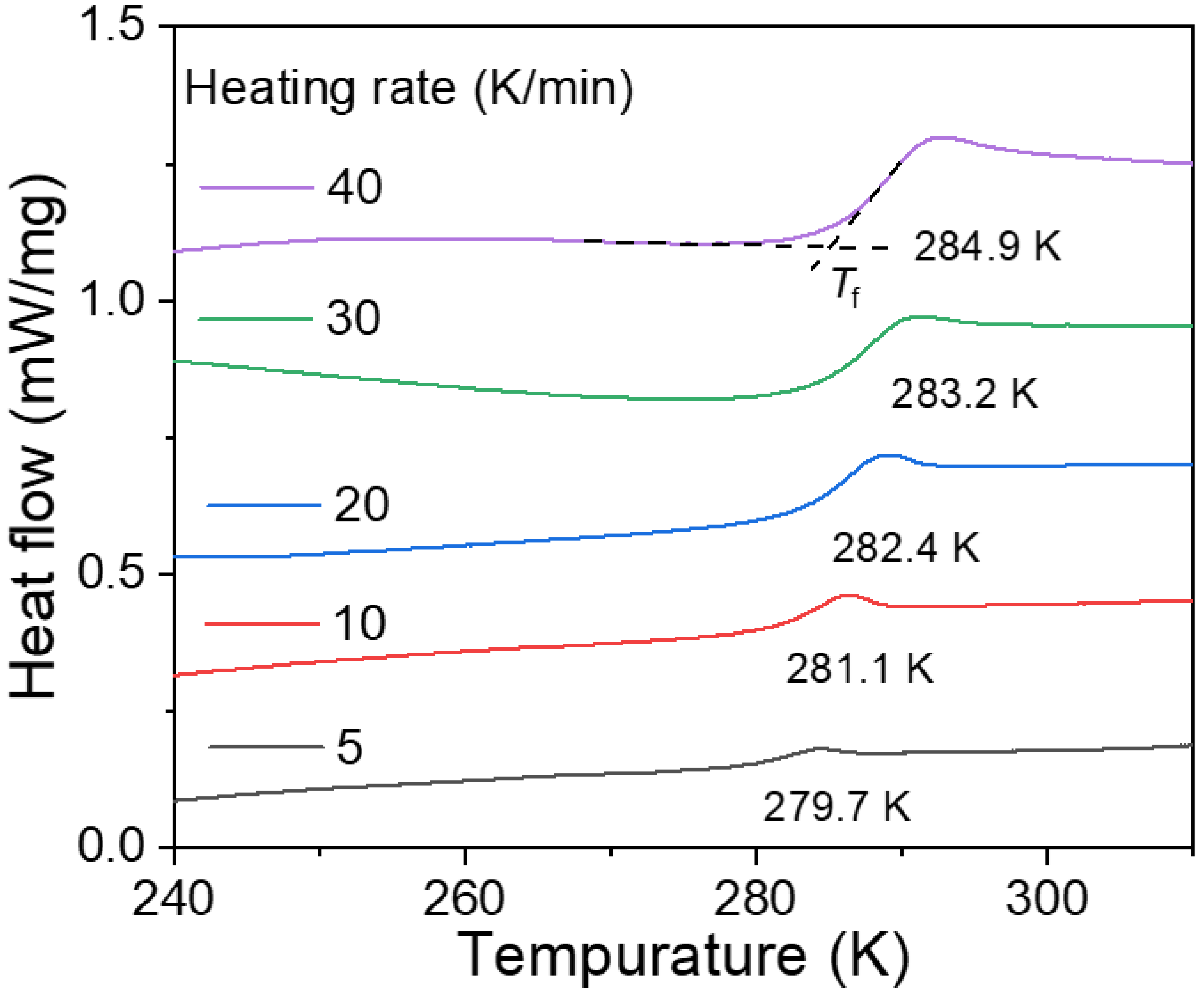


**Figure S21.** Variable heating rate DSC scans of MOIC-4 were recorded at heating rates of 5, 10, 20, 30, and 40 K·min$^{-1}$, and the fictive temperature ($T_f$) was determined by the extrapolation method.

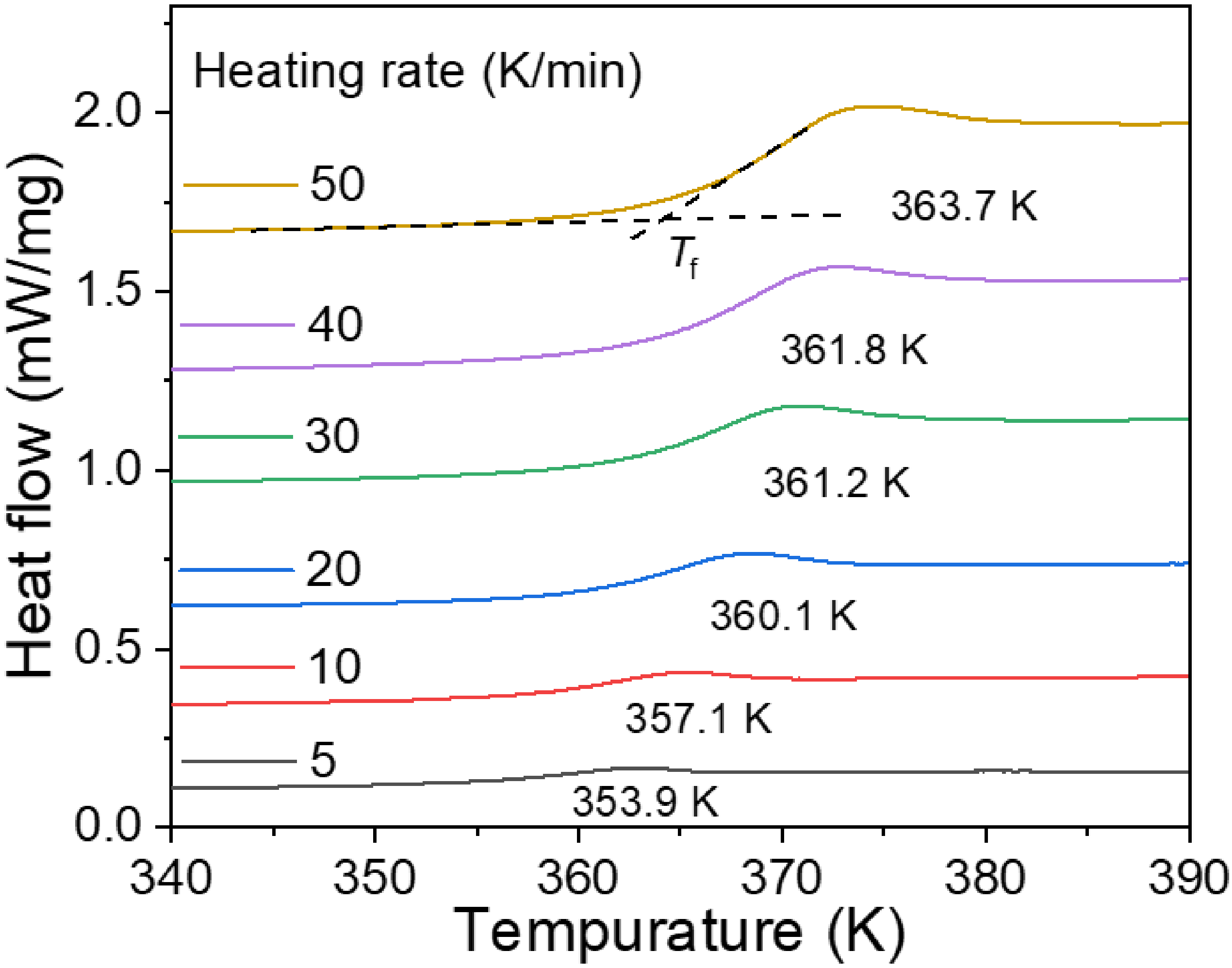


**Figure S22.** Variable heating rate DSC scans of MOIC-7 were recorded at heating rates of 5, 10, 20, 30, 40, and 50 K·min$^{-1}$, and the fictive temperature ($T_f$) was determined by the extrapolation method.

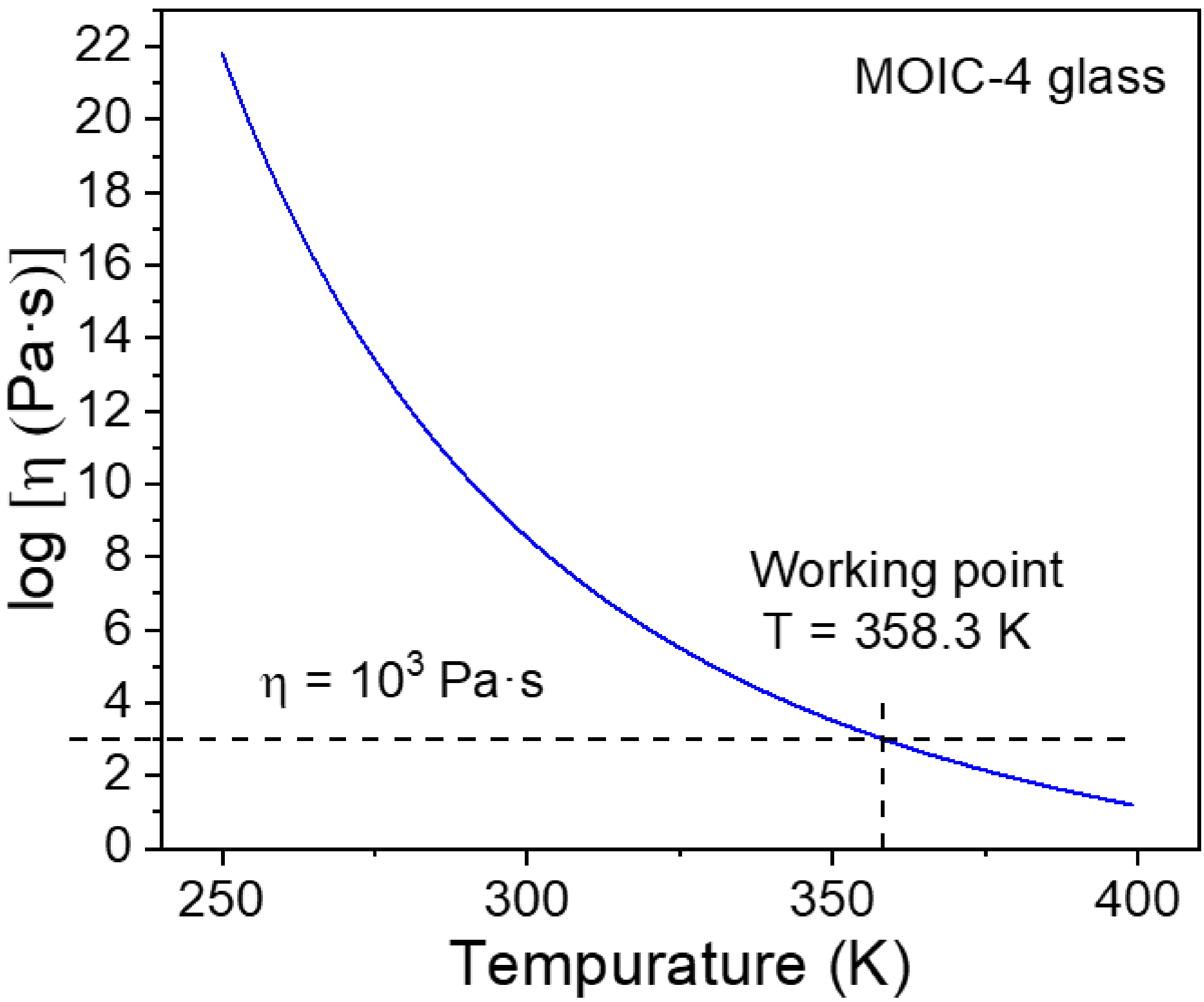


**Figure S23.** Theoretical viscosity-temperature curve for MOIC-4 glass.

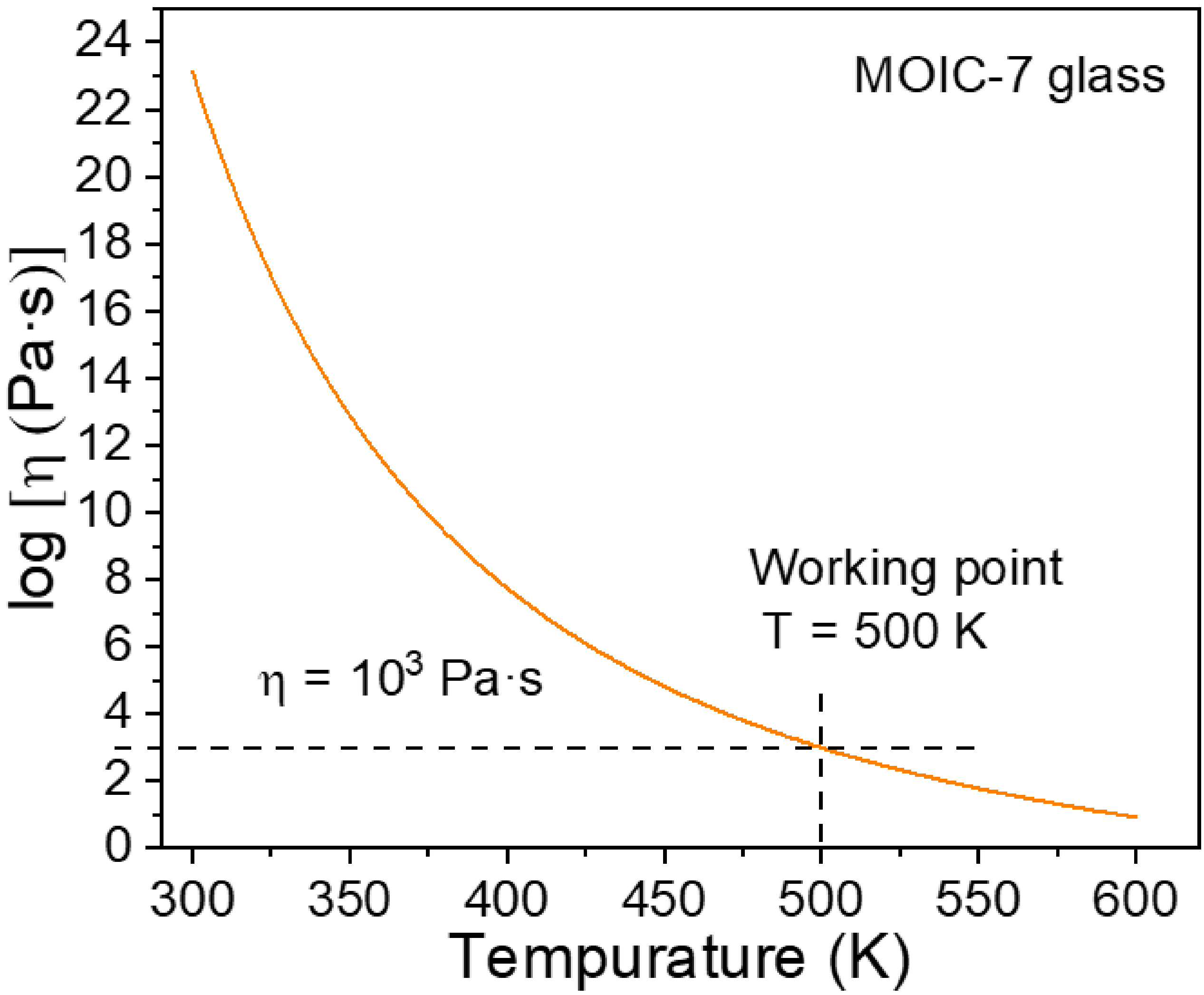


**Figure S24.** Theoretical viscosity-temperature curve for MOIC-7 glass.

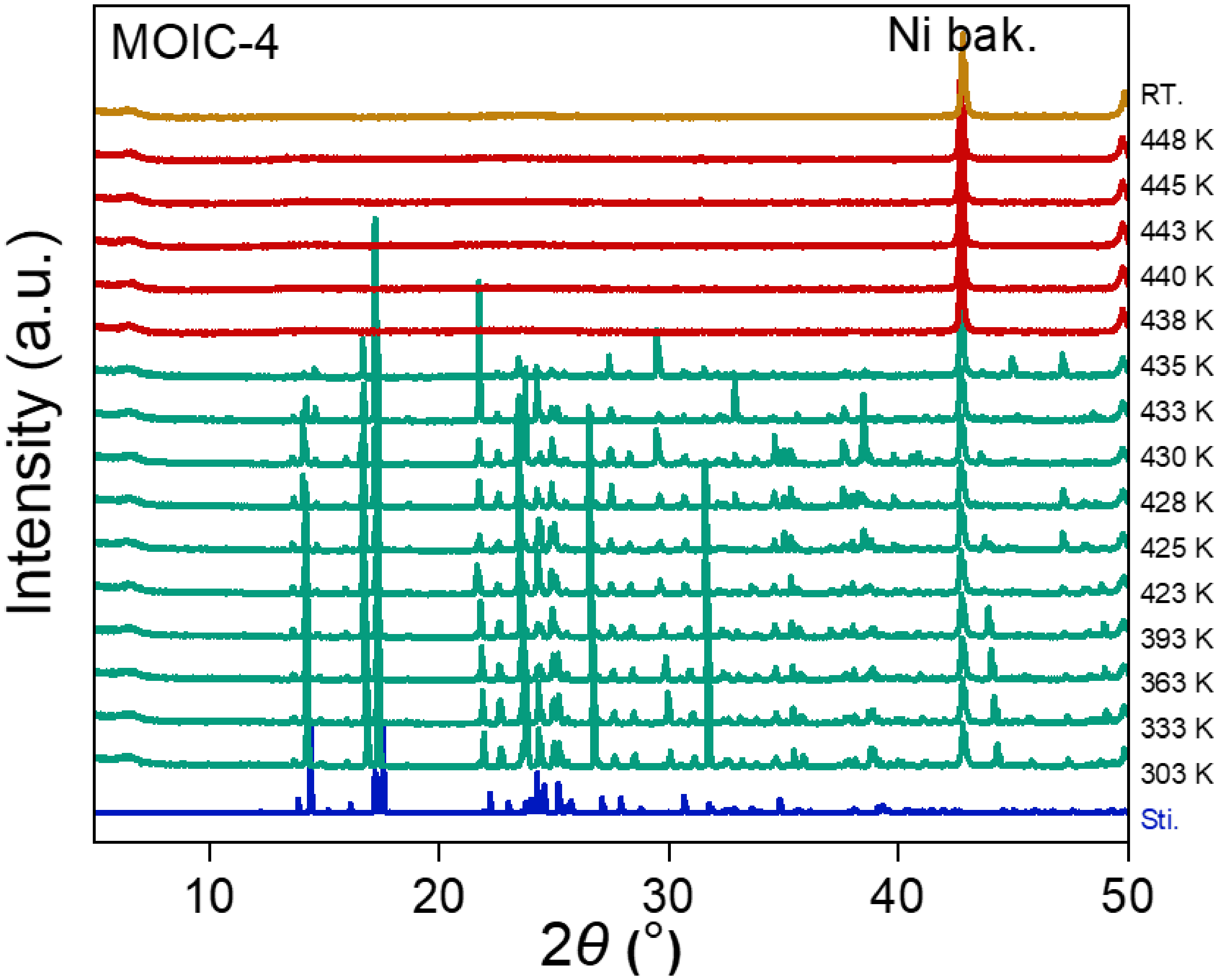


**Figure S25.** Variable-temperature XRD patterns of MOIC-4.

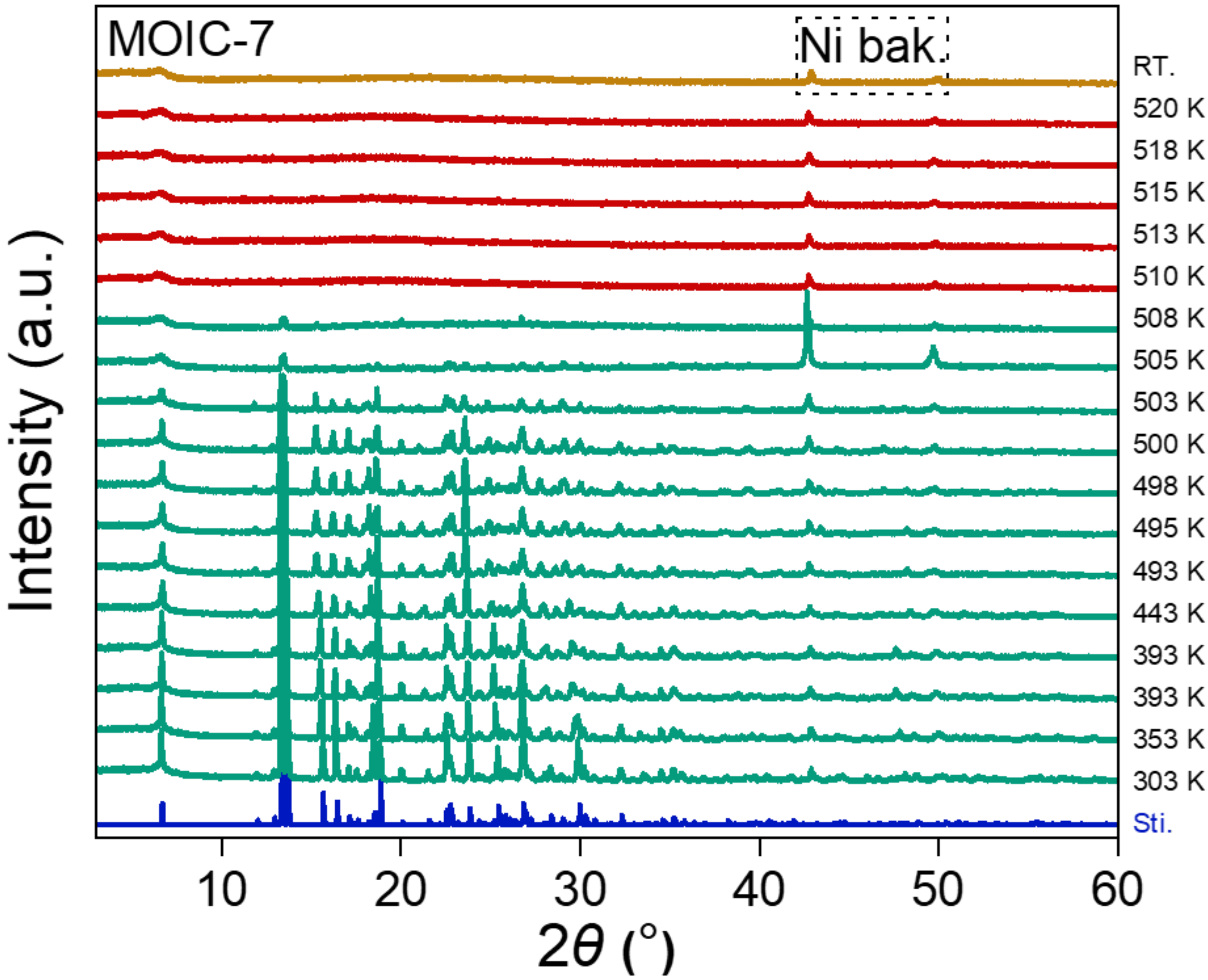


**Figure S26.** Variable-temperature XRD patterns of MOIC-7.

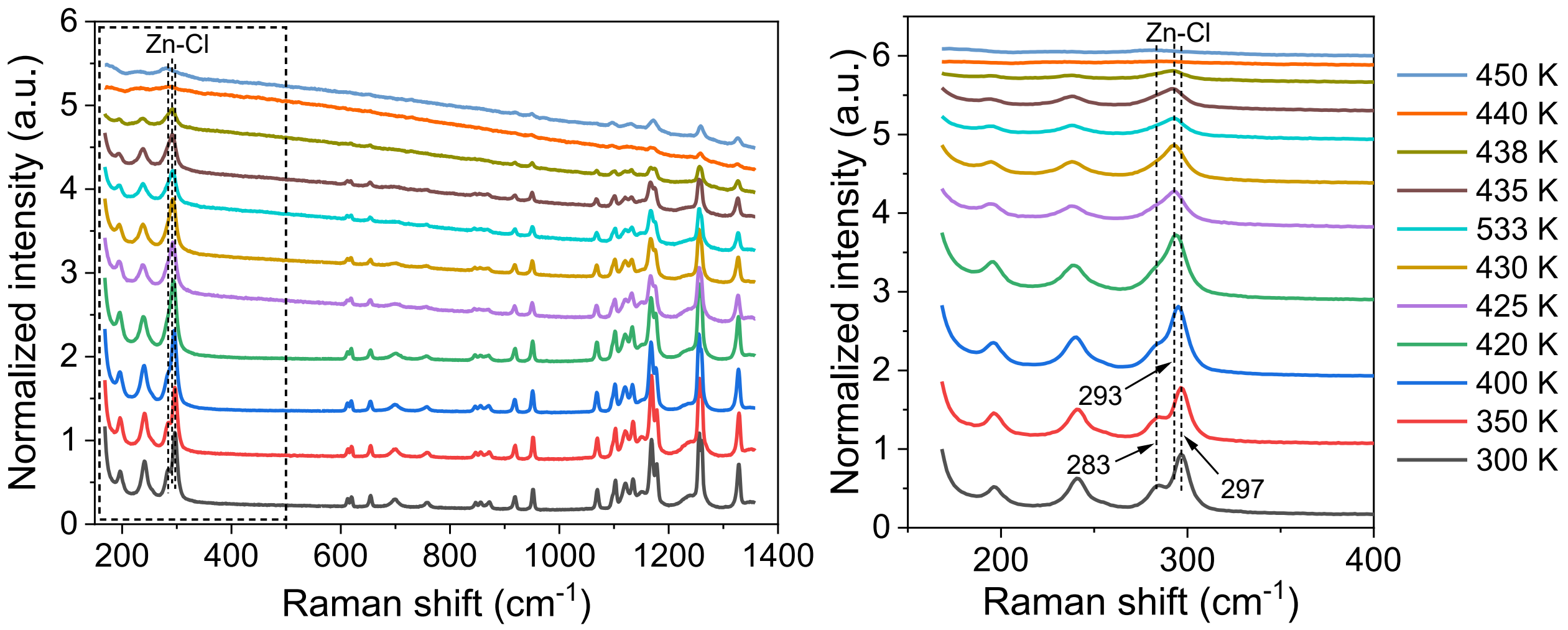


**Figure S27.** *In-situ* Raman spectra of MOIC-4 during phase transition (left panel) and Enlarged Raman spectra (right panel) of the selected areas of Fig. S27.

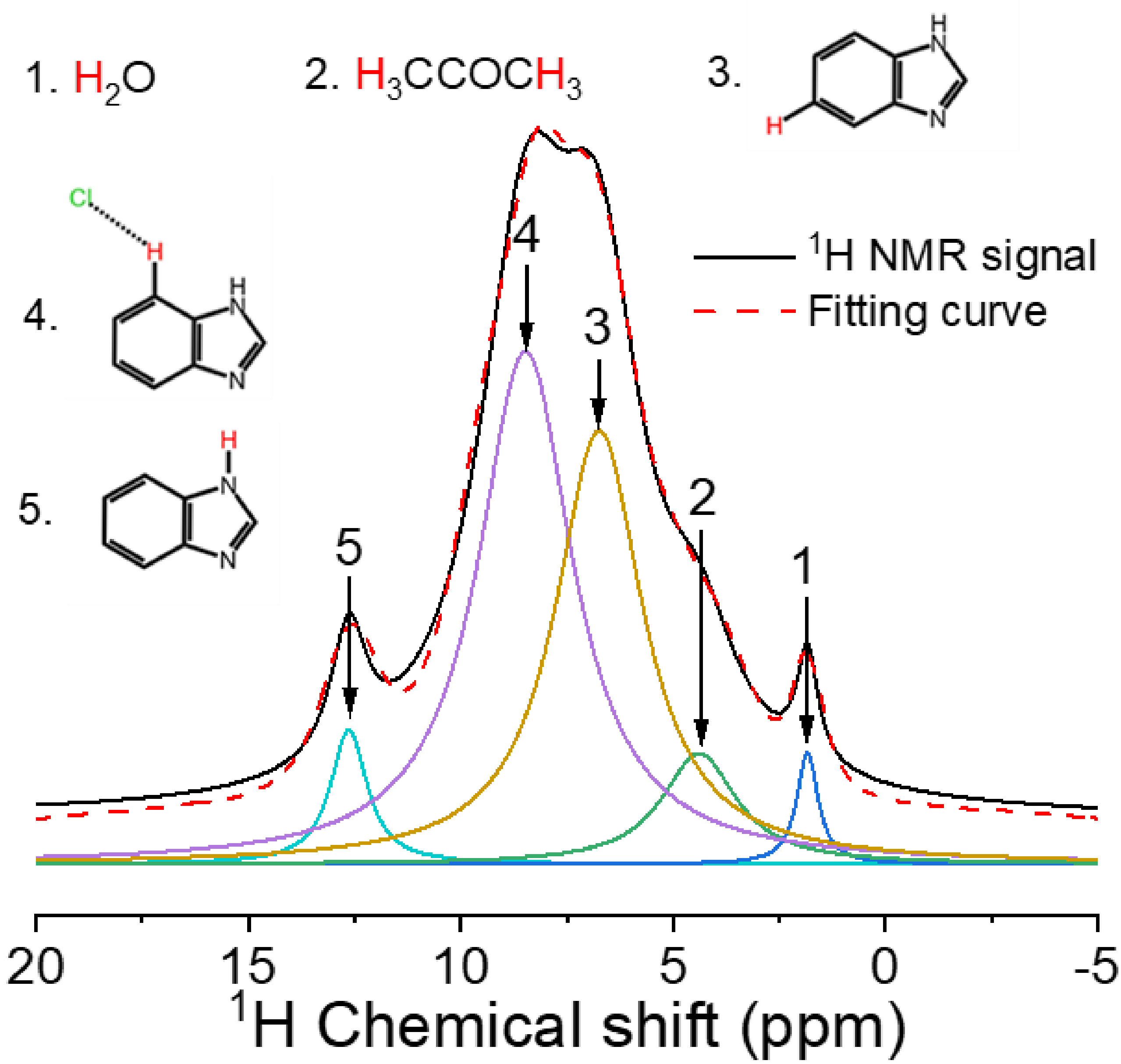


**Figure S28.** The deconvolution of the $^{1}H$ NMR spectra of MOIC-7 crystal.

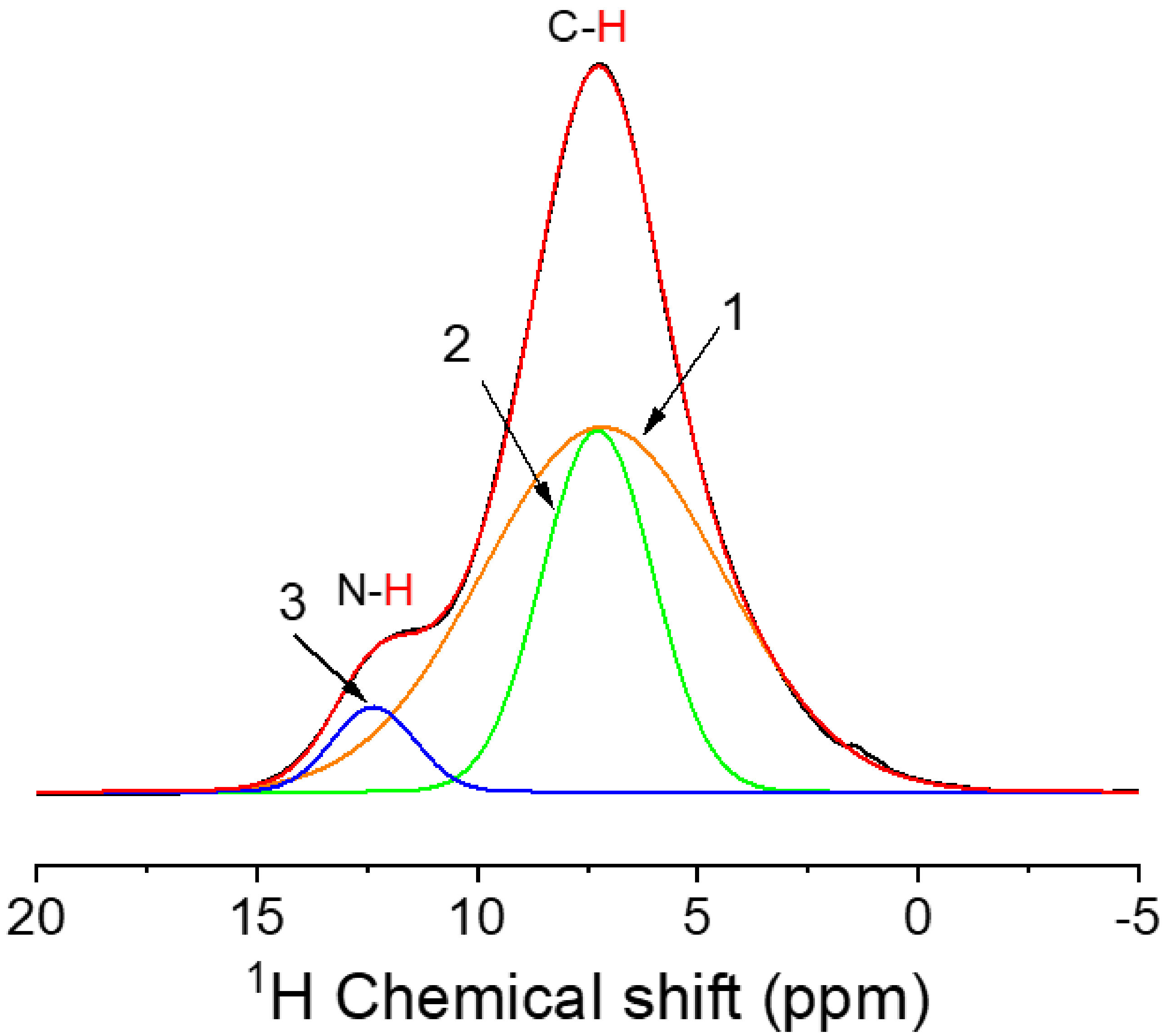


**Figure S29.** The deconvolution of the $^{1}$H NMR spectrum of MOIC-7 glass.

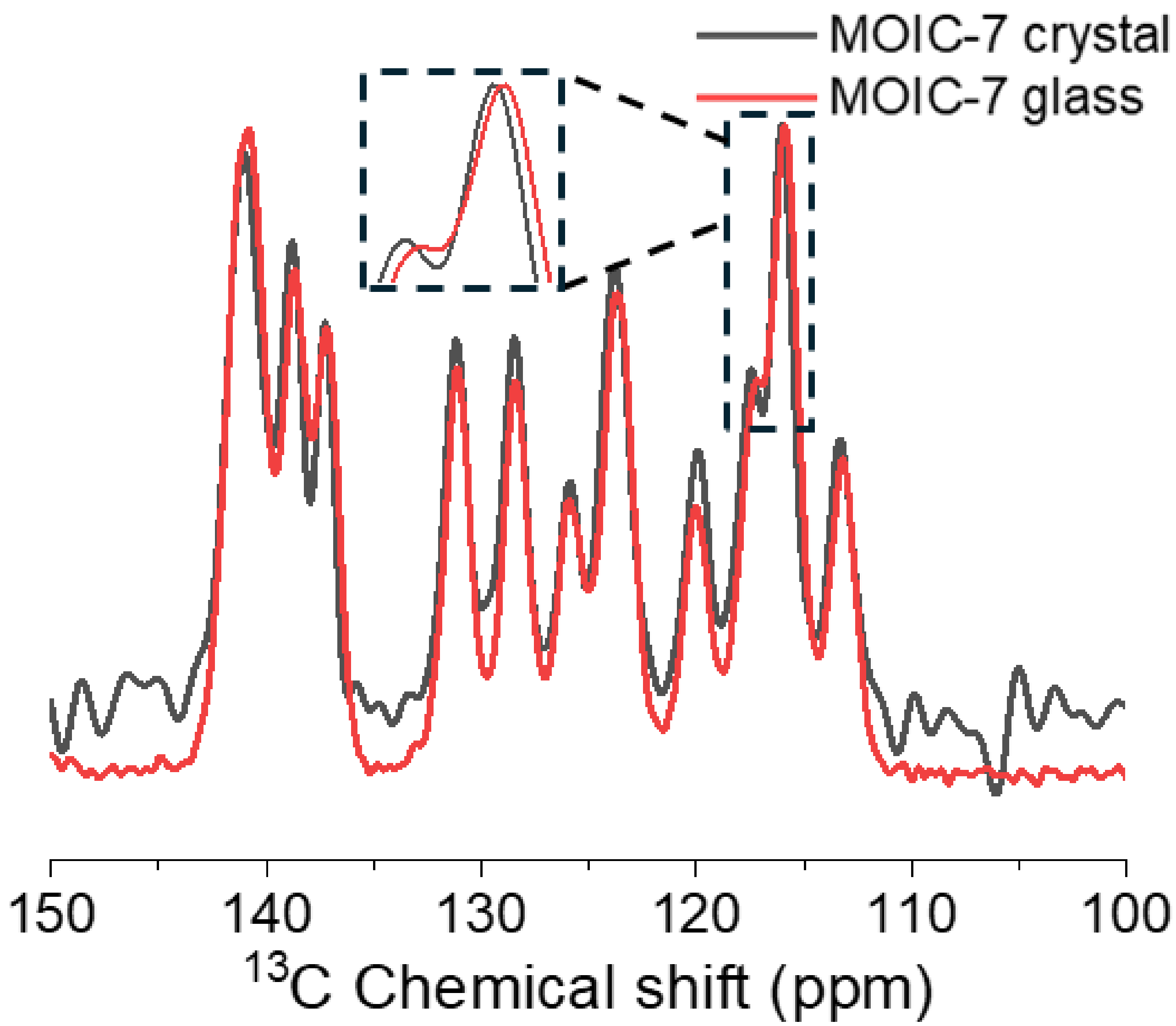


**Figure S30.** The $^{13}C$ CPMAS NMR spectra of MOIC-7 crystal and glass.

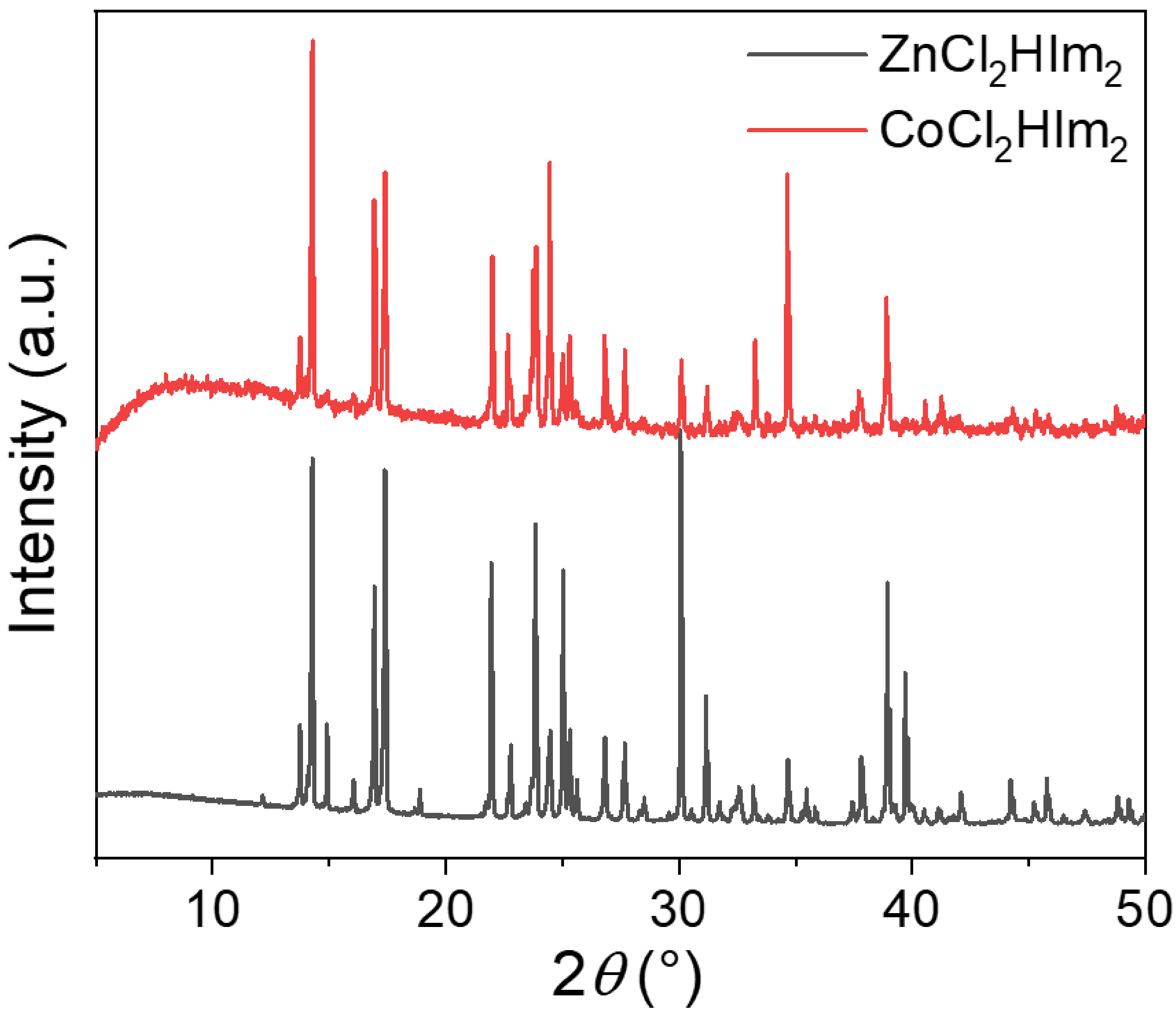


**Figure S31.** XRD patterns of MOIC-4 and its isostructural compound $CoCl_2(HIm)_2$.

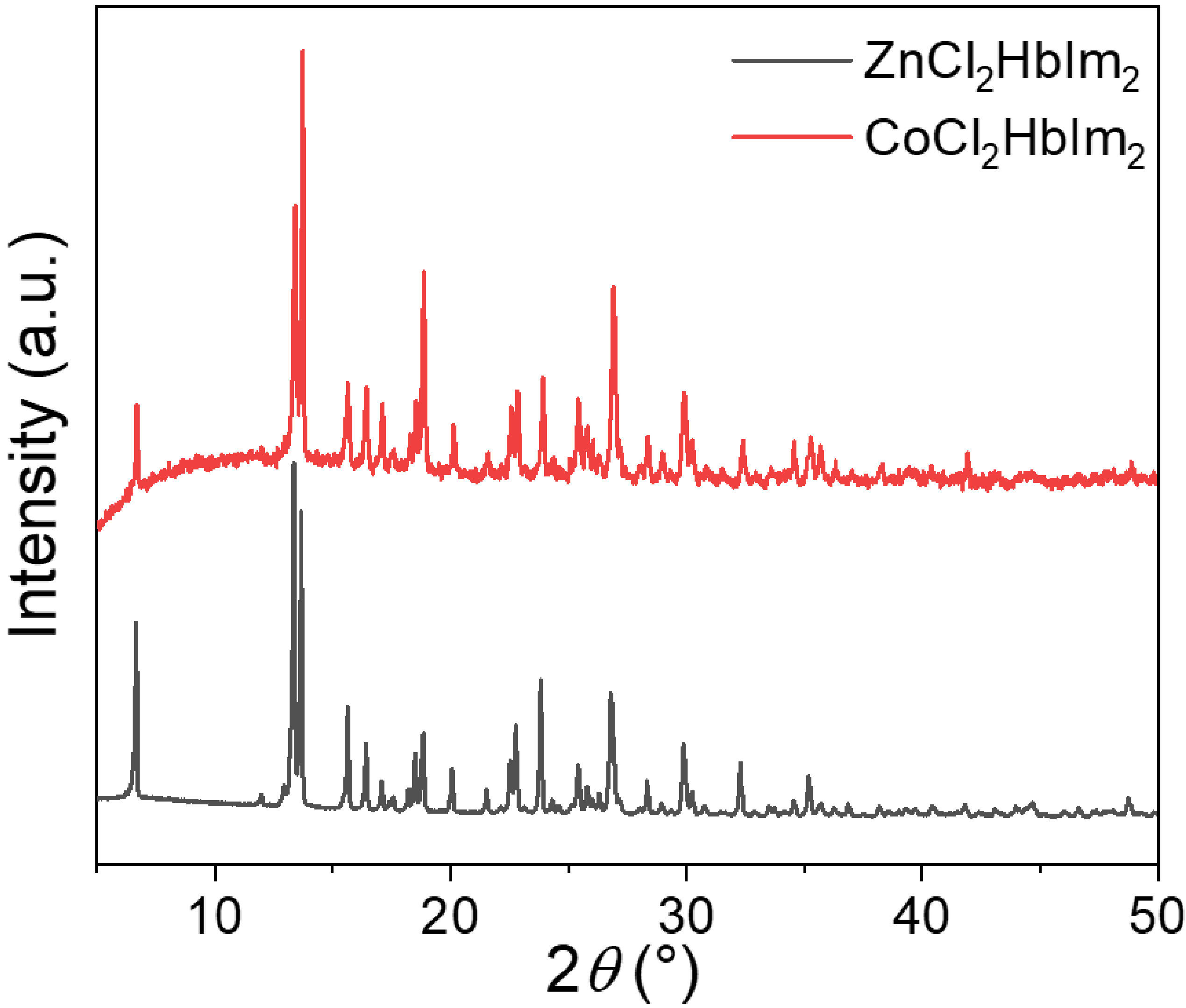


**Figure S32.** XRD patterns of MOIC-7 and its isostructural compound $CoCl_2(HbIm)_2$.

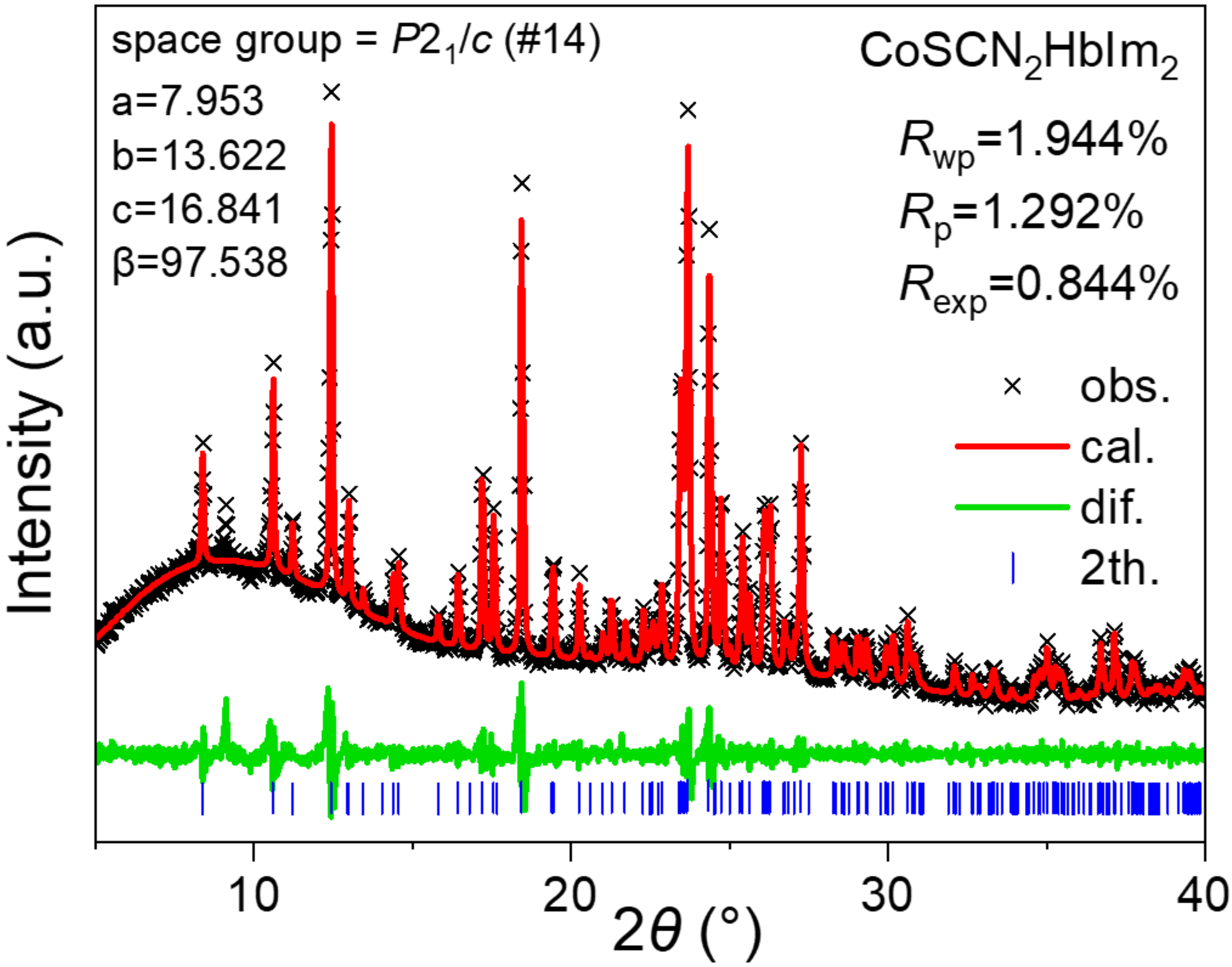


**Figure S33.** XRD refinement result of the as-synthesized $Co(SCN)_2(HbIm)_2$ crystal.

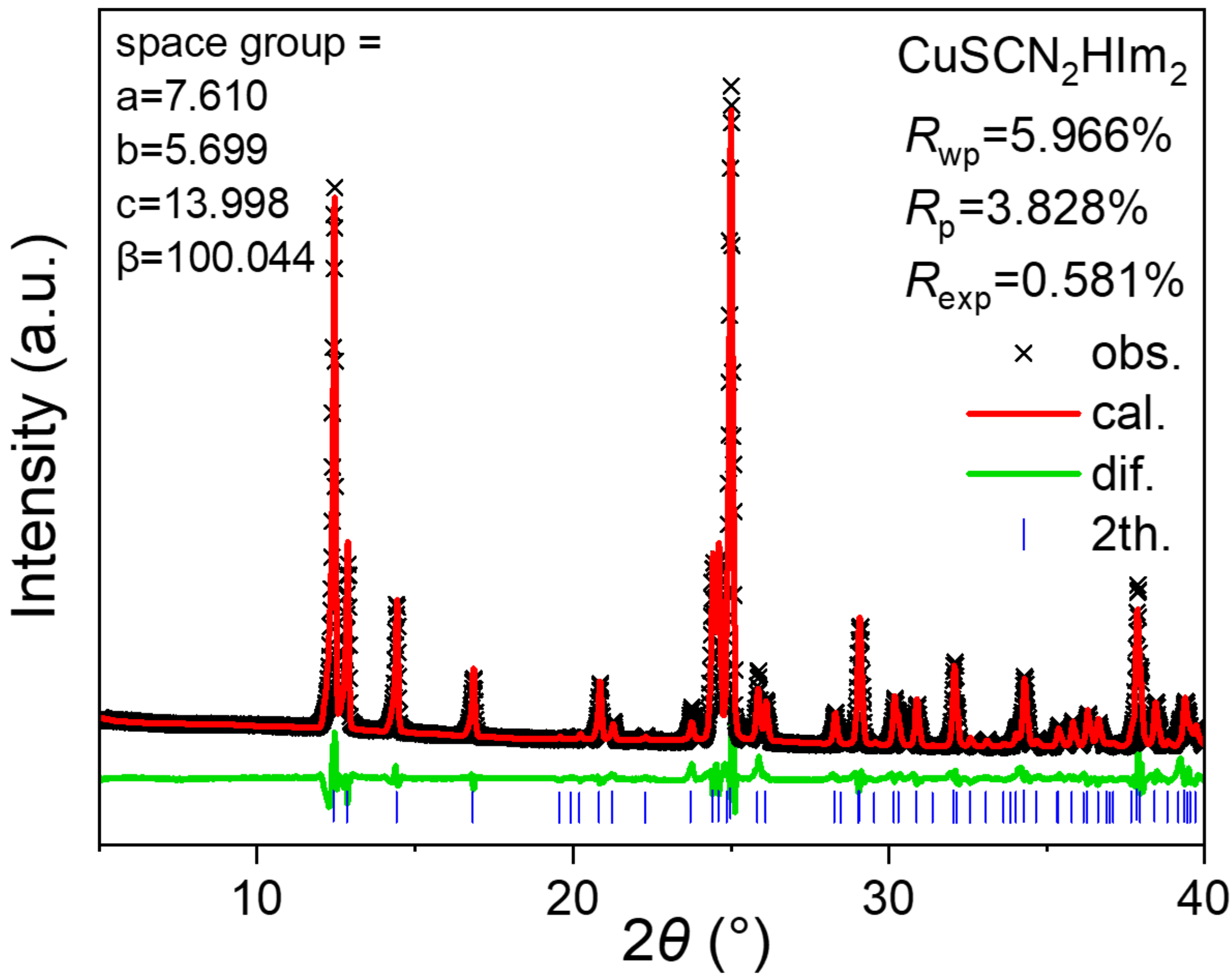


**Figure S34.** XRD refinement result of the as-synthesized $Cu(SCN)_2(HIm)_2$ crystal.

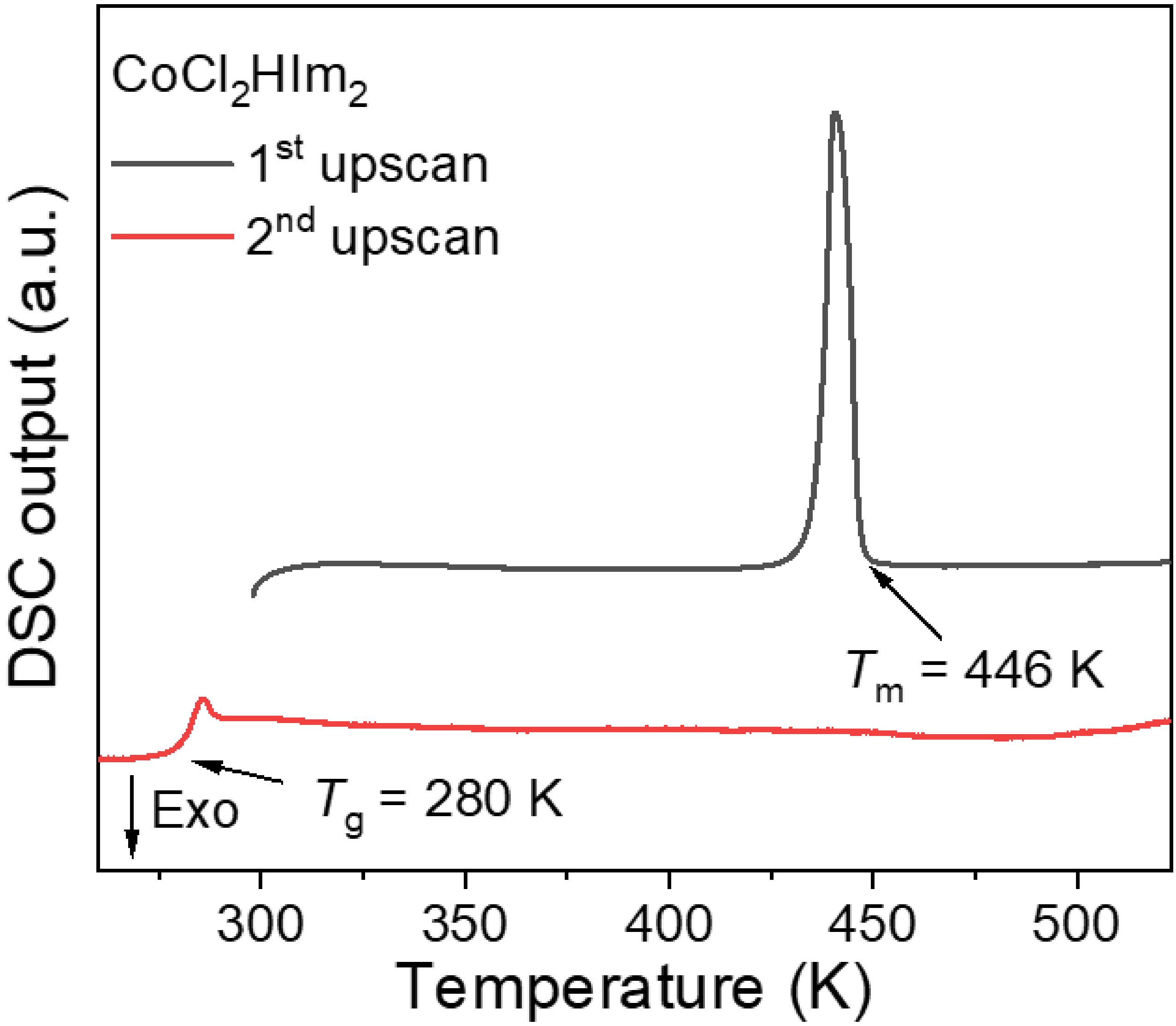


**Figure S35.** DSC upscan curves of the $CoCl_2(HIm)_2$ crystal.

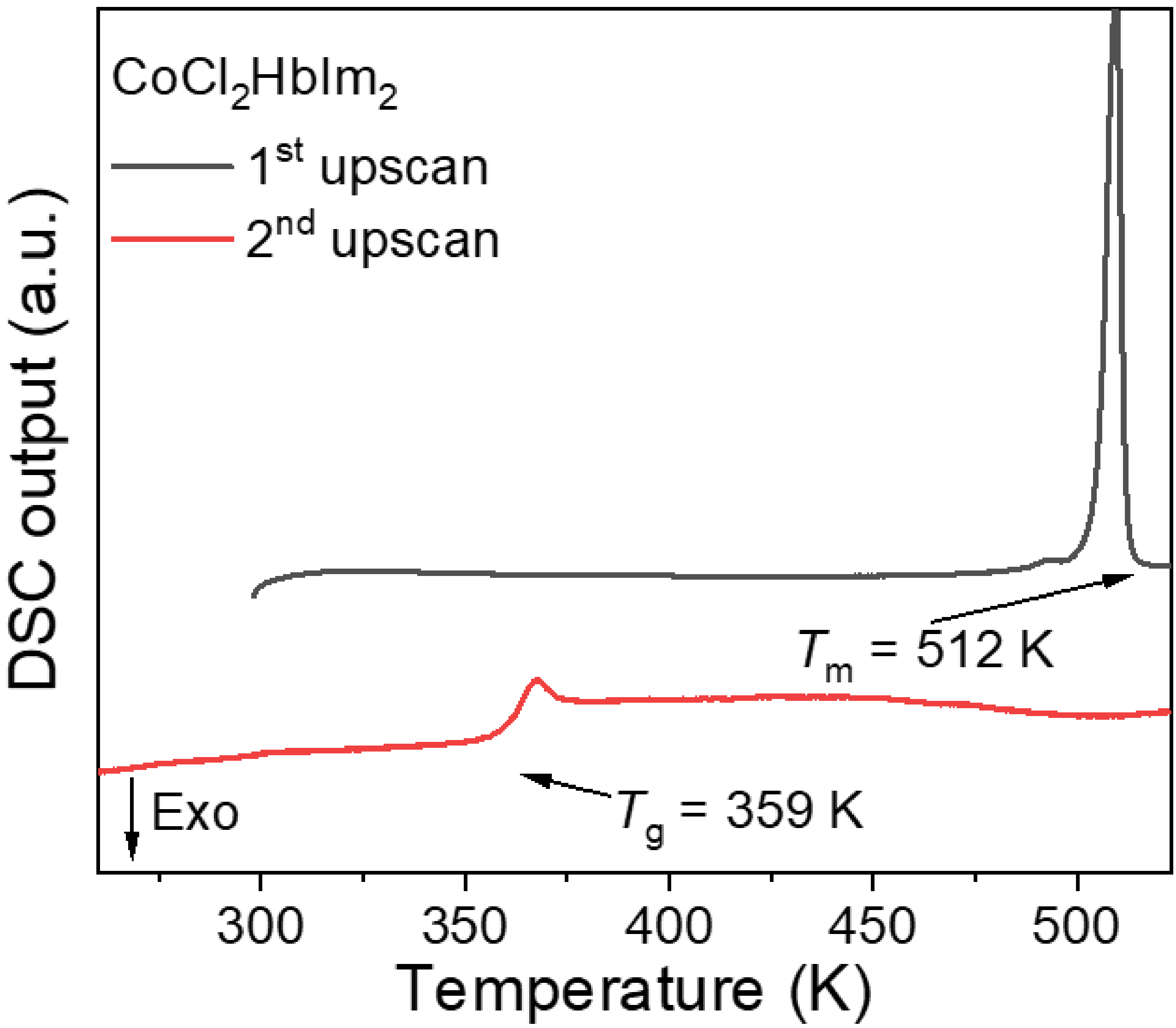


**Figure S36.** DSC upscan curves of the $CoCl_2(HbIm)_2$ crystal.

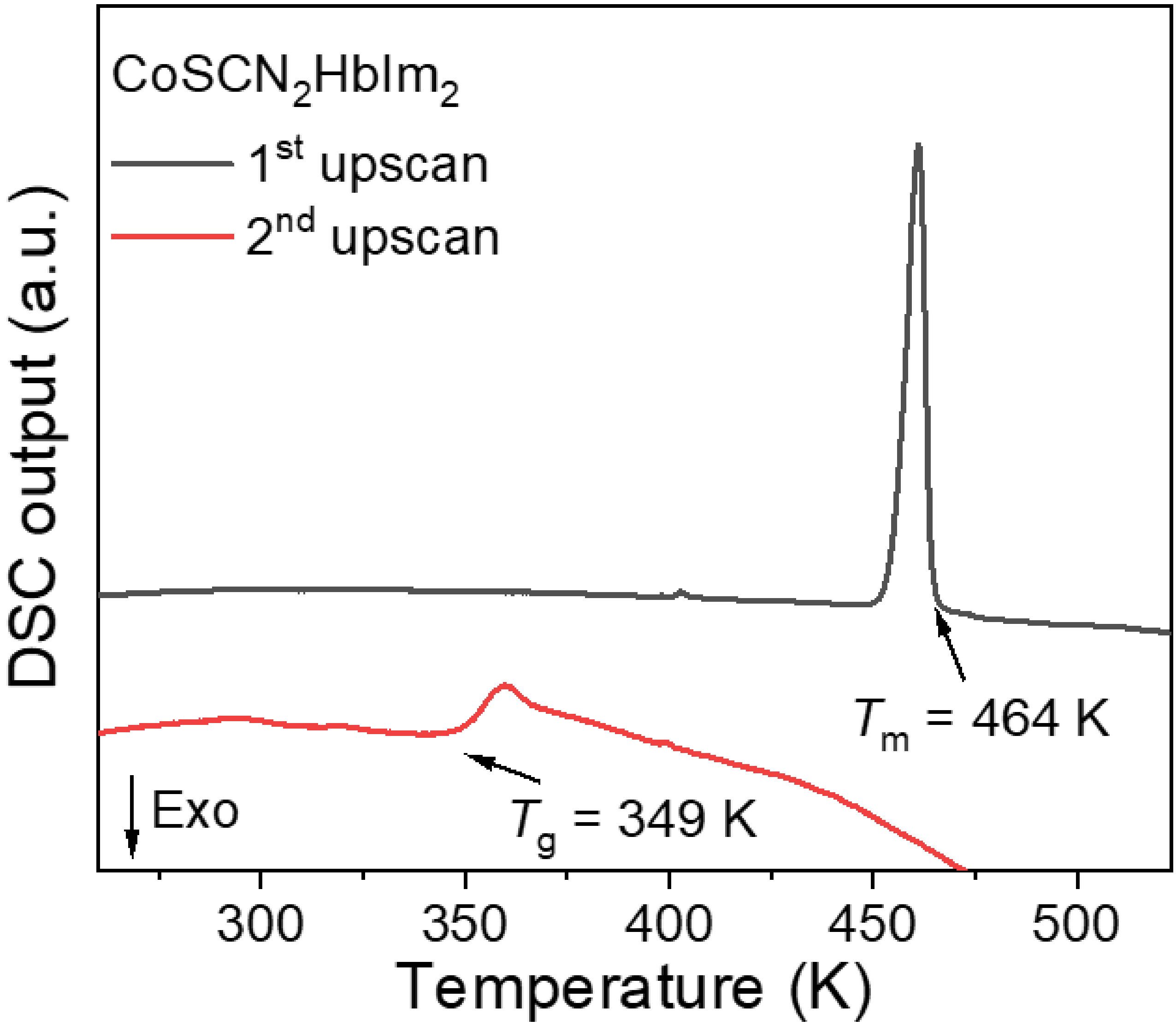


**Figure S37.** DSC upscan curves of the $Co(SCN)_2(HbIm)_2$ crystal.

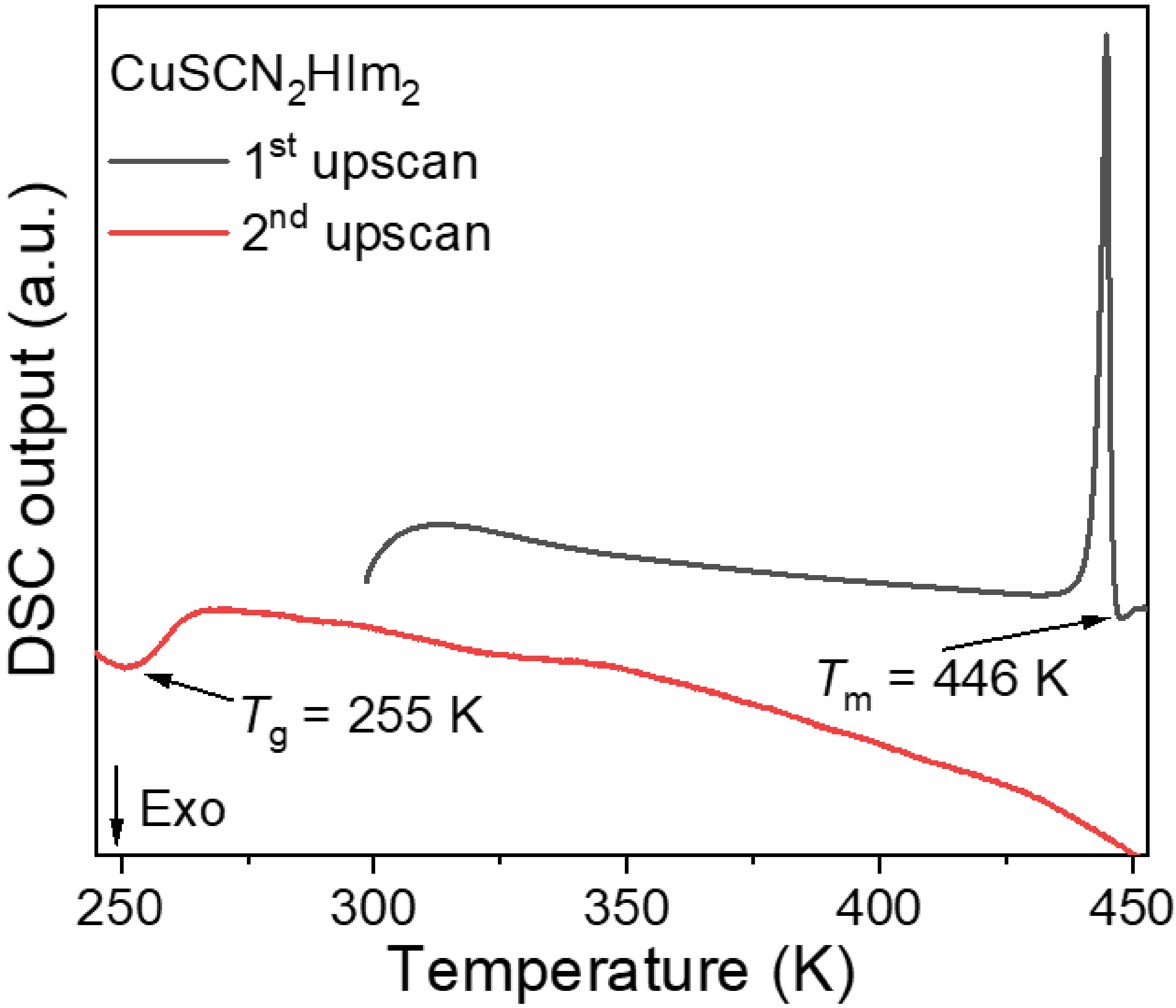


**Figure S38.** DSC upscan curves of the $Cu(SCN)_2(HIm)_2$ crystal.

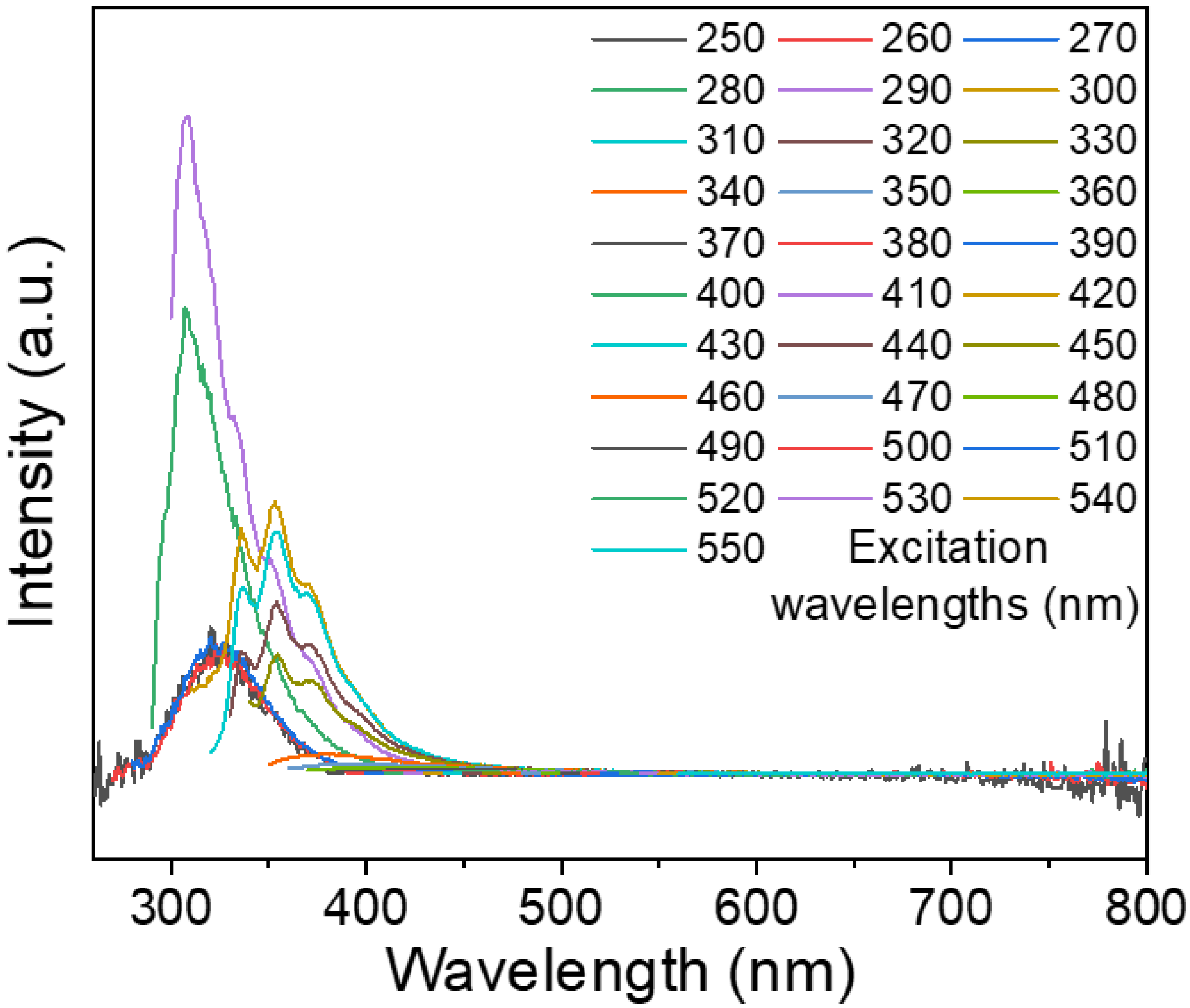


**Figure S39.** Fluorescence spectrum of MOIC-7 crystal at different excitation wavelengths.

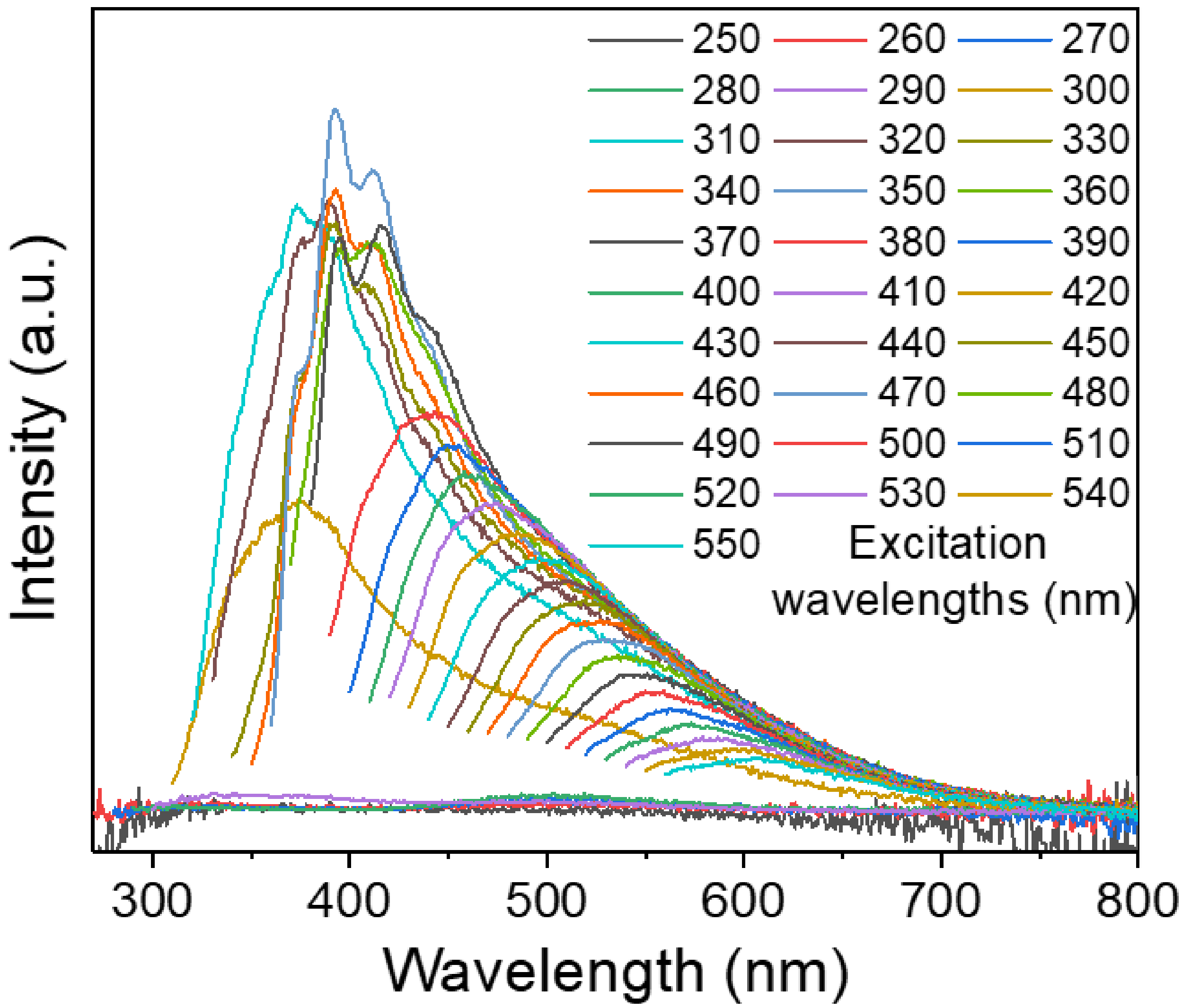


**Figure S40.** Fluorescence spectrum of MOIC-7 glass at different excitation wavelengths.

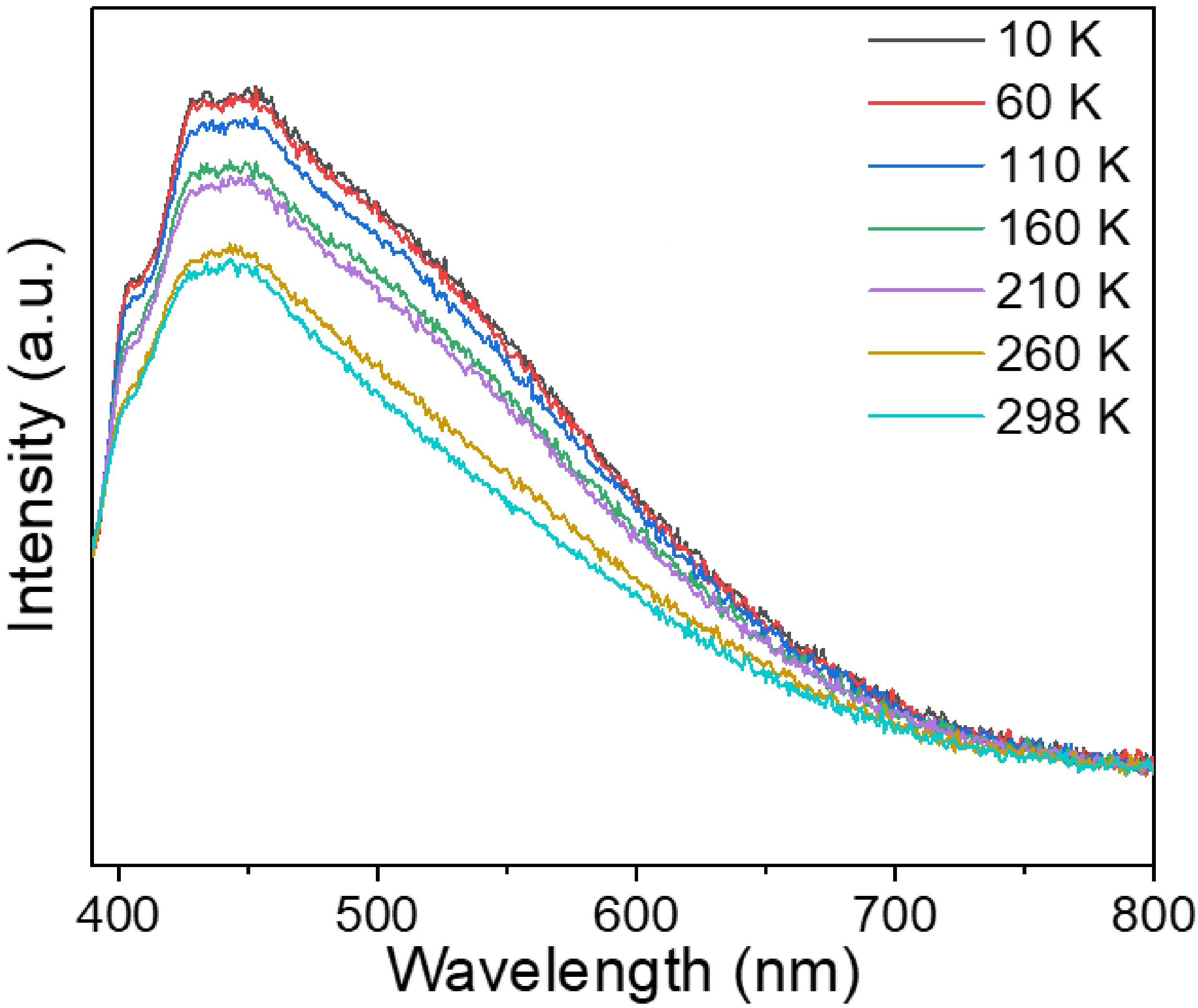


**Figure S41.** Fluorescence spectra of MOIC-7 glass at different temperatures (excitation light: 365nm).

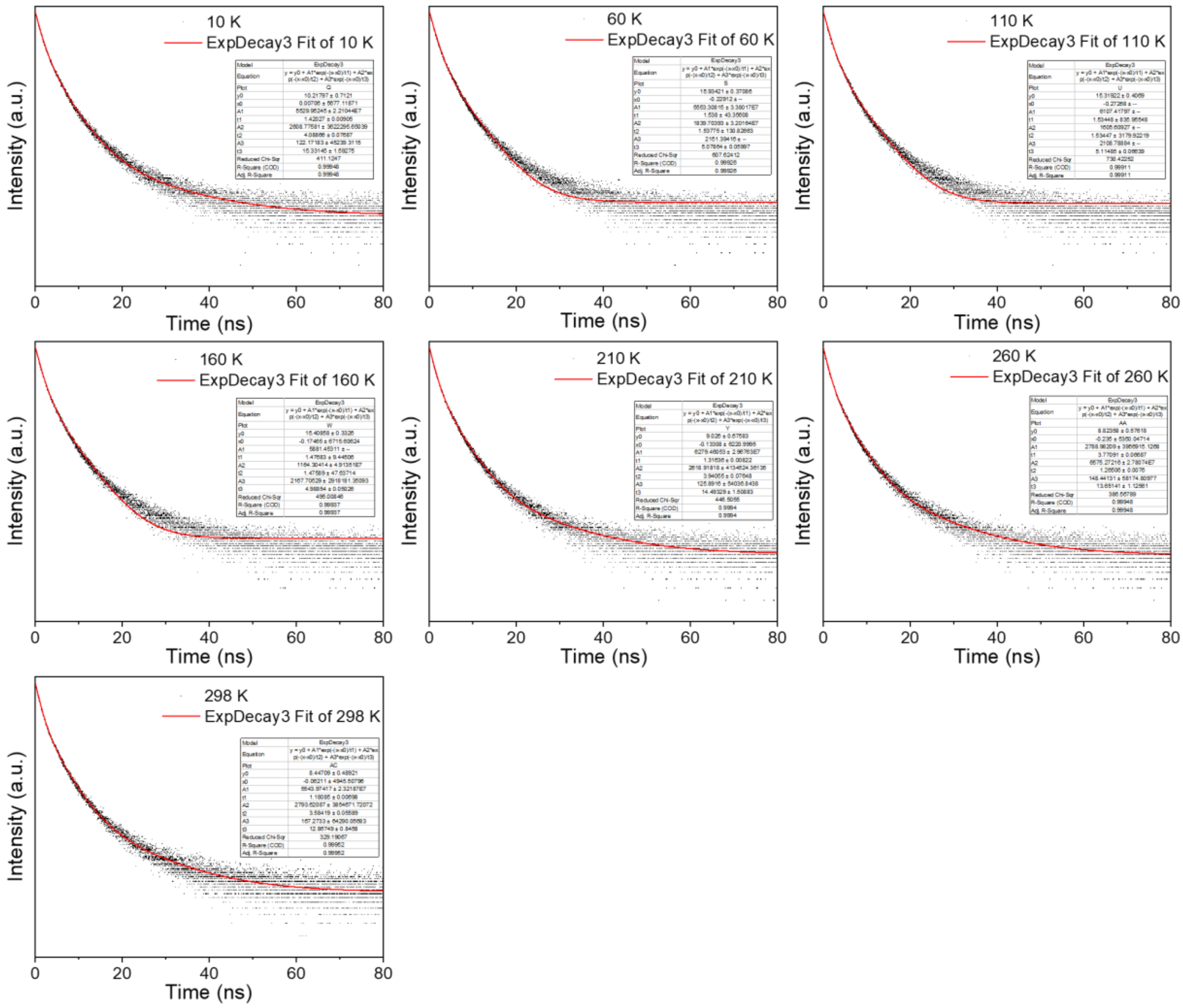


**Figure S42.** ExpDecay3 fitting of the time-luminescence intensity of MOIC-7 glass at different temperatures.

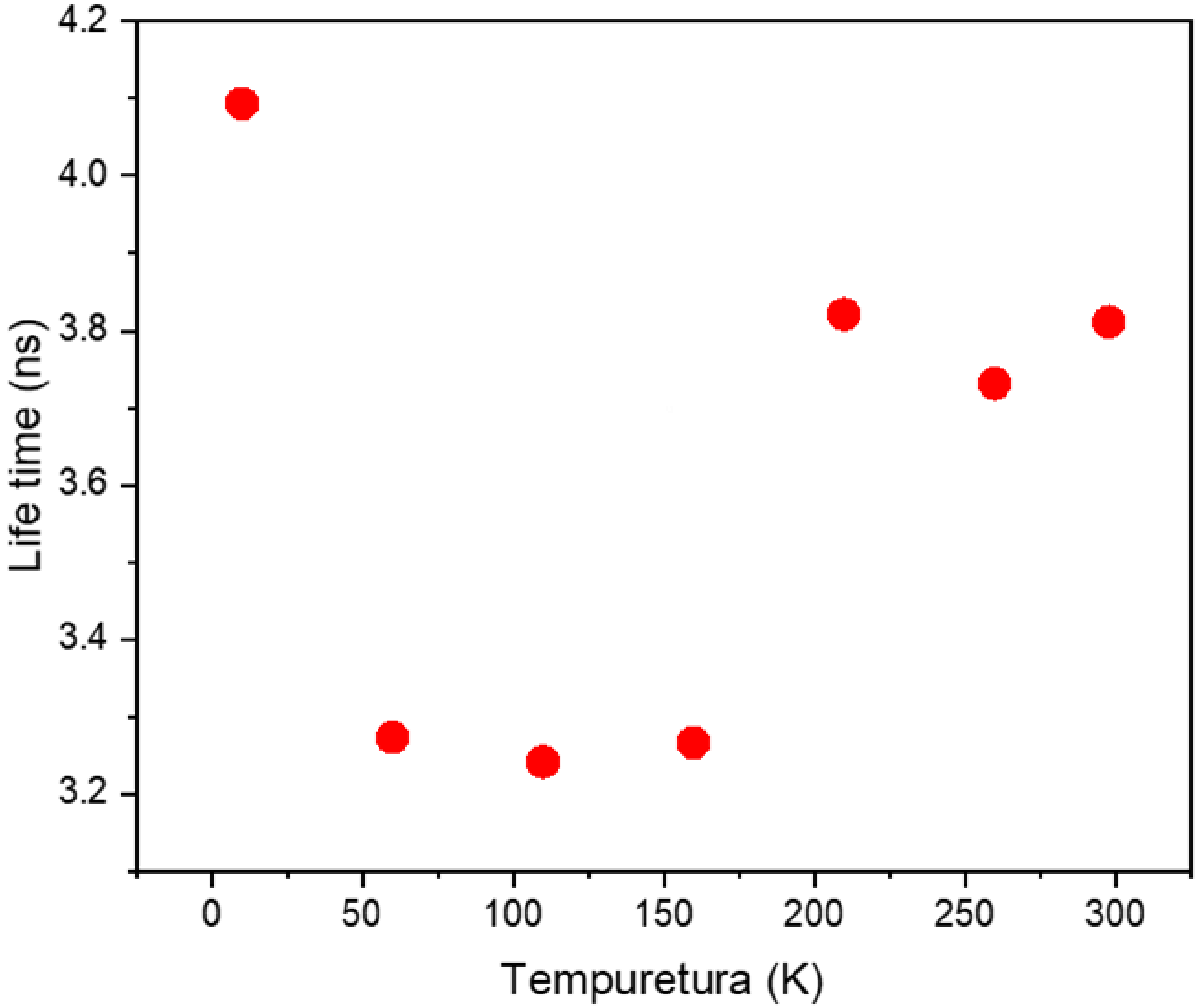


**Figure S43.** Temperature-fluorescence lifetime relationship of MOIC-7 glass.

**Table S1** List of weak interactions in the MOIC-7 crystal structure.

| Type | Kind | Location |
|---|---|---|
| 1 | N-H···Cl (H-bond) | 1D chain + Binding 1D chain to 2D sheet |
| 2 | C-H···Cl (H-bond) | Interdigitation |
| 3 | π-π interaction | Interdigitation + Interdigitated layer packing |
| 4 | H-π interaction | Interdigitation |

Intensity order: 1 > 2 > 3 ~ 4

**Table S2** Thermodynamic data of Zn-MOIC binary eutectic system.

| Sample | $T_m$ (K) | $T_g$ (K) | $T_g/T_m$ |
|---|---|---|---|
| $ZnCl_2(HIm)_2$ | 440 | 281 | 0.64 |
| $ZnCl_2(HIm_{0.916}HbIm_{0.084})_2$ | 432 | 289 | 0.67 |
| $ZnCl_2(HIm_{0.877}HbIm_{0.123})_2$ | 431 | 292 | 0.68 |
| $ZnCl_2(HIm_{0.751}HbIm_{0.249})_2$ | 415 | 303 | 0.73 |
| $ZnCl_2(HIm_{0.501}HbIm_{0.499})_2$ | 476 | 323 | 0.68 |
| $ZnCl_2(HIm_{0.115}HbIm_{0.885})_2$ | 500 | 351 | 0.70 |
| $ZnCl_2(HIm_{0.088}HbIm_{0.912})_2$ | 501 | 353 | 0.70 |
| $ZnCl_2(HbIm)_2$ | 507 | 359 | 0.71 |

**Table S3** Deconvolution parameters for the $^{1}H$ NMR spectrum of the MOIC-7 crystal.

| MOIC-7 crystal | Position (ppm) | FWHM (ppm) |
|---|---|---|
| Peak 1 | 1.84 ± 0.004 | 0.60 ± 0.01 |
| Peak 2 | 4.37 ± 0.01 | 2.14 ± 0.04 |
| Peak 3 | 6.74 ± 0.007 | 2.49 ± 0.03 |
| Peak 4 | 8.48 ± 0.007 | 2.70 ± 0.01 |
| Peak 5 | 12.64 ± 0.004 | 1.04 ± 0.01 |

**Table S4** Deconvolution parameters for the $^{1}H$ NMR spectrum of the MOIC-7 glass.

| MOIC-7 glass | Position (ppm) | FWHM (ppm) |
|---|---|---|
| Peak 1 | 7.17 ± 0.002 | 6.34 ± 0.005 |
| Peak 2 | 7.28 ± 0.0008 | 2.96 ± 0.003 |
| Peak 3 | 12.36 ± 0.002 | 2.21 ± 0.005 |

**Table S5** Deconvolution parameters for the *T*(r) curve of the MOIC-7 crystal.

| MOIC-7 crystal | Fitting peak position (Å) | Original peak position (Å) |
|---|---|---|
| Peak 1 (C-C(N)) | 1. 42 | 1.4 |
| Peak 2 (Zn-Cl) | 2.25 | 2.2 |
| Peak 3 (Zn-C) | 2.98 | 2.95 |
| Peak 4 (Cl-Cl) | 4.16 | 4.1 |
| Peak 5 (Zn-N) | 5.27 | 5.2 |
| Peak 6 (Zn-Zn or Cl-Cl) | 7.51 | 7.5 |
| Peak 7 (Zn-Zn) | 9.0 | 8.8 |

**Table S6** Deconvolution parameters for the *T*(r) curve of the MOIC-7 glass.

| MOIC-7 glass | Fitting peak position (Å) | Original peak position (Å) |
|---|---|---|
| Peak 1 (C-C(N)) | 1. 45 | 1.4 |
| Peak 2 (Zn-Cl) | 2.25 | 2.2 |
| Peak 3 (Zn-C) | 2.98 | 2.95 |
| Peak 4 (Cl-Cl) | 4.17 | 4.1 |

**Table S7** Thermodynamic data of M-MOIC crystals.

| Sample | $T_m$ (K) | $T_g$ (K) | $T_g/T_m$ |
|---|---|---|---|
| $CoCl_2(HIm)_2$ | 446 | 280 | 0.63 |
| $CoCl_2(HbIm)_2$ | 512 | 359 | 0.7 |
| $Co(SCN)_2(HbIm)_2$ | 464 | 349 | 0.75 |
| $Cu(SCN)_2(HIm)_2$ | 446 | 255 | 0.57 |

**Table S8** PLQY of MOIC crystals and MOIC-7 glass. The excitation light wavelength is 370 nm.

| Sample | Absorption efficiency (%) | Internal PLQY (%) | External PLQY (%) |
|---|---|---|---|
| MOIC-4 | 31.19 | 18.04 | 5.63 |
| $ZnCl_2(HIm_{0.877}HbIm_{0.123})_2$ | 28.97 | 15.43 | 4.47 |
| MOIC-7 | 22.15 | 1.20 | 0.26 |
| MOIC-7 glass | 52.42 | 3.40 | 1.78 |

# References


1. Shaker SA, Khaledi H, Ali HM. Spectroscopic investigations and physico-chemical characterization of newly synthesized mixed-ligand complexes of 2-methylbenzimidazole with metal ions. *Chem Pap* 2011; **65**: 299–307.
2. Sarkar S, Ghosh SR, Brandão P *et al*. Role of imidazole edge to edge supramolecular interaction in the crystal packing of $Cu(II)(SCN^{-})_2(imidazole)_2$ complex: A novel variety of supramolecular interaction revealed by CCDC database analysis and explored through DFT computational studies. *J Mol Struct* 2021; **1227**: 129513.
3. Lu T and Chen Q. Independent gradient model based on Hirshfeld partition: A new method for visual study of interactions in chemical systems. *J Comput Chem* 2022; **43**: 539–55.
4. Lu T and Chen Q. Interaction Region Indicator: A simple real space function clearly revealing both chemical bonds and weak interactions. *Chem:Methods* 2021; **1**: 231–9.
5. Lu T. A comprehensive electron wavefunction analysis toolbox for chemists, Multiwfn. *J Chem Phys* 2024; **161**: 082503.
6. Lu T and Chen F. Multiwfn: a multifunctional wavefunction analyzer. *J Comput Chem* 2012; **33**: 580–92.
7. VandeVJ, Krack M, Mohamed F *et al*. Quickstep: fast and accurate density functional calculations using a mixed Gaussian and plane waves approach. *Comput Phys Commun* 2005; **167**: 103–28.
8. Kühne TD, Iannuzzi M, Del BM *et al*. CP2K: an electronic structure and molecular dynamics software package-Quickstep: efficient and accurate electronic structure calculations. *J Chem Phys* 2020; **152**: 194103.
9. Perdew JP, Burke K, Ernzerhof M. Generalized gradient approximation made simple. *Phys Rev Lett* 1996; **77**: 3865–8.
10. VandeVondele J and Hutter J. Gaussian basis sets for accurate calculations on molecular systems in gas and condensed phases. *J Chem Phys* 2007; **127**: 114105.
11. Grimme S, Antony J, Ehrlich S *et al*. A consistent and accurate ab initio parametrization of density functional dispersion correction (DFT-D) for the 94 elements H-Pu. *J Chem Phys* 2010; **132**: 154104.
12. Grimme S, Ehrlich S, Goerigk L. Effect of the damping function in dispersion corrected density functional theory. *J Comput Chem* 2011; **32**: 1456–65.
13. Yamada H, Nakada K, Takemoto M *et al*. Fully automated measurement system for temperature-dependent X-ray total scattering at beamline BL04B2 at SPring-8. *J Synchrotron Radiat* 2022; **29**: 549–54.
